# Tracking Reactions of Asymmetric Organo‐Osmium Transfer Hydrogenation Catalysts in Cancer Cells

**DOI:** 10.1002/anie.202016456

**Published:** 2021-02-15

**Authors:** Elizabeth M. Bolitho, James P. C. Coverdale, Hannah E. Bridgewater, Guy J. Clarkson, Paul D. Quinn, Carlos Sanchez‐Cano, Peter J. Sadler

**Affiliations:** ^1^ Department of Chemistry University of Warwick Coventry CV4 7AL UK; ^2^ I14 Imaging Beamline Diamond Light Source Oxford OX11 0DE UK; ^3^ Center for Cooperative Research in Biomaterials (CIC biomaGUNE) Basque Research and Technology Alliance (BRTA) Paseo de Miramon 182 20014 San Sebastián Spain

**Keywords:** anticancer catalysts, bioorganometallic chemistry, X-ray fluorescence, organo-osmium complexes, transfer hydrogenation

## Abstract

Most metallodrugs are prodrugs that can undergo ligand exchange and redox reactions in biological media. Here we have investigated the cellular stability of the anticancer complex [Os^II^[(η^6^‐*p*‐cymene)(*RR*/*SS*‐MePh‐DPEN)] [**1**] (MePh‐DPEN=tosyl‐diphenylethylenediamine) which catalyses the enantioselective reduction of pyruvate to lactate in cells. The introduction of a bromide tag at an unreactive site on a phenyl substituent of Ph‐DPEN allowed us to probe the fate of this ligand and Os in human cancer cells by a combination of X‐ray fluorescence (XRF) elemental mapping and inductively coupled plasma‐mass spectrometry (ICP‐MS). The BrPh‐DPEN ligand is readily displaced by reaction with endogenous thiols and translocated to the nucleus, whereas the Os fragment is exported from the cells. These data explain why the efficiency of catalysis is low, and suggests that it could be optimised by developing thiol resistant analogues. Moreover, this work also provides a new way for the delivery of ligands which are inactive when administered on their own.

## Graphical abstract

This chiral organo‐osmium complex catalyses enantioselective reduction of pyruvate to lactate in cancer cells. The bromine tag on the chelated ligand and osmium have been jointly mapped in cells by X‐ray fluorescence and inductively coupled plasma‐mass spectrometry, revealing facile displacement of the ligand and its translocation to the nucleus.

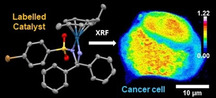

## Introduction

Transition metal catalysts have potential as therapeutic agents to treat cancer and other diseases.^[1]^ Such catalysts might transform multiple substrate molecules in situ, including exogenous prodrugs, diagnostic agents, and endogenous metabolites,[^1^, ^2^] whilst requiring only low concentrations to achieve the desired activity. Therapeutic strategies based on metal catalysts might help to overcome resistance to chemotherapy and reduce unwanted side effects,[^1a^, ^1b^] both of which are of current clinical concern.

**Figure 1:**
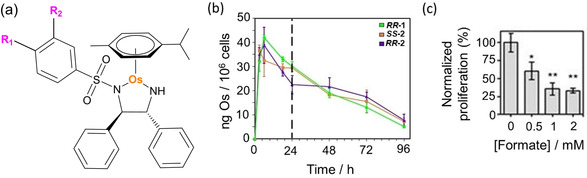
a) Structures of [Os(*p*‐cymene)(R_1/2_Ph‐DPEN)] complexes **1**–**5** (for R_1_ and R_2_ substituents, see Table 1). Only the (*R*,*R*) configuration is shown for clarity. b) Accumulation of Os (ng/10^6^ cells) in A2780 (human ovarian) cancer cells treated with 1×IC_50_ of ***R***,***R*** **‐1**, ***S***,***S***‐**2** and ***R***,***R***‐**2** (310 K, 5 % CO_2_) for 3, 6, 18 or 24 h (no recovery) or 24 h drug exposure followed by 24, 48 or 72 h recovery period (in complex‐free media). c) Transfer hydrogenation‐modulated cellular viability of A549 (human lung) cancer cells treated with 0.5×IC_50_ of ***S***,***S*** **‐2** in the presence of formate (0–2 mM). Statistical significance was calculated using a two‐tailed t‐test assuming unequal variances (Welch's t‐test), *p<0.05 and **p<0.01.

[Os^II^[(η^6^‐*p*‐cymene)(*RR*/*SS*‐MePh‐DPEN)] [**1**] (MePh‐DPEN=tosyl‐diphenylethylene‐diamine) is a chiral 16‐electron organo‐osmium(II) half‐sandwich complex structurally derived from the well‐established Noyori Ru^II^ catalysts (Figure 1 a),^[3]^ which shows high enantioselectivity and conversion rates. For example, reduction of acetophenone is ca. 3‐fold more efficient (in turnover frequency, TOF) and more stable (over one month under normal atmospheric conditions) than its industrially‐used Ru^II^ analogue.^[3]^ Furthermore, once inside cells, and in presence of the non‐toxic hydride donor formate, this complex catalyses the enantioselective reduction of pyruvate, an essential precursor in cell metabolism, to natural L‐lactate or unnatural D‐lactate, depending on the chirality of the catalyst.^[1f]^

It can be assumed that the ability of such catalysts to cause metabolic perturbations in cells requires the presence of intact catalyst, which contributes to the antiproliferative activity and selectivity of **1** towards a variety of human cancer cell lines.[^2e^, ^4^] Yet, its intracellular catalytic activity is most likely marked by low turnover numbers (TON; previously estimated to be ≈13), which might suggest some degradation of the complex inside cells.^[1f]^ This might be expected to be a common problem for synthetic metal catalysts, such as organometallic complexes designed to work under well‐defined chemical conditions, including inert atmospheres and in organic solvents.[^1g^, ^1i^, ^2c^, ^5^] In order to optimise the design of synthetic intracellular catalysts, and increase their in cellulo catalytic and biological efficiency, it is important to investigate their fate in cells.

Previous work using ICP‐MS experiments on fractionated cancer cells treated with **1**, showed a ca. 47 % cytosolic accumulation of the Os,^[1f]^ suggesting that catalysis may take place in the cytosol. Additionally, ca. 48 % of intracellular Os was present in the membrane/ particulate fraction (which contains organelles and membrane proteins), which may also implicate organelles (i.e. mitochondria or lysosomes) as cellular targets.^[1f]^ However, such studies did not provide information on the intracellular stability of the complex.

To probe this, we have incorporated a halide substituent on a sulfonylphenyl substituent in the chelated Ph‐DPEN ligand, so generating [Os^II^(η^6^‐*p*‐cym)(R_1/2_Ph‐DPEN)], complexes with R_1_(*para*)=Br (complexes ***R***,***R*** **‐** and ***S***,***S*** **‐2**), I (***R***,***R*** **‐** and ***S***,***S*** **‐3**), F (***R***,***R*** **‐4**), or R_2_(*meta*)=Br (***S***,***S*** **‐5**). A combination of nanofocused synchrotron X‐ray fluorescence (XRF) and ICP‐MS allows not only osmium but also the chelated DPEN ligand to be tracked in cells using the halide label. These experiments shed new light on the chemistry of these organometallic catalysts in cells, and will aid the design of next‐generation catalytic drugs.

## Results and Discussion

We introduced halogen tags (R_1_ or R_2_) into chiral Ph‐diphenylethylene (Ph‐DPEN) ligands by coupling phenylsulfonyl chlorides carrying Br, I, or F in *para* or *meta* positions to 1*S*,2*S* or 1*R*,2*R* diphenylethylenediamine. The new ligands were then reacted with [Os(*p*‐cymene)Cl_2_]_2_ dimer (synthesised using a reported method)^[6]^ to generate enantiomerically pure organometallic complexes ***S***,***S*** **‐** and ***R***,***R*** **‐2** (*p*‐Br) and **3** (*p*‐I), ***R***,***R*** **‐4** (*p*‐F), and ***S***,***S*** **‐5** (*m*‐Br), analogues of complex **1** (Figure 1 a).

Halogenation of the phenyl group did not alter significantly the structure of the complexes, as seen by comparison of the X‐ray crystal structures of complexes ***S***,***S*** **‐1**, ***S***,***S*** **‐2** and ***S***,***S*** **‐3** (Supporting Information, Table S1, Figures S7 and S8), and led only to a moderate decrease in their hydrophobicity (Log*P* 1.45±0.02 for **1**, ca. 1 for **2**, **3** and **5**, and 0.30±0.03 for **4**; Supporting Information, Table S2). Density Functional Theory (DFT) calculations related such changes in hydrophobicity to differences in the effective charge distribution at the C−X bonds due to the electronegativity of the halides (F> Cl > Br > I, Supporting Information, Table S3, Figure S9). However, no significant effects were observed on the charge at the Os center, *p*‐cym arene, or N atoms of the R_1/2_Ph‐DPEN ligand (Supporting Information, Tables S3–6). Equally, **2**–**5** maintained their ability to act as catalysts for asymmetric transfer hydrogenation (ATH) reactions. They catalysed the reduction of acetophenone to (*S*)‐ or (*R*)‐1‐phenylethanol in presence of formic acid, achieving high enantiomeric excesses and conversions (>93 %), with the Br and I analogues (**2**, **3**) showing slightly higher catalytic efficiency when compared with **1** (TOF=81±3, 87±5 and 63.6±0.6 h^−1^,^[3]^ respectively; Table 1). This confirmed that labelling Ph‐DPEN ligands with halogen tags did not affect significantly the chemical or catalytic properties of the new complexes. Furthermore, ^1^H NMR and UV/Vis studies showed that the halogenated analogues of **1** were stable over 24 h in DMSO or PBS.

**Table 1:** Catalytic conversion (%), enantiomeric excess (%) and turnover frequency (TOF, h^−1^) for the reduction of acetophenone to (*S*)‐ or (*R*)‐1‐phenylethanol for complexes **1**–**5** in the presence of 5:2 formic acid/ TEA azeotrope for 24 h at 310 K as determined by ^1^H NMR and chiral GC. Half‐maximal inhibitory concentrations (IC_50_/μM) of **1**–**5** towards A2780 (human ovarian) and A549 (human lung) cancer cells upon 24 h complex exposure followed by a 72 h recovery period in fresh media. Osmium cellular accumulation in A2780 cells treated with 1×IC_50_ of **1**–**4** for 24 h (no recovery).

	R_1_	R_2_	S/C	Conv. [%]^[a]^	Config.^[b]^	*ee* [%]^[b]^	TOFh^−1^)^[a]^	A2780 IC_50_^[c]^	Os ng/10^6^ A2780 cells^[d]^	A549 IC_50_^[c]^
***S***,***S*** **‐1** ^[e]^	CH_3_	H	200	99	S	99	63.9±0.3	15.2±0.5	32±3	21.1±0.3
***R***,***R*** **‐1** ^[e]^	CH_3_	H	200	99	R	99	63.6±0.6	15.5±0.5	30±2	31±1
***S***,***S*** **‐2**	Br	H	200	98	S	98	81±5	31±2	29±3	29.5±0.5
***R***,***R*** **‐2**	Br	H	200	99	R	97	81±2	27.4±0.6	30±2	33±0.4
***S***,***S*** **‐3**	I	H	200	96	S	94	88±5	27.5±0.8	39±2	32±0.4
***R***,***R*** **‐3**	I	H	200	97	R	95	85±5	29±3	37±3	–
***R***,***R*** **‐4** ^[e]^	F	H	200	99	R	96	40±1	17±1	10±2	32±2
***S***,***S*** **‐5**	H	Br	200	98	S	98	79±4	27±2	–	21.1±0.3

[a] Determined by ^1^H NMR. [b] Determined by chiral GC. [c] Determined using SRB colorimetric assay. [d] Determined by ICP‐MS. [e] Previously reported data for ***S,S**‐**1***, ***R,R**‐**1*** and ***R***,***R***‐**4**.[^1f^, ^3^]

Enantiomers of **2** and **3** showed the same antiproliferative activity in vitro against ovarian A2780, lung A549, breast MCF7 and prostate PC3 cancer cells, while ***R***,***R*** **‐4** was more potent against A2780 cells. Still, all of them were slightly less potent than **1**. (Figure 1 d; Supporting Information, Table S7). Similarly, they were also less toxic to zebrafish embryos, and showed slightly improved biocompatibility (Supporting Information, Table S20).^[4]^ ICP‐MS showed similar amounts of Os in cells treated with equipotent concentrations of **1**–**3** after 24 h (dose of 1×IC_50_, Figure 1 d; 0.25–1.5×IC_50_, Supporting Information, Tables S10 and S11). Moreover, identical time‐dependent influx and efflux of osmium was observed in cells treated with 1×IC_50_ of **1** and **2** (Figure 1 b); reaching maximum accumulation after ca. 6–8 h followed by a period of concentration‐independent efflux. Both complexes were also taken up to some extent by cells using energy‐dependent mechanisms (i.e. endocytic pathways leading to lysosomal deposition), as accumulation of intracellular osmium decreased at 277 K (Supporting Information, Table S12). Reduction in accumulation was significantly lower for cells treated with **2**.

The anticancer activity of ***R***,***R*** **‐** and ***S***,***S***‐**2** was improved (cell survival reduced by 74 %) by co‐treatment with non‐toxic concentrations of hydride donor formate (Figure 1 c; Supporting Information, Figure S10), but not by acetate, which cannot act as a hydride donor (Supporting Information, Figure S11). The effect induced by formate was specific for cancer cells compared to normal healthy cells (Supporting Information, Figure S12), and was not caused by an increase in the intracellular levels of Os (*p*>0.5; Supporting Information, Table S13), or the disruption of membrane integrity (Supporting Information, Table S8, Figure S13). However, as previously observed for **1**,^[1f]^ the presence of **2** induced G_1_‐arrest (Supporting Information, Table S9). These results confirm that halogen substitution does not affect significantly the chemical, structural and catalytic properties of **2**–**5** compared to **1**. The tagged complexes also show similar biological properties to the parent complex. Complexes **2**–**5** appear to have the same mechanism of action than **1**, being capable of performing in‐cell transfer hydrogenation. The small decrease in their anticancer potency may be be caused by reduced cellular accumulation, possibly related to lower hydrophobicity.

The halogen tags used (i.e. F, Br and I) are present in most biological organisms.^[7]^ Bromine is reported to have an important role in connective tissue formation,^[8]^ but is found in lower quantities than the other halogens in the body.^[7a]^ Bromine is also present only at low concentrations in formulations for cell culture media and serum albumin.[^7a^, ^9^] Thus, low backgrounds were found when its accumulation and distribution were determined in cellular samples that were cultured in vitro (4.6 ng Br/ 10^6^ cells). Furthermore, due to the relatively high stability towards nucleophilic substitution of the C−Br bond in the BrPh‐DPEN ligand in **2**,^[10]^ release of free bromide is unlikely to occur in the presence of biological thiols in cellular conditions. Therefore, most of the detected bromine should correspond to either complex **2** itself, displaced or fragmented BrPh‐DPEN ligand. Hence, we used brominated complex **2** to probe the stability of this catalyst in cells by determining the relative accumulation and localisation of Os and the Br tag, by detection of ^189^Os and ^79^Br by ICP‐MS, and by elemental mapping of Os and Br by detecting nanofocused synchrotron X‐ray fluorescence (XRF) emissions of Os (L_3_‐M_5_=8.9 keV) and Br (K‐L_3_=11.9 keV).

**Figure 2:**
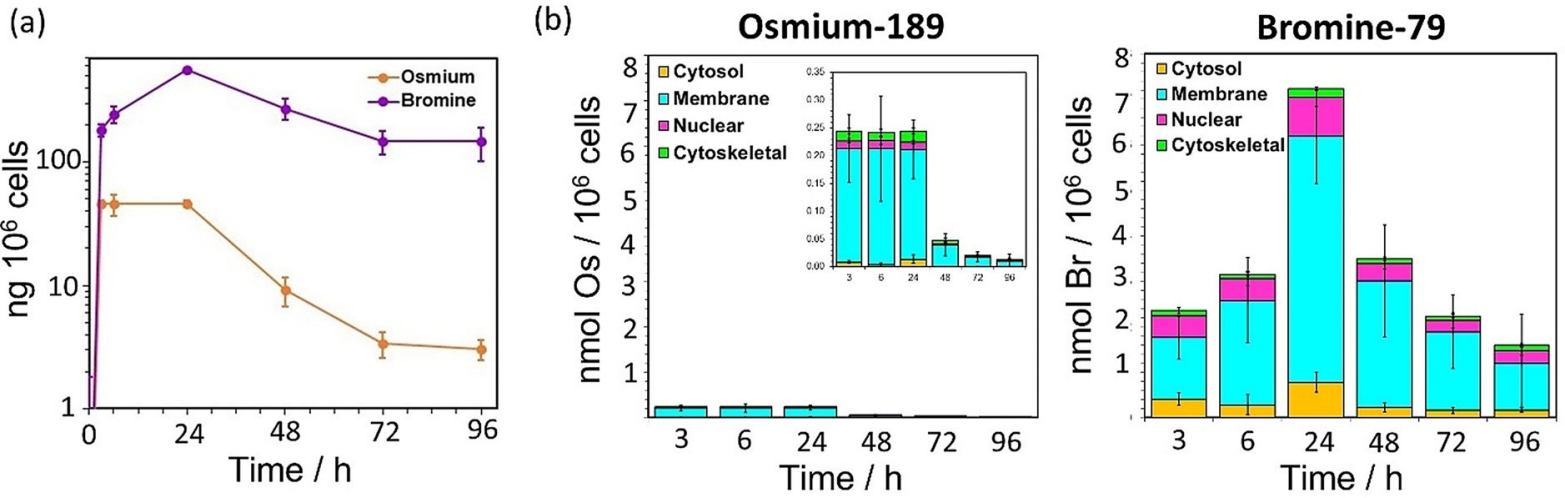
a) Time‐dependent accumulation of Os (^189^Os) and Br (^79^Br) in A549 (human lung) cancer cells treated with 1×IC_50_ (30 μM) of ***S***,***S*** **‐2** for 3, 8 18 or 24 h (no recovery) or 24 h followed by 24, 48 and 72 h recovery in drug‐free medium, shown on a logarithmic scale for comparison. b) Time‐dependent accumulation of Os (^189^Os) and Br (^79^Br) in sub‐cellular fractions (cytosol, membrane, nuclear and cytoskeletal) of A549 cells treated with 1×IC_50_ (30 μM) of ***S***,***S*** **‐2** for various exposure times as quantified by ICP‐MS.

Quantification of Br by ICP‐MS was challenging due to the high energy of its first ionisation potential (11.8 eV),^[11]^ and the presence of common polyatomic interferences with *m*/*z*=79 or 81 (e.g. ^40^Ar^39^K^+^ or ^31^P^16^O_3_^+^ for *m*/*z*=79; ^32^S^16^O_3_^1^H^+^ or ^40^Ar^40^Ar^1^H^+^ for *m*/*z*=81).^[12]^ Furthermore, nitric acid could not be used as the cell digestion matrix, as it induces oxidation of bromine to molecular Br_2_. Instead, pellets of A549 lung cancer cells treated with ***S***,***S*** **‐2** under different conditions were digested at 353 K using an alkaline solution of 25 % *w*/*v* tetramethylammonium hydroxide (TMAH). This reduced the loss of volatile analytes.^[13]^ As expected, intracellular levels of Br were much lower for untreated cells than in cells exposed to ***S***,***S*** **‐2** (4.6 vs. 185 ng/10^6^ cells after exposure to 30 μM of ***S***,***S*** **‐2** for 0 or 3, h, respectively). The cells also accumulated Br and Os in a different time‐dependent manner (Figure 2 a). The highest levels of intracellular Os were reached after ca. 4–8 h, and the levels of both Os and Br decreased upon recovery of cells in drug‐free media after 24 h exposure (Figure 2 a; Supporting Information, Table S14). However, there was a clear influx of Br into cells for the whole duration of the exposure to the complex (24 h). The Br tag in the complex was accumulated >9× more (on a molar basis) than Os after just 3 h exposure to ***S***,***S***‐**2** (Figure 2 a; Supporting Information, Table S14).

ICP‐MS measurements using isolated subcellular fractions obtained from cells treated with ***S***,***S*** **‐2** also showed clear differences in the cellular distribution of Br and Os (Figure 2 b; Supporting Information, Tables S15–19). Osmium was mostly (>65 %) in the fraction containing the membranes and other organelles (i.e. mitochondria or lysosomes) at all of the time points analysed (3–96 h). Lesser amounts of Os were found in the cytoskeletal fraction, perhaps indicating interactions between cationic osmium species and negatively‐charged microtubules, while very little was present in the cytosol and nuclei of the treated cells (<6 %). In contrast, while most Br was always present in the membrane and organelle fraction (>58 %), much higher quantities of Br compared to Os were found at all times in the cytosol and nuclei of the treated cells (10–20 %).

ICP‐MS experiments showed that not only the levels of Os, but also Br decreased when cells were exposed to ***S***,***S*** **‐2** at lower temperatures (277 K vs. 310 K, by ca. 48 % and 75 %, respectively; Supporting Information, Figure S15). Lower Br‐to‐Os ratios found in cells upon inhibition of active transport also suggested the presence of higher quantities of intact complex (Supporting Information, Figure S16). Besides, excretion of Os and BrPh‐DPEN seemed to occur through different mechanisms. Inhibition of caveolae endocytic pathways using methyl‐β‐cyclodextrin reduced cellular uptake of the complex (Supporting Information, Figure S17 and S18). Yet, it did not alter the intracellular levels of Br, possibly by inhibiting the cellular efflux of the free ligand. Moreover, Os (but not Br) efflux was reduced when cells recovered in 20 μM of verapamil after exposure to ***S***,***S***‐**2** (Supporting Information, Tables S18,19, Figure S19). Verapamil inhibits the ATP‐dependent efflux membrane pump Pgp (Permeability Glycoprotein‐1; a well‐known pump involved in detoxification and drug resistance).^[14]^ Thus, uptake experiments suggested the presence of intracellular degradation of the complex, followed by differential cellular trafficking and efflux for Os and Br‐carrying fragments.

**Figure 3:**
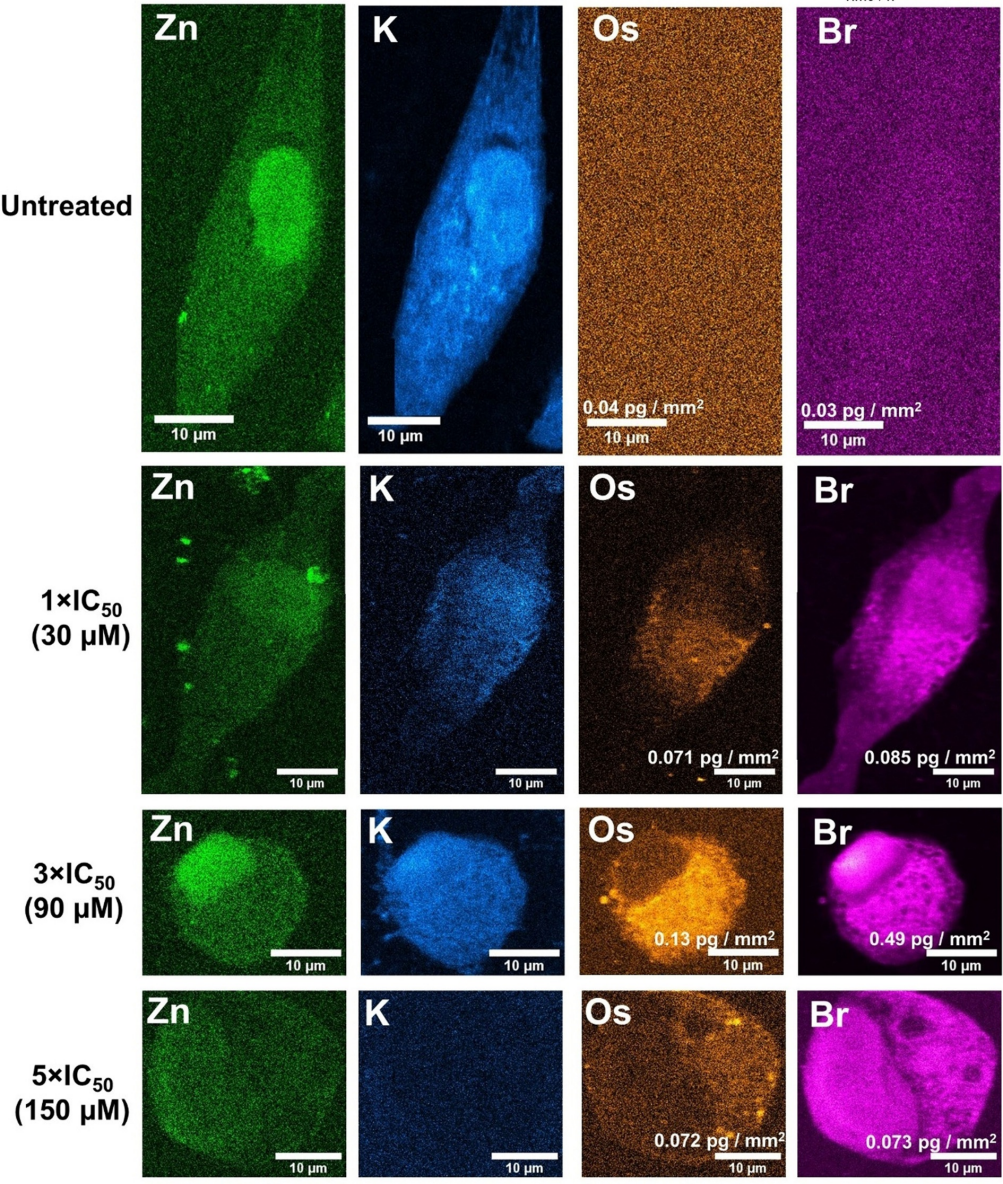
XRF elemental maps acquired from cryo‐fixed and freeze‐dried A549 (human lung) cancer cells treated with 1–5×IC_50_ (30–150 μM) of ***S***,***S*** **‐2** for 24 h showing the distribution of: Zn (**green**); (ii) K (**blue**) Os (**orange**) and Br (**pink**). Maps were collected using 100 nm step size and 0.1 s dwell time. Data were processed in PyMca toolkit developed by the ESRF,^[15]^ and images generated in ImageJ.^[16]^

It is difficult to differentiate between membrane‐bound and internalized elements using 2D mapping techniques. Still, concentration of large quantities of such elements in specific organelles and cellular areas (i.e. nuclei, lysosomes, mitochondria, ER or other cytosolic organelles) can be easily detected, providing vital information on their cellular distribution. As such, the distribution of Os and Br in cancer cells was further studied at sub‐cellular spatial resolution (100×100 nm^2^) by acquiring XRF elemental maps using nanofocused synchrotron radiation at I14 (Diamond Light Source). A549 lung cancer cells grown on silicon nitride membranes were treated with various concentrations of ***S***,***S*** **‐2** (1–5× IC_50_ concentration) for 24 h, before being cryo‐fixed and freeze‐dried for subsequent analysis under ambient conditions (Figure 3). Natural intracellular levels of Br were below the detection limit (Supporting Information, Figure S20). XRF emissions from Br or Os were not detected in untreated cells (Supporting Information, Figure S21–24). These control cells also maintained a normal “stretched out” morphology (as seen from S, P and K maps) typical of this cell line,^[17]^ and had clearly defined nuclei, areas with high accumulation of Zn (Supporting Information, Figures S21–24). On the contrary, XRF maps acquired from cells treated with ***S***,***S*** **‐2** showed the presence of drug‐induced morphological changes, which were concentration‐dependent (Figure 3; Supporting Information, Figures S25–S38). For example, cells became smaller in size and more rounded in shape when treated with 1–3×IC_50_ of ***S***,***S*** **‐2**. Instead, the use of higher concentrations of the drug (5×IC_50_) led to cell swelling (Figure 3; Supporting Information, Table S21, Figures S39–42) and nuclei with increased size and poorly‐defined perimeters due to a sparse intracellular distribution of Zn, suggesting rupture of the nuclear membrane. This indicated significant concentration‐dependent cell damage caused by **2**.

**Figure 4:**
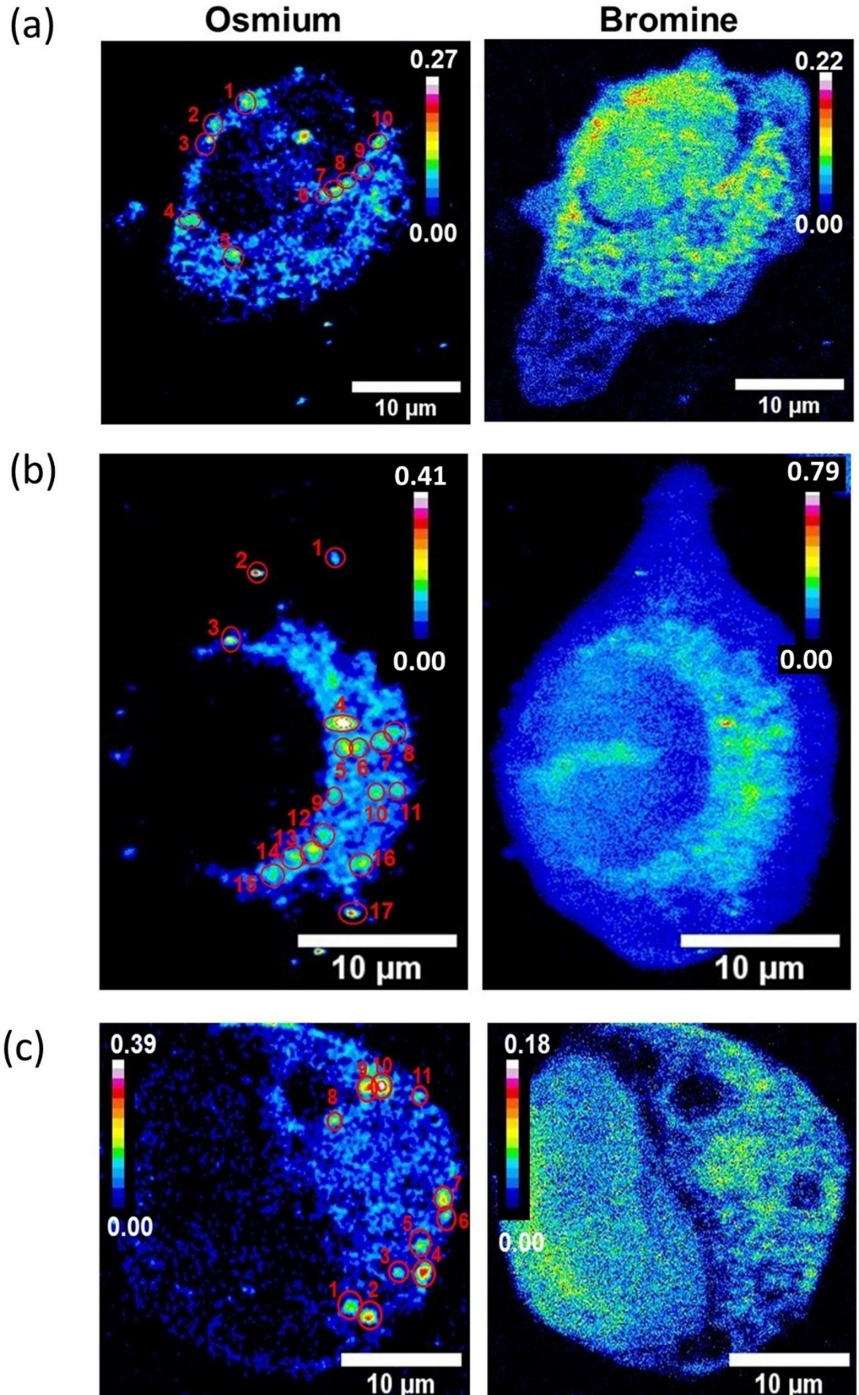
XRF elemental maps of Os and Br acquired from cryo‐fixed and freeze‐dried A549 (human lung) cancer cells treated with a) 1, b) 3 or c) 5×IC_50_ of ***S***,***S*** **‐2** (30, 90 or 150 μM) for 24 h, showing Os‐Br co‐localisation in small vesicle‐like areas. Maps were collected using 100 nm step size and 0.1 s dwell time. The calibration bar is in pg mm^−2^. Data were processed using the PyMCa toolkit developed by the ESRF,^[15]^ and images generated in ImageJ.^[16]^

XRF elemental maps obtained from cells treated with ***S***,***S*** **‐2** also showed that Os was located mostly in cytosolic regions (from 75–85 % for 1–3×IC_50_ treatment, to ca. 65 % at 5×IC_50_; Figure 3, Supporting Information, Table S22, Figures S25–38). Equally, cells treated with the complex accumulated more Br than Os (5, 7 and 2× more at 1, 3 and 5×IC_50_, respectively; Supporting Information, Table S23). Most of that intracellular Br was found in regions of the cytosol (71±9 %, 60±7 % and 52±7 % at 1, 3 and 5×IC_50,_ respectively), but a significant amount of Br also reached the nuclei of treated cells (29±9 %, 40±7 % and 48±7 % at 1, 3 and 5×IC_50_, respectively; Figure 3, Supporting Information, Table S23). Remarkably, the percentage of Br in the nuclei calculated from XRF maps (29±9 %) was slightly higher than that found when nuclear fractions were isolated from cells treated under the same conditions (1×IC_50_) and analyzed using ICP‐MS (12±1 %). XRF data are based on averaged elemental determinations from between 3–7 individual cryo‐fixed, dried cells, in contrast to ICP‐MS, performed on digested cell fractions from a large population of cells. Also, fractionation kits can introduce elemental leeching and cross‐contamination between fractions. Moreover, the data obtained from XRF maps confirmed the trends found on our previous ICP‐MS experiments, and implied that intracellular degradation of the complex was occurring. Nevertheless, the Os and Br from the catalyst were found to co‐localise moderately in the cytoplasm (Pearson's Coefficient *R*=0.24±0.11, 0.39±0.05 and 0.17±0.01 at 1, 3 and 5×IC_50_, respectively; Supporting Information, Table S24). Thus, suggesting that some of the complex should remain intact in those areas after 24 h treatment, and could support transfer hydrogenation catalysis in the cytosol of cancer cells. Furthermore, both Os and Br appeared to accumulate in small (0.65±0.21 μm^2^, 0.6±0.1 μm^2^ and 0.78±0.25 μm^2^ at 1, 3 and 5×IC_50,_ respectively), cytoplasmic compartments of cells when they were treated with ***S***,***S*** **‐2** (Figure 4 a–c; Supporting Information, Table S25, Figures S43–53). It is likely that these are lysosomes or endosomes, since they are known to be similar in size, and temperature‐dependent accumulation experiments had shown (at least) partial uptake of the Os catalysts through endocytosis (or other active transport mechanisms). Remarkably, Br/Os ratios were lower in those areas than in the rest of the cell (4±2, 4±1 and 1.48±0.17 at 1, 3 and 5×IC_50_, respectively, Supporting Information, Tables S23 and S25), suggesting the presence of higher concentrations of intact complex.

**Figure 5:**
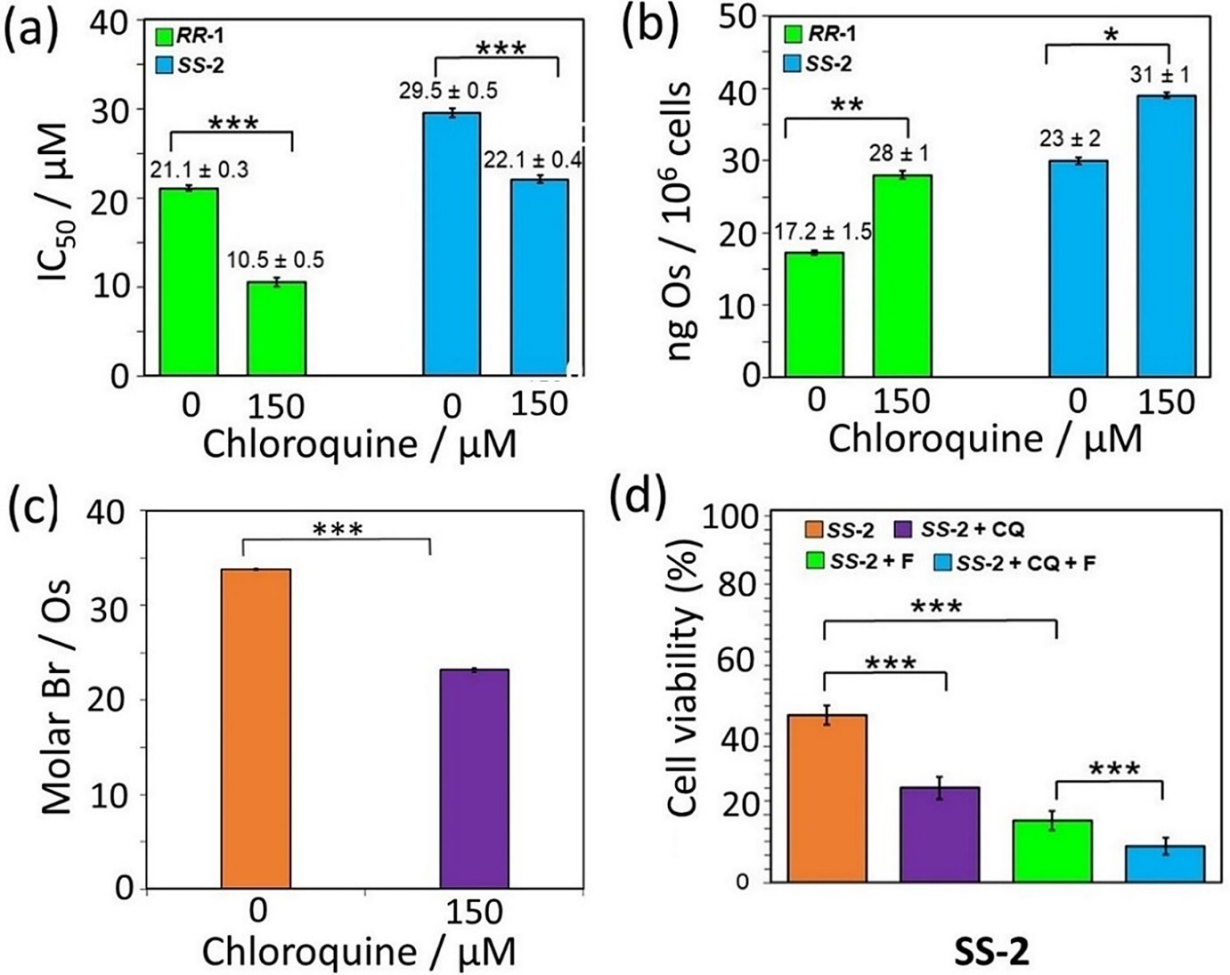
a) Half‐maximal inhibitory concentrations (IC_50_/μM) of ***R***,***R*** **‐1** and ***S***,***S*** **‐2** for A549 (human lung) cancer cells pre‐incubated with 0 or 150 μM of chloroquine diphosphate for 2 h, followed by 24 h complex exposure and 72 h recovery period in fresh media. b) Accumulation of Os (^189^Os; ng/10^6^ cells) in A549 cells pre‐incubated with 0 or 150 μM chloroquine diphosphate for 2 h and treated with 1×IC_50_ of ***R***,***R*** **‐1** (21 μM) or ***S***,***S‐*** **2** (30 μM) for 24 h. c) Molar Br‐to‐Os ratios (Br/Os) for A549 cells pre‐incubated with 0 or 150 μM of chloroquine diphosphate for 2 h and treated with 1×IC_50_ of ***S***,***S*** **‐2** (30 μM) for 24 h. d) Transfer hydrogenation‐modulated cellular viability of human A549 cells pre‐incubated with 0 or 150 μM chloroquine diphosphate for 2 h and treated with 1×IC_50_ of ***S***,***S*** **‐2** in the presence of formate (0–2 mM). Statistical significance was calculated using a two‐tailed t‐test assuming unequal variances (Welch's t‐test), *p<0.05, **p<0.01 and ***p<0.001.

Since the catalysts were at least partially taken up by energy‐dependent mechanisms and probably reached the lysosomes, we investigated whether they are stable in acidic, cysteine‐rich environments typical of lysosomes (i.e. cysteine proteases).[^18^, ^19^] We incubated complexes **1** and **2** for 24 h at pH 5.5 or 7, with 1 or 10 mM L‐cysteine at 310 K. MS analysis showed that the complexes alone remained intact at pH 5.5, but in the presence of L‐Cys they released their chelated MePh‐DPEN or BrPh‐DPEN ligands (Supporting Information, Table S26, Figures S54–S57). The fragment or fragments carrying the Os center remain unidentified, as they could not be isolated. It seems unlikely that dissociation of the Br‐labelled chelated ligand occurs in the culture medium used to treat A549 cells since although we have shown that such a dissociation can be induced by thiols, the level of thiols in the medium is very low, with cysteine being present only as oxidised cystine (0.2 mM) which does not react (Figure S58). Cys34 in the foetal albumin present (ca. 30 μM), which is in a cleft, would be expected to be inaccessible to such a bulky organometallic complex, as found previously for [(biphenyl)Ru(en)Cl]^+^.^[20]^ Thus, degradation of the complexes by thiols such as GSH seems likely to occur in lysosomes after endocytosis. We tested this hypothesis by reducing cellular levels of glutathione with low doses of L‐buthionine sulfoximine (L‐BSO), or inhibiting the activity of lysosomes in A549 cells with chloroquine diphosphate (which reduces activity of lysosomal proteases and prevents endosome maturation).^[21]^ Remarkably, co‐administration of L‐BSO (5 μM; Figure S59) or pre‐incubation with chloroquine (150 μM, 2 h; Figure 5 a) led to a significant increase in the anticancer potency of **1** (by 36 % or 49 %, respectively) and **2** (by 25 %, for chloroquine). This may be due to increased accumulation of catalyst (Figure 5 b), but was not caused by a decrease in membrane integrity in cells exposed to both chloroquine and the catalysts (Supporting Information, Table S27). It appeared that the complexes were degraded less by chloroquine‐treated cells, as they accumulated higher quantities of Os and had lower Br/Os ratios (Figure 5 b,c, Supporting Information, Figure S60, Table S28). Interestingly, chloroquine enhanced the antiproliferative response of **1** to formate, which supported the presence of increased concentrations of intact catalyst inside cells (Figure 5 d, Supporting Information, Table S28).

**Figure 6:**
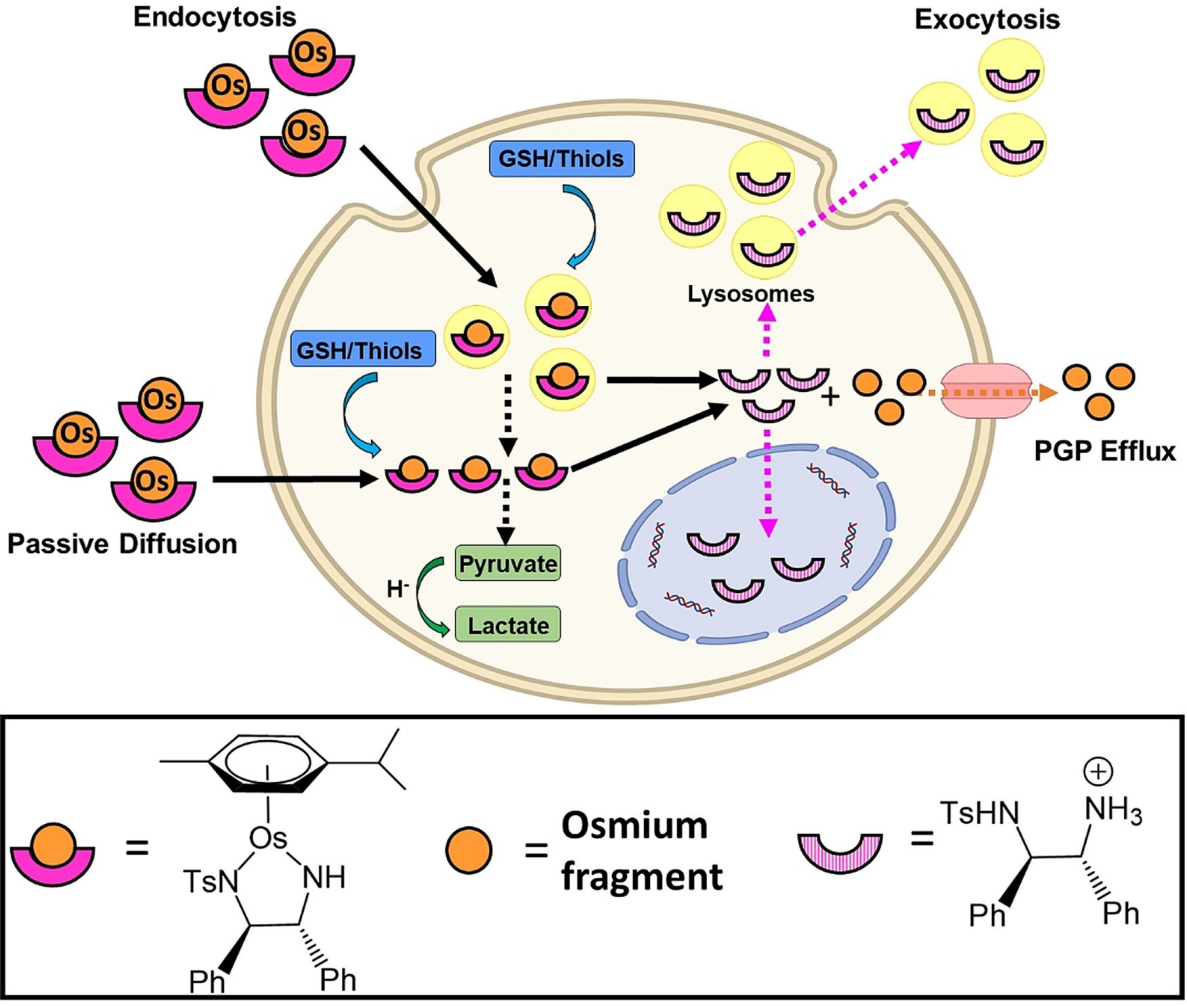
Mechanisms for the uptake of catalyst ***R***,***R‐*** **1** into cancer cells, its degradation and efflux. Created with BioRender.com.

Overall, these results provide new insights into the behaviour of this family of asymmetric transfer hydrogenation catalysts in cancer cells (Figure 6). The complexes are internalised by cells via a combination of both passive and active transport, reaching lysosomes, the cytosol and some other organelles. Once inside, in the presence of a hydride donor, intact catalysts facilitate the catalytic reduction of pyruvate to lactate in the cytosol, altering the metabolism of cancer cells and inhibiting their proliferation. The catalysts also interact with intracellular thiols (e.g. cysteine‐containing peptides such as GSH and proteins). This leads to the release of Os‐containing fragments and intact (or fragmented) DPEN chelating ligands, which are likely to be charged at physiological pH.^[22]^ The fragments generated during this degradation exhibit different cellular behaviour, which explained differences observed between the accumulation of Os and Br. Osmium‐containing fragments are rapidly excreted from cells via Pgp membrane pumps, while chelated ligands show much longer in‐cell lifetimes. As such, RPh‐DPEN ligands are highly accumulated by cells, reaching even the cell nuclei before they are excreted by vesicle‐related exocytosis. This raises questions about the role of the free ligands in the activity of the complexes. However, degradation of **1** and its analogues causes the loss of catalytic and biological activity of the complexes, reducing their overall anticancer potential. Furthermore, although RPh‐DPEN ligands are readily internalised by cells (Supporting Information, Table S15), they do not possess antiproliferative properties (IC_50_>150 μM, Supporting Information, Table S7). Still, the interaction observed between **1** and cellular thiols could provide a basis for developing analogous therapeutic complexes designed for the simultaneous delivery of active ligands and reactive fragments of metal complexes inside cells.

## Conclusion

We have labelled chelated ligands of anticancer organo‐osmium transfer hydrogenation catalysts with halogen atoms. Such tags did not alter the chemical and biological properties of the catalyst. Halogenation permits application of a range of techniques to study the ligands of metal complexes in biological environments, including: ^19^F NMR or MRI for fluorinated compounds,^[23]^ ICP‐MS and XRF using bromine or iodine tags,[^9b^, ^24^] or PET/SPECT imaging after radiolabelling with ^131^I.^[25]^ We probed the structure and spatial localisation of a brominated transfer hydrogenation catalyst inside cells with ICP‐MS and nanoscale synchrotron XRF mapping, combined with cellular uptake and mechanistic studies. These experiments showed that the catalyst was degraded in cancer cells, probably through transport into acidic lysosomes following by reaction with cellular thiols. The chelated Br‐ligand (or a Br‐fragment), but not Os, is translocated into the nucleus. Such reactions help to explain the low intracellular TON estimated for these catalysts. This work demonstrates the utility of halogen tags as probes for MS and X‐ray based techniques which elucidate reactions of organometallic anticancer catalysts in cells. The next step for improving the efficiency of the catalysts might be to tether the chelated ligand to the arene ring, a strategy which has been used effectively in chemical systems.^[26]^ On the other hand, such ligand release reactions might be used to deliver drugs which would otherwise be poorly taken up into cells and be inactive if administered separately.

Data Availability: The data that support the findings in this study are available in the Warwick Research Archive Portal (WRAP) repository, http://wrap.warwick.ac.uk/147754/.

## Conflict of interest

The authors declare no conflict of interest.

## Supporting information

As a service to our authors and readers, this journal provides supporting information supplied by the authors. Such materials are peer reviewed and may be re‐organized for online delivery, but are not copy‐edited or typeset. Technical support issues arising from supporting information (other than missing files) should be addressed to the authors.

Supplementary
