# Supplementary material for: Tracking Reactions of Asymmetric Organo‐Osmium Transfer Hydrogenation Catalysts in Cancer Cells
Source: Angew Chem Int Ed Engl. 2021 Feb 15;60(12):6462–72. doi: 10.1002/anie.202016456 (PMC7985874; doi:10.1002/anie.202016456)
Supplement: Supplementary file 1 — Supplementary [file ANIE-60-6462-s001.pdf]

Angewandte Chemie
Eine Zeitschrift der Gesellschaft Deutscher Chemiker

Supporting Information

Tracking Reactions of Asymmetric Organo-Osmium Transfer Hydrogenation Catalysts in Cancer Cells

Elizabeth M. Bolitho, James P. C. Coverdale, Hannah E. Bridgewater, Guy J. Clarkson, Paul D. Quinn, Carlos Sanchez-Cano,* and Peter J. Sadler**

anie_202016456_sm_miscellaneous_information.pdf

Supporting Information

©Wiley-VCH 2016 69451 Weinheim, Germany

Contents

ES1 Materials **S4**
Chemical reagents S4
Biological Reagents S4
Human cell lines S4
ES2 Instrumentation **S5**
Microwave reactor S5
Nuclear Magnetic Resonance (NMR) Spectroscopy S5
High Resolution Mass Spectrometry (HR-MS) S5
CHN Elemental Analysis S5
UV-Vis Spectroscopy. S5
X-ray Crystal Structures S5
Gas Chromatography (GC-FID) S5
Inductivelycoupled Plasma-Optical Emission Spectroscopy (ICP-OES) S5
Inductively-coupledPlasma-Mass Spectrometry (ICP-MS) S5
Multiplate reader S6
Flow Cytometer S6
Plunge-freezer S6
Freeze-dryer S6
ES3 Chemical synthesis and characterisation **S7**
Synthesis of Ph-DPEN (phenyldiphenylethylenediamine) ligands S7
Synthesis of $[Os(\eta^6\text{-}p\text{-}cym)Cl_2]_2$ *precursor dimer* S11
Synthesis of $[Os^{II}(p\text{-}cym)(R_{1/2}Ph\text{-}DPEN)]$ *complexes* S11
ES4 X-ray crystal structures **S16**
ES5 Lipophilicity (Log P) studies **S18**
ES6 Density Functional Theory (DFT) **S19**
ES7 Asymmetric Transfer Hydrogenation (ATH) **S23**
^{1}H NMR Asymmetric Transfer Hydrogenation (ATH) S23
GC-FID Asymmetric Transfer Hydrogenation (ATH) S23
ES8 In vitro antiproliferative screening **S24**
Culture medium S24
Defrosting cells S24
General cell maintenance S24
Sulforhodamine B (SRB) Assay S24
Formate and acetate-dependent cell viability S25
ES9 Membrane integrity **S27**
ES10 Cell cycle analysis **S28**
ES11 ^{189}Os ICP-MS cellular accumulation studies **S28**
ICP-OES of drug stock solutions S28
General cellular accumulation protocol S28
Nitric acid ICP-MS digestion S29
ES12 ^{189}Os and ^{79}Os ICP-MS cellular accumulation studies **S31**
Optimisation of ICP-MS alkaline method S31
Alkaline ICP-MS digestion S32

ES13 Zebrafish embryo toxicity studies **S38**
ES14 Synchrotron-XRF **S39**
Preparation of Tris-glucose buffer. S39
Preparation of A549 cells on Si_3N_4 membranes. S39
XRF elemental map acquisition at I14 Beamline. S39
PyMca data analysis. S39
ImageJ data analysis S39
XRF Elemental Maps S40
Cell area and roundness factors (RF) S50
XRF quantification of Os and Br S53
Elemental colocalization of Os and Br S54
XRF lysosomal structures S55
ES15 Reactions with L-cysteine **S61**
ES16 L-BSO GSH depletion assay **S67**
ES17 Chloroquine-dependent *in vitro* activity **S68**
Chloroquine-dependent antiproliferative activity S68
Chloroquine-dependent membrane integrity S68
Chloroquine-dependent Os and Br cellular accumulation S69
Chloroquine-dependent formate cell viability S70
ES18 References **S71**

ES1 Materials

Chemical reagents. Osmium trichloride hydrate ($OsCl_3.3H_2O$) was purchased from Heraeus. α-phellandrene (>85%, CAS#99-83-2, 100G), (1*S*,2*S*)-(-)-1,2-diphenylethylenediamine (97%, CAS# 29841-69-8), (1*R*,2*R*)-(+)-1,2-diphenylethylenediamine (97%, 35132-20-8), 4-(bromo)benzenesulfonyl chloride (98%, CAS# 98-58-8, 25 g), triethylamine (>99.5%, CAS# 121-44-8), deuterated d_6-benzene (99.6 atom %, CAS# 1076-43-3), acetophenone (98%, CAS# 98-86-2), 5:2 formic acid trimethylamine azeotrope (>99%, CAS# 15077-13-1), sodium formate (CAS# 141-53-7), thiourea (ACS reagent, >99%, CAS# 62-56-6), L-ascorbic acid (BioXtra, >99%, CAS# 50-81-7), sodium chloride (99.9999%, trace metal basis, CAS# 7647-14-5), sodium hydrogen carbonate, L-cysteine, tris(hydroymethyl)aminomethane (CAS# 77-86-1), ammonium acetate anhydrous and magnesium sulfate were purchased from Sigma Aldrich, UK. Sodium acetate anhydrous (CAS# 127-09-3) and potassium hydroxide pellets (Extra Pure, CAS# 1310-58-3) were purchased from Fischer Scientific. Osmium (1000 ± 10 μg/mL hexchlorodiammonium osmate in 15% *v*/*v* hydrochloric acid, platinum (1001 ± 12 mg/L TraceCERT® platinum in 5% *v*/*v* hydrochloric acid), bromine (1000 ± 5 μg/mL potassium bromide in water) and ruthenium (995 ± 4 μg/mL ruthenium chloride in 10% *v*/*v* hydrochloric acid) for ICP trace analysis were purchased from Inorganic Ventures and stored at 276 K. All commercial solvents were purchased from Sigma Aldrich and used as specified by the manufacturer (unless stated otherwise).

Biological reagents. Dulbecco's Modified Eagles Medium (DMEM), phosphate buffer saline (PBS), penicillin / streptomycin, L-glutamine, heat-inactivated fetal calf serum (FCS) and trypsin / EDTA was prepared by staff at the School of Life Sciences (University of Warwick) upon purchasing from PAA Laboratories. Methyl-β-cyclodextrin (BioReagent, CAS# 128446-36-6, 1 g), verapamil hydrochloride (CAS# 152-11-4), poly-L-lysine solution (0.01%, sterile-filtered, BioReagent, CAS# 25988-63-0) and chloroquine diphosphate salt (BioReagent, CAS#-50-63-5, >98.5%, 10 g) were purchased from Sigma Aldrich, UK. FractionPREP Cell Fractionation Kit was purchased from Cambridge Bioscience Ltd.

Human cell lines. A549 human lung carcinoma (86012804), A2780 human ovarian carcinoma (93112519), MCF7 human breast adenocarcinoma (86012803), PC3 human prostate carcinoma cells (90112714) and MRC5 human fetal fibroblasts (84101801) were purchased from the European Collection of Cell Cultures (ECACC). Every 6 months, mycoplasma-free status was confirmed.

ES2 Instrumentation

Microwave reactor. The synthesis of the precursor dimer $[Os(p\text{-cymene})Cl_2]_2$ was performed using a CEM Discovery-SP microwave reactor. Microwave conditions: temperature; 413 K, voltage; 150 W and pressure; 250 *psi*.

Nuclear Magnetic Resonance Spectroscopy (NMR). Characterisation of structures by ^1H NMR and ^{13}C NMR were performed on a Bruker Avance III HD-500 instrument (University of Warwick). ^1H NMR transfer hydrogenation kinetic experiments were performed on Bruker Avance III AV-400 instrument (University of Warwick). All measurements were performed at 298 K, unless otherwise specified and spectra acquired using standard pulse sequences referenced to the specified residual solvents.

High Resolution Mass Spectrometry (HR-MS). High resolution mass spectra were recorded on a Bruker UHR-Q-TOF MaXis plus in the positive scan range m/z 50-2400. All samples were prepared in acetonitrile, unless specified otherwise. Direct infusion using a syringe pump (2 µL/min) was used and calibrated to sodium formate. Source conditions: Positive ESI, -500 V end-plate, 0.4 bar N_2 nebulizer gas pressure, 4 L/min dry gas, 453 K dry temperature, 200 Vpp funnel RF, 200 Vpp multiple RF, m/z = 55 quadruple minimum mass, 5.0 eV collision energy, 600 Vpp collision RF, 50-200 Cpp ramping of ion cooler, 121 µs transfer time, 1 µs pre-pulse storage time.

CHN elemental analysis. C, H and N elemental analysis was measured on an Exeter elemental analyser CE440 by Warwick Analytical Services UK.

UV-vis Spectroscopy. UV-vis absorption measurements were performed on a Varian Cary 300 Bio Scan spectrometer using 700 µL quartz cuvettes. Spectrometer settings: pathlength = 1 cm, analysis range = 800-200 nm, scan rate 600 nm / min, average acquisition time = 0.1 s, data interval 1 nm. Date were processed using Winlab UV software.

X-ray crystal structures. A single crystals were mounted on a glad fibre with oil and analysed on a Oxford Diffraction Gemini diffractometer with a ruby charge-coupled detector at 150 K.

Gas Chromatography (GC-FID). Chiral GC measurements were carried out on a Hewlett-Packard 5890 instrument linked to PC running DataApex Clarity software

Inductively-coupled plasma-optical emission spectroscopy (ICP-OES). Solutions of complexes in 5% *v*/*v* DMSO and 95% *v*/*v* DMEM were analysed using PerkinElmer 5300DV ICP-OES. Calibration standards of analytes of interest were prepared in the range 50-700 ppb from a 10,000 ppm certified reference materials (Inorganic Ventures) in 3.6% *v*/*v* nitric acid (10 mM thiourea, 100 mg/L L-ascorbic acid). The salinity of the calibration standards was adjusted to the sample matrix of culture media using NaCl. Samples were diluted appropriately to fit the calibration range, so that dissolved solids did not exceed 0.1% *w*/*v*. Samples were analysed on a Perkin Elmer Optima 5300 DV Optical Emission Spectrophotometer: Os (λ=225.585; 228.226 nm). Data were processed in WinLab32 Offline 3.4.1 software for Windows.

Inductively coupled plasma-mass spectrometry (ICP-MS). ICP-MS analysis for cellular accumulation experiments were analysed on an Agilent 7900 series ICP-MS spectrometer in no-gas mode. Calibrations of the elements of interest (^{189}Os, ^{79}Br) were prepared in the range 0.1-1000 ppb from 10'000 ppm certified reference materials (Inorganic Ventures) using either 3.6% *v*/*v* nitric acid (380.6 mg thiourea, 50 mg L-ascorbic acid in 500 mL) or 1% *m*/*v* TMAH, as specified. Dissolved solids did not exceed 0.1% *w*/*v*. For TMAH studies of ^{189}Os and ^{79}Br,

^{101}Ru (50 ppb) was used as an internal standard. For all other ICP-MS experiments, ^{166}Er (10 ppb) was used. Data processing was carried out using ChemStation Agilent Technologies in Offline Data Analysis mode.

(i) ***HNO_3 digestion:*** Cell pellets were digested using freshly-distilled 72% *v*/*v* nitric acid (200 µL) at 353 K for 12 h. The samples were then diluted 20-fold using stabilised milliQ water (containing 10 mM thiourea and 100 mg/L L-ascorbic acid) to achieve a final concentration of 3.6% *v*/*v* nitric acid (total dissolved solids <0.2% *w*/*v*). Data acquisition was performed using an internal standard of erbium (^{166}Er=10 ppb) in no gas mode.

(ii) ***TMAH digestion:*** Cell pellets were digested using an aqueous solution of 25% *m*/*v* TMAH (500 µL) at 353 K overnight, adapted from methods published in the literature.11, 12 The samples were then diluted 25-fold to achieve 1% *m*/*v* TMAH (total dissolved solids <0.1% *w*/*v*) using milliQ water. Data acquisition was performed using an internal standard of ruthenium (^{101}Ru=50 ppb). Calibration standards were prepared as previously described.

Multiplate reader. 96-well plates for all SRB assays were analysed using BioRad iMark microplate reader using the λ = 492 nm filter.

Flow cytometer. Cell samples for membrane integrity or cell cycle analysis were analysed on the BD LSRII flow cytometer with BD FACSDIVA V8.01 for Windows and processed using FlowJo V10 for Windows.

Plunge-freezer. Silicon nitride (Si_3N_4) membranes were plunge-frozen 30% propane in liquid ethane (cooled on liquid nitrogen) using an in-house manufactured manual plunge-freezer at the School of Life Science (University of Warwick).

Freeze-dryer. Silicon nitride (Si_3N_4) membranes were freeze-dried using Alpha 2-4 LDplus Christ model, operating at 188 K temperature and 0.0024 mbar vacuum.

ES3 Chemical synthesis and characterisation

Synthesis of Phenylsulfonyl diphenylethylenediamine (Ph-DPEN) ligands

Phenylsulfonyl diphenylethylenediamine (Ph-DPEN) ligands were synthesised based on a literature method (Scheme 1).[1]

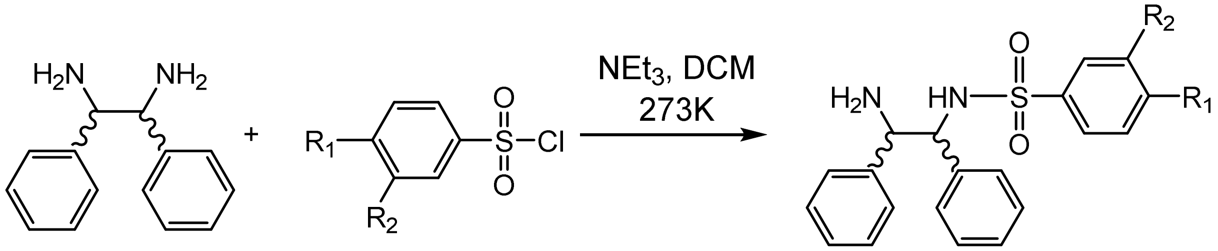

Scheme 1. General synthetic scheme for the synthesis of Ph-DPEN (phenyldiphenylethylene diamine) ligands using varying phenylsulfonyl chlorides and *SS* or *RR*-DPEN in the presence of base (triethylamine).

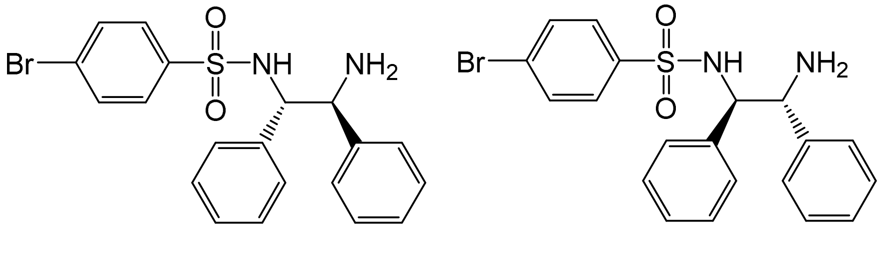

***SS*-L1** ***RR*-L1**

(1*S*, 2*S*)-*p*-BrPh-DPEN *SS*-L1. To a solution of (*1S*, *2S*)-diphenylethylenediamine (1.28 g, 6.03 mmol) and triethylamine (0.8 mL, 5.77 mmol) in anhydrous DCM (15 mL) cooled in an ice bath was added dropwise 4-bromobenzenesulfonyl chloride (1.84 g, 7.2 mmol) in anhydrous DCM (10 mL). After the ice bath was removed, the reaction mixture was stirred for 2 h. Distilled water (30 mL) was added to the reaction mixture. The organic phase was separated and the aqueous phase extracted with DCM (3 × 10 mL), combined with the organic phase and dried over magnesium sulphate. A pale yellow solution was obtained and concentrated *in vacuo*. Diethyl ether (10mL) was added to precipitate a white solid (1.12 g, 2.60 mmol, 43%). ^{1}H NMR (500 MHz, d-DMSO, 25 °C, TMS): δ = 7.43 (d, ^{3}J(H,H) = 8.5 Hz, 2H, ArH), 7.31 (d, ^{3}J(H,H) = 8.5 Hz, 2H, ArH), 7.12-7.0 (m, 5H, ArH), 7.00 (d, ^{3}J(H,H) = 6.6 Hz, 3H, ArH), 6.07-6.92 (m, 2H, ArH), 4.33 (d, ^{3}J(H,H) = 7.2 Hz, 1H, C*H*NHTs), 3.97 (d, ^{3}J(H,H) = 7.2 Hz, 1H, C*H*NH). ^{13}C NMR (125 MHz, DMSO): δ = 142.8, 140.9, 140.0, 132.0, 128.6, 128.1, 127.9, 127.8, 127.7, 127.1, 126.9, 125.9, 65.3, 61.0, 40.6, 40.4, 40.2. HRMS (ESI): *m/z* calculated for $C_{20}H_{20}BrN_2O_2S$ [M+H$^+$]: 431.0423; found: 431.0427. Elemental analysis: found (calculated for $C_{20}H_{19}BrN_2O_2S$): C 55.10 (55.69), H 4.31 (4.44), N 6.40 (6.49).

(1*R*, 2*R*)-*p*-BrPh-DPEN *RR*-L1. To a solution of (1*R*, 2*R*)-diphenylethylenediamine (1.25 g, 5.89 mmol) and triethylamine (0.8 mL, 5.77 mmol) in anhydrous DCM (15 mL) cooled in an ice bath was added dropwise 4-bromobenzenesulfonyl chloride (1.87 g, 7.32 mmol) in anhydrous DCM (10 mL). After the ice bath was removed, the reaction mixture was stirred for two hours. Distilled water (30 mL) was added to the reaction mixture. The organic phase was separated and the aqueous phase extracted with DCM (3 × 10 mL), combined with the organic phase and dried over magnesium sulphate. A pale yellow solution was obtained and concentrated *in vacuo*. Diethyl ether (10mL) was added to precipitate a white solid (1.12 g,

2.60 mmol, 43%). ^{1}H NMR (500 MHz, d-DMSO, 25 °C, TMS): δ = 7.43 (d, ^{3}J(H,H) = 8.5 Hz, 2H, ArH), 7.31 (d, ^{3}J(H,H) = 8.5 Hz, 2H, ArH), 7.14-7.05 (m, 5H, ArH), 7.00 (d, ^{3}J(H,H) = 6.7 Hz, 3H, ArH), 6.96-6.92 (m, 2H, ArH), 4.33 (d, ^{3}J(H,H) = 7.2 Hz, 1H, CHNHTs), 3.97 (d, ^{3}J(H,H) = 7.2 Hz, 1H, CHNH). ^{13}C NMR (125 MHz, DMSO): δ = 142.8, 140.9, 140.0, 132.0, 128.6, 128.1, 127.9, 127.8 127.7, 127.1, 126.9, 125.9, 65.3, 61.0, 46.2, 40.6, 40.4, 40.2. HRMS (ESI): *m/z* calculated for $C_{20}H_{20}BrN_2O_2S$ [M+H^{+}]: 431.0423; found: 431.0423. Elemental analysis: found (calculated for $C_{20}H_{19}BrN_2O_2S$): C 55.02 (55.69), H 4.34 (4.44), N 6.29 (6.49).

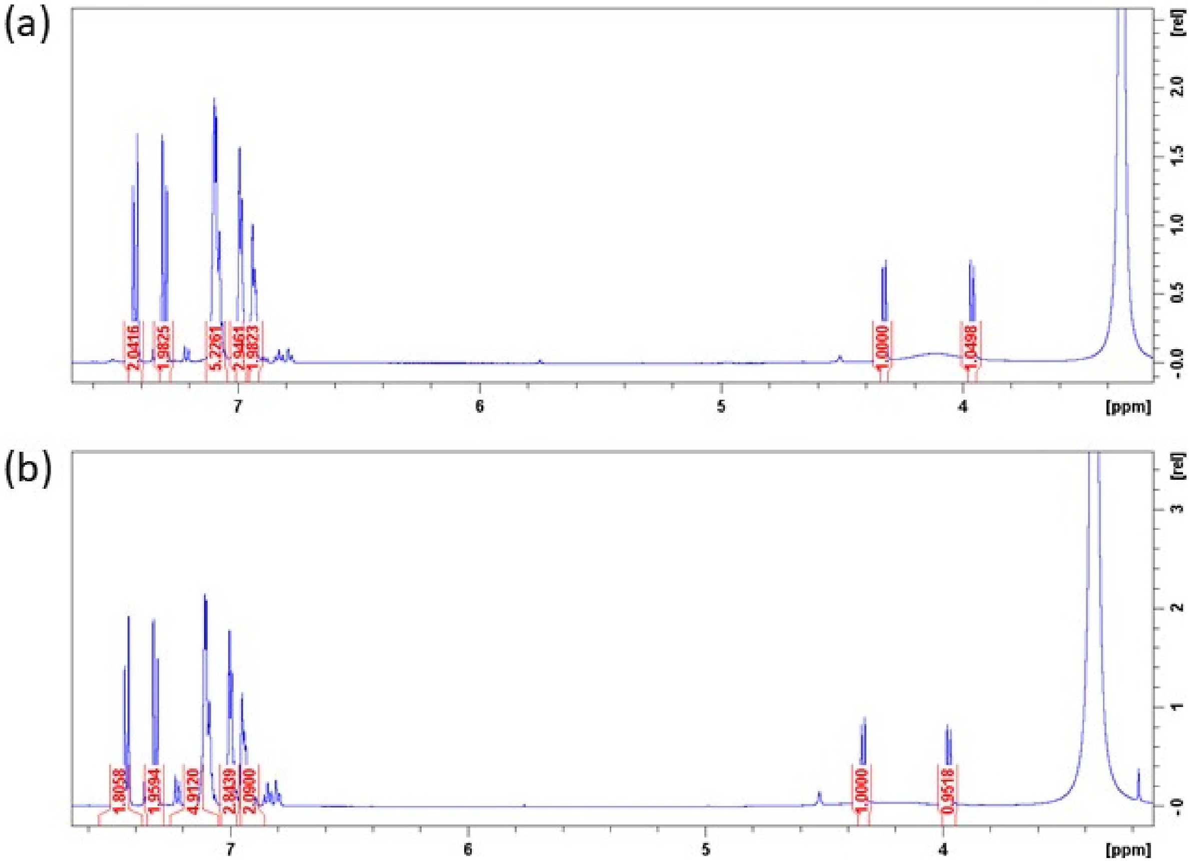

Figure S1. 500 MHz ^{1}H NMR spectra of (a) ***SS*-L1** and (b) ***RR*-L1** in d_6-DMSO.

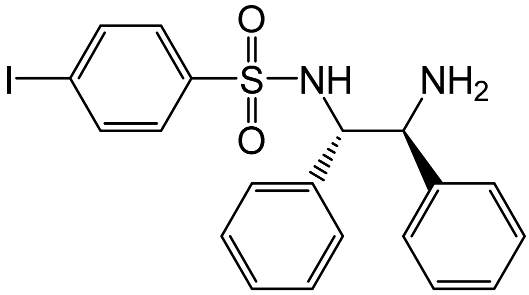

***SS*-L2**

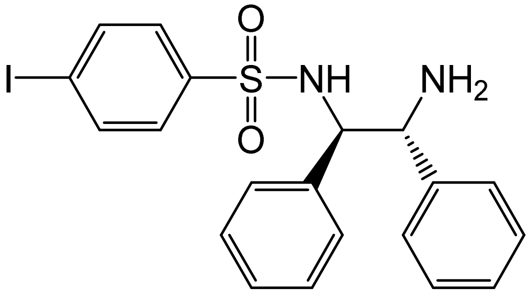

***RR*-L2**

(1*S*, 2*S*)-*p*-IPh-DPEN (*SS*-L2). To a solution of (1*S*, 2*S*)-diphenylethylenediamine (1.27 g, 5.98 mmol) and triethylamine (0.83 mL, 5.77 mmol) in anhydrous DCM (15 mL) cooled in an ice bath was added dropwise 4-iodobenzenesulfonyl chloride (2.19 g, 7.20 mmol) in anhydrous DCM (10 mL). After the ice bath was removed, the reaction mixture was stirred for two hours. Distilled water (30 mL) was added to the reaction mixture. The organic phase was separated and the aqueous phase extracted with DCM (3 × 10 mL), combined with the organic phase and

dried over magnesium sulphate. A pale yellow solution was obtained and concentrated *in vacuo*. Diethyl ether (10mL) was added to precipitate a white solid (0.62 g, 1.30 mmol, 22%).^{1}H NMR (500 MHz, d_6-DMSO, 298 K, TMS): δ=7.60 (d, ^{3}J(H,H)=8.4 Hz, 2H, ArH), 7.15 (d, ^{3}J(H,H)=8.4 Hz, 2H, ArH), 7.13-6.78 (m, 10H, ArH), 4.32 (d, ^{3}J(H,H)=7.3 Hz, 1H, C*H*NHTs), 3.97 (d, ^{3}J(H,H)=7.3 Hz, 1H, C*H*NH). ^{13}C NMR (125 MHz, DMSO): δ=142.7, 141.2, 140.0, 137.8, 128.3, 128.1, 128.0, 127.8, 127.7, 127.1, 126.9, 100.0, 65.3, 61.0, HRMS (ESI): *m/z* calculated for $C_{20}H_{20}N_2IO_2S$ [M+H^{+}]: 479.0290; found: 479.0289. Elemental analysis: found (calculated for $C_{20}H_{19}N_2IO_2S$): C 49.72 (50.22), H 3.92 (4.00), N 5.75 (5.86).

(1*R*, 2*R*)-*p*-IPh-DPEN (*RR*-L2). To a solution of (1*R*, 2*R*)-diphenylethylenediamine (1.03 g, 4.90 mmol) and triethylamine (0.67 mL, 4.83 mmol) in anhydrous DCM (15 mL) cooled in an ice bath was added dropwise 4-iodobenzenesulfonyl chloride (1.73 g, 5.70 mmol) in anhydrous DCM (10 mL). After the ice bath was removed, the reaction mixture was stirred for two hours. Distilled water (30 mL) was added to the reaction mixture. The organic phase was separated and the aqueous phase extracted with DCM (3 × 10 mL), combined with the organic phase and dried over magnesium sulphate. A pale yellow solution was obtained and concentrated *in vacuo*. Diethyl ether (10mL) was added to crash out a white solid (0.75 g, 1.59 mmol, 27%). ^{1}H NMR (500 MHz, d_6-DMSO, 298 K, TMS): δ=7.60 (d, ^{3}J(H,H)=8.3 Hz, 2H, ArH), 7.15 (d, ^{3}J(H,H)=8.3 Hz, 2H, ArH), 7.13-6.90 (m, 10H, ArH), 4.38 (d, ^{3}J(H,H)=7.4 Hz, 1H, C*H*NHTs), 3.99 (d, ^{3}J(H,H)=7.4 Hz, 1H, C*H*NH). ^{13}C NMR (125 MHz, d_6-DMSO): δ=141.2, 140.1, 139.8, 137.8, 128.3, 128.1, 128.0, 127.8, 127.7, 127.2, 126.9, 100.1, 65.1, 60.9. HRMS (ESI): *m/z* calculated for $C_{20}H_{20}IN_2O_2S$ [M+H^{+}]: 479.0290; found: 479.0288. Elemental analysis: found (calculated for $C_{20}H_{20}N_2IO_2S$): C 43.50 (50.22), H 4.25 (4.00), N 5.04 (5.86).

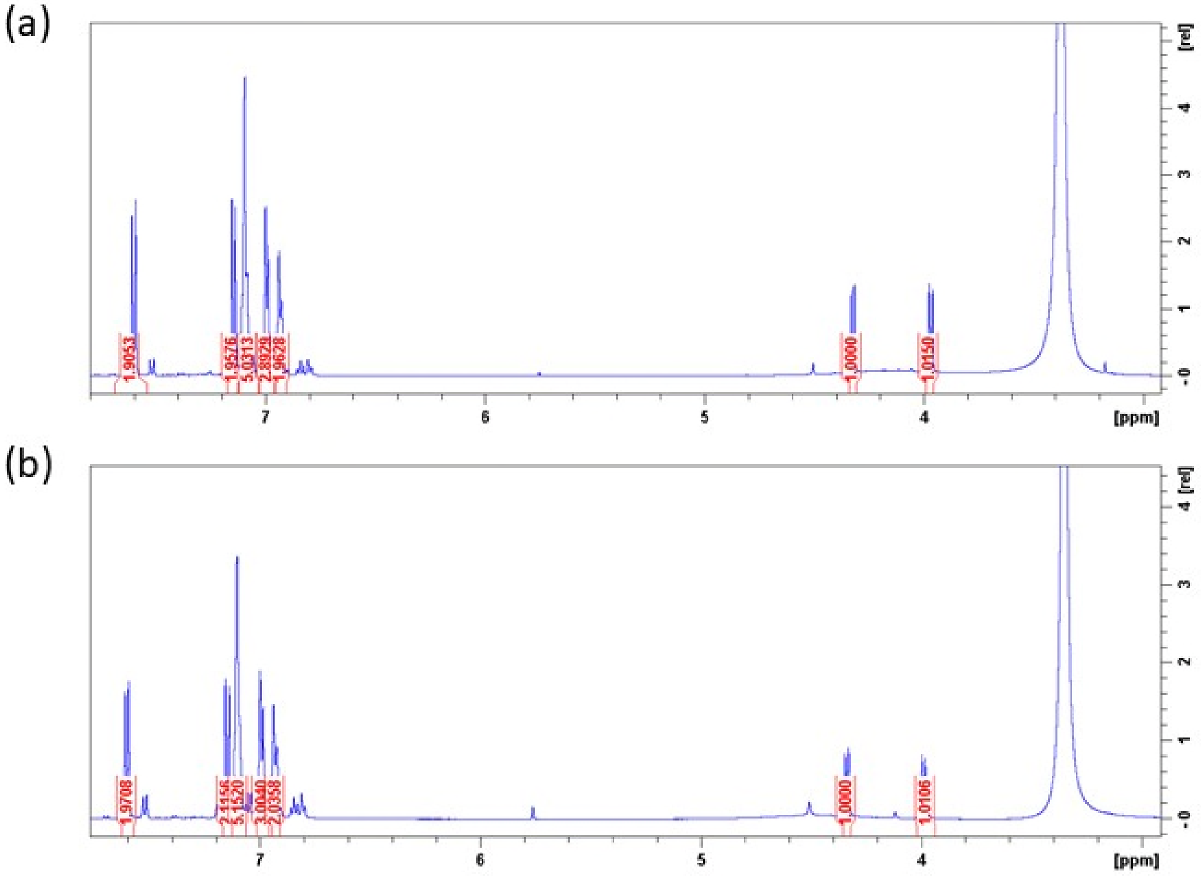

Figure S2. 500 MHz ^{1}H NMR spectra of (a) ***SS*-L2** and (b) ***RR*-L2** in d_6-DMSO.

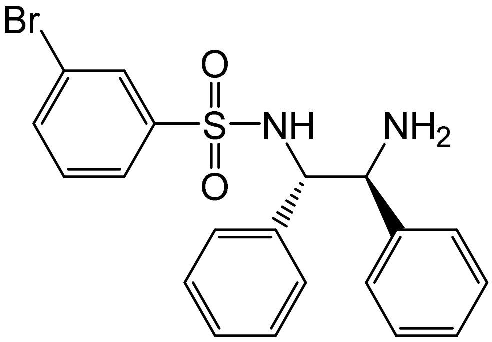

***SS*-L3**

(1*S*, 2*S*)-*m*-BrPh-DPEN (*SS*-L3). To a solution of (1*S*, 2*S*)-diphenylethylenediamine (0.36 g, 1.84 mmol) and triethylamine (0.25 mL, 1.80 mmol) in anhydrous DCM (15 mL) cooled in an ice bath was added dropwise 3-bromobenzenesulfonyl chloride (0.32 mL, 2.25 mmol) in anhydrous DCM (10 mL). After the ice bath was removed, the reaction mixture was stirred for two hours. Distilled water (30 mL) was added to the reaction mixture. The organic phase was separated and the aqueous phase extracted with DCM (3 × 10 mL), combined with the organic phase and dried over magnesium sulphate. A pale yellow solution was obtained and concentrated *in vacuo*. Diethyl ether (10mL) was added to crash out a white solid (0.35 g, 0.91 mmol, 50%). ^{1}H NMR (400 MHz, d_6-DMSO, 298 K, TMS): δ=8.78 (s, 2H, NH), 7.55 (d, ^{3}J(H,H)=8.0 Hz, 1H, ArH), 7.50 (d, ^{3}J(H,H)=7.9 Hz, 2H, ArH), 7.37 (d, ^{3}J(H,H)=7.8 Hz, 2H, ArH), 7.15 (t, ^{3}J(H,H)=7.7 Hz, 6H, ArH), 6.83 (m, 3H, ArH), 4.47 (d, ^{3}J(H,H)=8.2 Hz, 1H, C*H*NHTs), 4.15 (d, ^{3}J(H,H)=8.6 Hz, 1H C*H*NH). ^{13}C NMR (125 MHz, d_6-DMSO): δ=142.0, 141.8, 140.0, 139.7, 139.6, 137.8, 128.3, 128.1, 128.0, 127.8, 127.7, 127.1, 126.9, 100.0, 65.3, 61.0. HRMS (ESI): *m/z* calculated for $C_{20}H_{20}BrN_2O_2S$ [M+H$^+$]: 431.0429; found: 431.0425. Elemental analysis: found ($C_{20}H_{19}BrN_2O_2S$): C 54.96 (55.69), H 4.62 (4.44), N 6.49 (6.49).

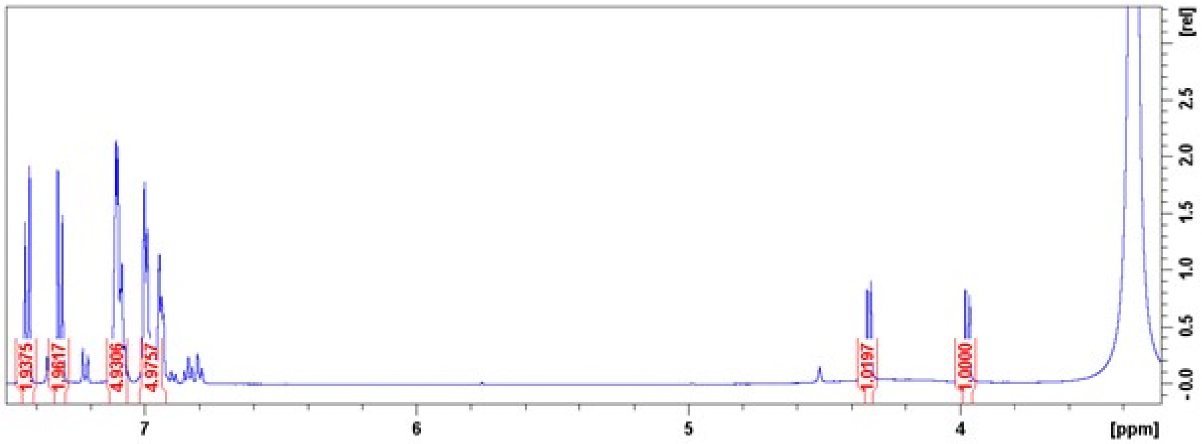

Figure S3. 500 MHz ^{1}H NMR spectrum of ***SS*-L3** in d_6-DMSO.

Synthesis of [Os(η⁶-p-cym)Cl₂]₂ precursor dimer

[Os(η⁶-*p*-cymene)Cl₂]₂ dimer was synthesised using a literature method (Scheme 2).[2] Os[(*p*-cym)(Ph-DPEN)] complexes (**2**, **3** and **5**) were synthesised based on a literature method (Scheme 3).[3]

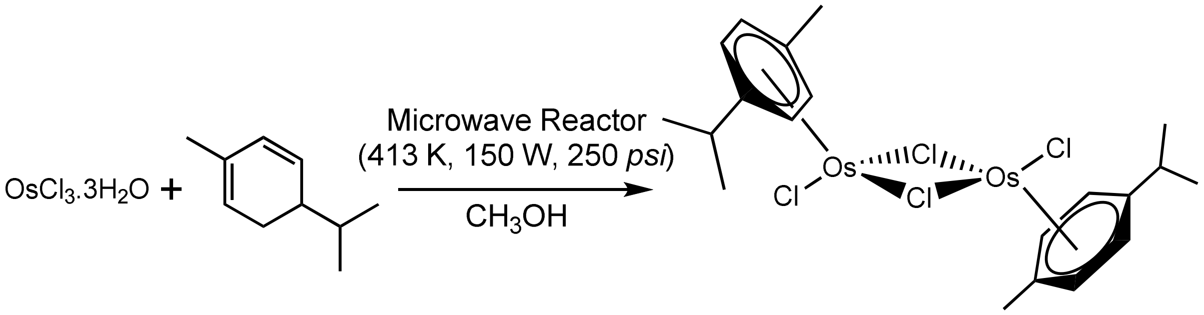

Scheme 2. Synthetic scheme for [Os(η⁶-*p*-cym)Cl₂]₂ dimer, used as precursor dimer for the synthesis of [Os(*p*-cym)(Ph-DPEN)] complexes using an optimized microwave method (413 K, 150 W, 250 *psi*).[2]

Synthesis of [Os^{II}(p-cym)(R$_{1/2}$Ph-DPEN)] complexes

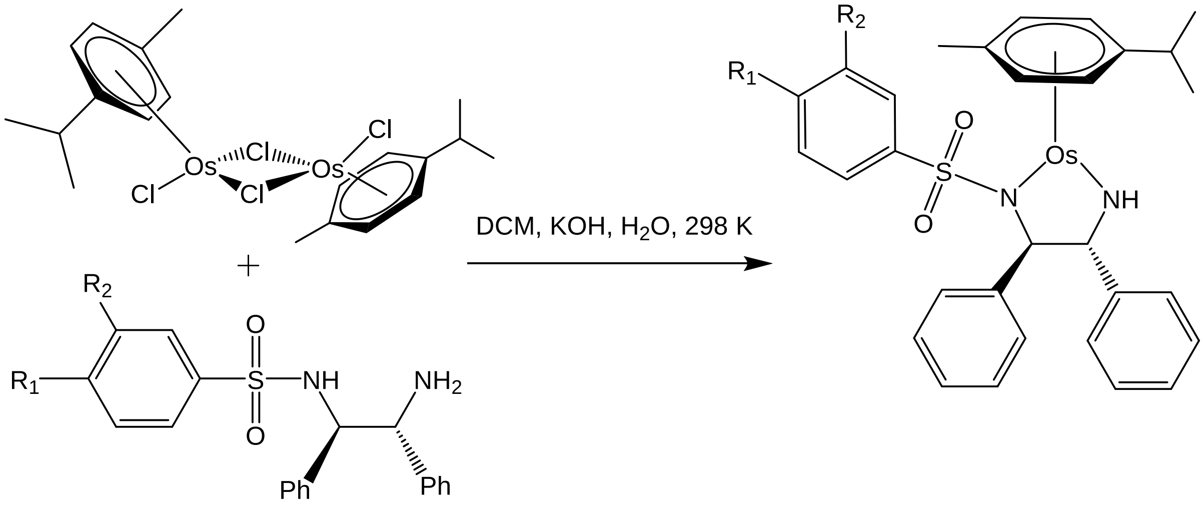

Scheme 3. General synthetic scheme for [Os^{II}(*p*-cym)(R$_{1/2}$Ph-DPEN)] complexes (**2**-**5**): where *p*-cym=*para*-cymene, RPh-TsDPEN=R-phenyldiphenylethylenediamine with substituent R=Br, I.

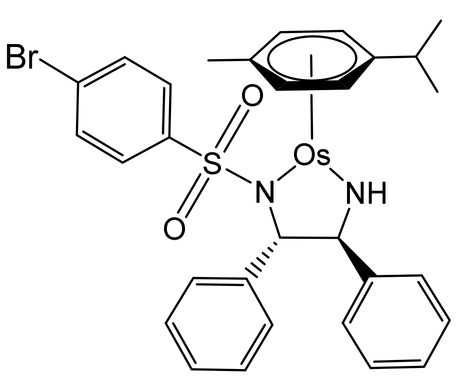

***SS*-2**

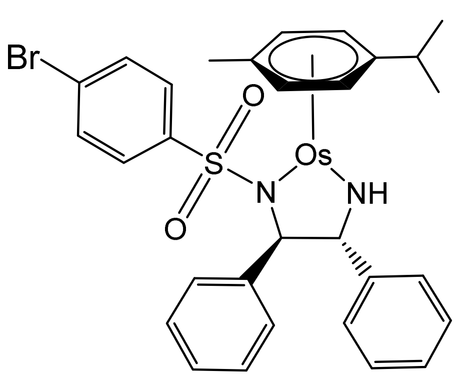

***RR*-2**

Synthesis of *SS*-2. To a stirred solution of osmium *p*-cymene chloride dimer (50 mg, 0.06 mmol) in DCM (5 mL) was added (*1S, 2S*)-*p*-BrPh-DPEN (60 mg, 0.14 mmol). A bright yellow solution was formed. KOH (50 mg) was added, followed by distilled water (30 mL) and brick-red organic layer was formed. The organic layer was extracted with DCM (3 × 10 mL) and the resulting red solution was concentrated *in vacuo*. A dark orange solid was obtained (52 mg, 0.07 mmol, 58%). ^{1}H NMR (500 MHz, d_6-DMSO, 298 K, TMS): δ = 8.21 (d, ^{3}J(H,H) = 3.7 Hz, 1H, NH), 7.46 (d, ^{3}J(H,H) = 7.5 Hz, 2H, ArH), 7.31 (d, ^{3}J(H,H) = 8.4 Hz, 2H, ArH), 7.27-7.09 (m, 11H, ArH), 5.95 (d, ^{3}J(H,H) = 5.6 Hz, 1H, Os-ArH), 5.83 (d, ^{3}J(H,H) = 5.5 Hz, 1H, Os-ArH), 5.71 (d, ^{3}J(H,H) = 5.4 Hz, 1H, Os-ArH), 5.64 (d, ^{3}J(H,H) = 5.4 Hz, 1H, Os-ArH), 4.20 (s, 1H, TsNCH), 3.85 (d, ^{3}J(H,H) = 4.3 Hz, 1H, CH), 2.27 (s, 3H CH_3), 1.28 (d, ^{3}J(H,H) = 6.8 Hz, 3H, $CH(CH_3)_2$), 1.18 (d, ^{3}J(H,H) = 6.9 Hz, 3H, $CH(CH_3)_2$). ^{13}C NMR (125 MHz, DMSO): δ = 146.8, 144.9, 143.9, 131.3, 128.5, 128.2, 127.9, 127.1, 126.7, 126.5, 126.9, 91.1, 82.7, 80.2, 76.8, 73.8, 71.4, 67.9, 40.6, 40.2, 40.1, 32.6, 23.7, 23.5, 20.8. UV/Vis λ_{max} 259, 412, 477 nm; HRMS (ESI): *m/z* calculated for $C_{30}H_{32}BrN_2O_2OsS$ [M+H$^+$]: 755.0977; found: 755.0946. Elemental analysis: found (calculated for $C_{30}H_{31}BrN_2O_2OsS$): C 47.14 (47.80), H 4.10 (4.15), N 3.51 (3.72).

Synthesis *RR*-2. To a stirred solution of osmium *p*-cymene chloride dimer (49 mg, 0.06 mmol) in DCM (5 mL) was added (*1R, 2R*)-*p*-BrPh-DPEN (60 mg, 0.14 mmol). A bright yellow solution was formed. KOH (50 mg) was added, followed by distilled water (30 mL) and brick-red organic layer was formed. The organic layer was extracted with DCM (3 x 10 mL) and the resulting red solution was concentrated *in vacuo*. A dark orange solid was obtained (48 mg, 0.06 mmol, 53 %). ^{1}H NMR (500 MHz, d_6-DMSO, 298 K, TMS): δ = 8.21 (d, ^{3}J(H,H) = 4.0 Hz, 1H, NH), 7.46 (d, ^{3}J(H,H) = 7.5 Hz, 2H, ArH), 7.31 (d, ^{3}J(H,H) = 8.5 Hz, 2H, ArH), 7.27-7.11 (m, 11H, ArH), 5.95 (d, ^{3}J(H,H) = 5.7 Hz, 1H, Os-ArH), 5.83 (d, ^{3}J(H,H) = 5.5 Hz, 1H, Os-ArH), 5.71 (d, ^{3}J(H,H) = 5.5 Hz, 1H, Os-ArH), 5.64 (d, ^{3}J(H,H) = 5.6 Hz, 1H, Os-ArH), 4.20 (s, 1H, TsNCH), 3.85 (d, ^{3}J(H,H) = 4.4 Hz, 1H, CH), 2.27 (s, 3H CH_3), 1.28 (d, ^{3}J(H,H) = 6.9 Hz, 3H, $CH(CH_3)_2$), 1.18 (d, ^{3}J(H,H) = 6.9 Hz, 3H, $CH(CH_3)_2$). ^{13}C NMR (125 MHz, DMSO): δ = 146.8, 144.8, 143.8, 131.3, 129.3, 128.5, 128.2, 127.9, 127.4, 127.1, 126.9, 126.6, 91.1, 82.7, 80.2, 76.8, 73.8, 71.4, 40.6, 40.4, 40.3, 32.6, 23.7. 23.5, 20.8. UV/Vis λ_{max} 259, 412, 477 nm; HRMS (ESI): *m/z* calculated for $C_{30}H_{32}BrN_2O_2OsS$ [M+H$^+$]: 755.0977; found: 755.0969. Elemental analysis: found (calculated for $C_{30}H_{31}BrN_2O_2OsS$): C 48.55 (47.80), H 4.20 (4.15), N 4.05 (3.72).

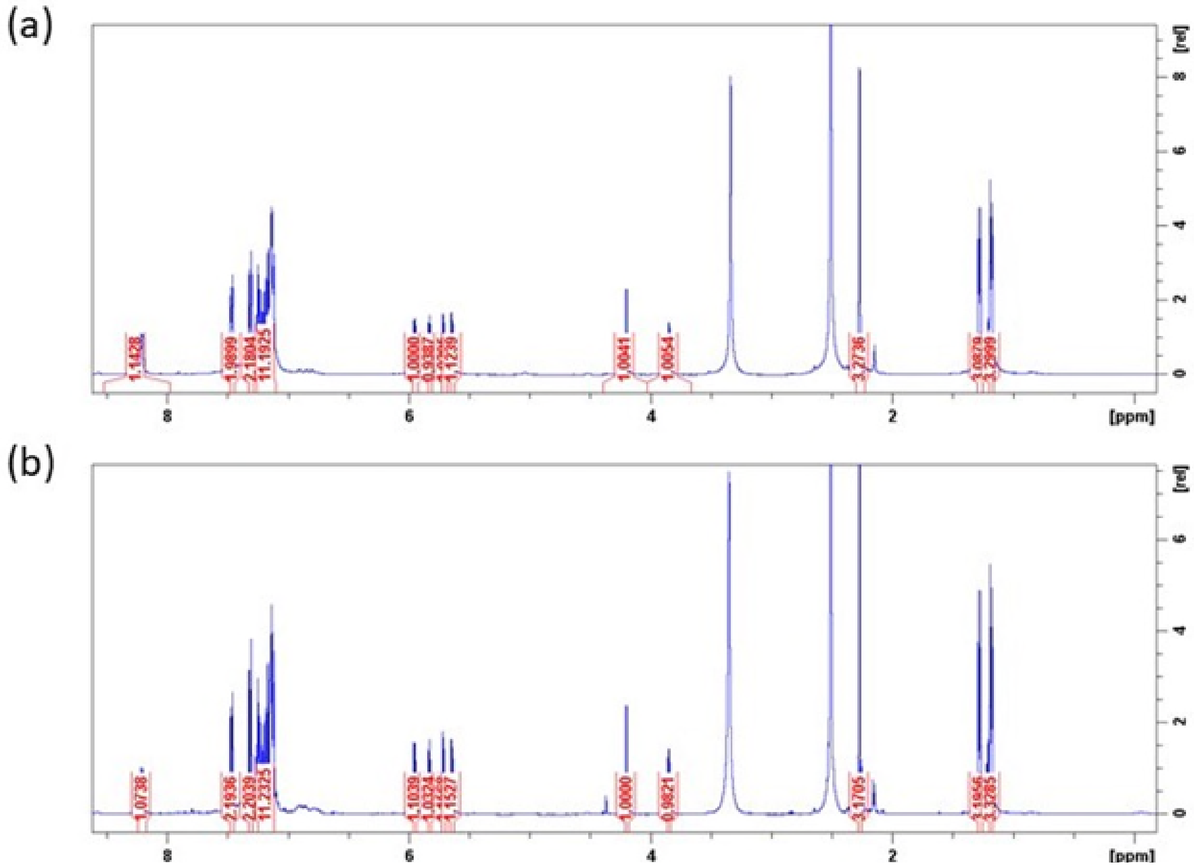

Figure S4. 500 MHz ^{1}H NMR spectra of (a) ***SS*-2** and (b) ***RR*-2** in d$_6$-DMSO.

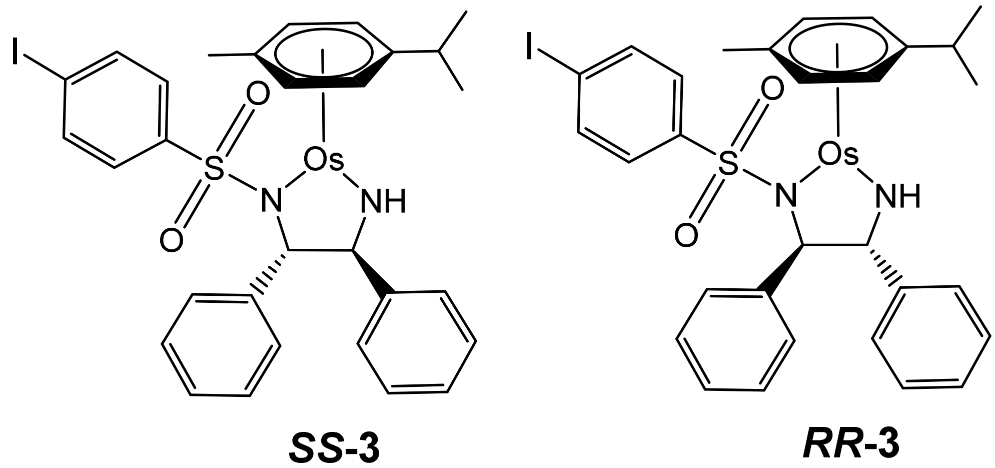

***SS*-3** ***RR*-3**

[Os(η^6-*p*-cymene)((1*S*, 2*S*)-IPh-DPEN)] (*SS*-3). To a stirred solution of osmium *p*-cymene chloride dimer (50 mg, 0.06 mmol) in DCM (5 mL) was added (*1S*, *2S*)-*p*-IPh-DPEN (***SS*-L2**, 67 mg, 0.14 mmol). A bright yellow solution was formed. Potassium hydroxide (50 mg) was added, followed by distilled water (30 mL) and brick-red organic layer was formed. The organic layer was extracted with DCM (3 x 10 mL) and the resulting red solution was concentrated *in vacuo*. A dark orange solid was obtained (47 mg, 0.06 mmol, 49%).^{1}H NMR (500 MHz, d$_6$-DMSO, 298 K, TMS): δ=8.19 (d, ^3J(H,H)=4.1 Hz, 1H, NH), 7.49 (d, ^3J(H,H)=8.4 Hz, 2H, ArH), 7.46 (d, ^3J(H,H)=7.7 Hz, 2H, ArH), 7.27-7.11 (m, 9H, ArH), 6.96 (d, ^3J(H,H)=8.3 Hz, 2H, ArH), 5.94 (d, ^3J(H,H)=5.6 Hz, 1H, Os-ArH), 5.82 (d, ^3J(H,H)=5.5 Hz, 1H, Os-ArH), 5.70 (d, ^3J(H,H)=5.5 Hz, 1H, Os-ArH), 5.62 (d, ^3J(H,H)=5.6 Hz, 1H, Os-ArH), 4.19 (s, 1H, TsNCH), 3.84 (d, ^3J(H,H)=4.4 Hz, 1H, CH), 2.27 (s, 3H CH_3), 1.27 (d,

^3J(H,H)=6.9 Hz, 3H, CH(CH_3)$_2$), 1.18 (d, ^3J(H,H) = 6.9 Hz, 3H, CH(CH_3)$_2$). ^{13}C NMR (125 MHz, DMSO): δ=146.9, 144.9, 144.2, 137.2, 128.3, 128.2, 127.9, 127.4, 127.1, 126.9, 126.6, 98.3, 91.1, 82.7, 80.2, 76.8, 73.8, 71.4, 67.2, 40.6, 40.4, 40.2, 40.1, 32.6, 31.2, 23.7, 23.5, 20.7. UV/Vis λ_{max} 272, 412, 479 nm; HRMS (ESI): *m/z* calculated for $C_{30}H_{32}IN_2O_2OsS$ [M+H$^+$]: 803.0839; found: 803.0832. Elemental analysis: found (calculated for $C_{30}H_{31}N_2IO_2OsS$): C 45.14 (45.00), H 3.84 (3.90), N 3.60 (3.50).

[Os(η^6-*p*-cymene)((1*R*, 2*R*)-IPh-DPEN)] (*RR*-3). To a stirred solution of osmium *p*-cymene chloride dimer (50 mg, 0.06 mmol) in DCM (5 mL) was added (*1R*, *2R*)-*p*I-DPEN (***RR*-L2**, 68 mg, 0.14 mmol). A bright yellow solution was formed. Potassium hydroxide (50 mg) was added, followed by distilled water (30 mL) and brick-red organic layer was formed. The organic layer was extracted with DCM (3 x 10 mL) and the resulting red solution was concentrated *in vacuo*. A dark orange solid was obtained (68 mg, 0.08 mmol, 71%).^1H NMR (500 MHz, d_6-DMSO, 298 K, TMS): δ=8.20 (d, ^3J(H,H)=4.0 Hz, 1H, NH), 7.49 (d, ^3J(H,H)=8.4 Hz, 2H, ArH), 7.46 (d, ^3J(H,H)=7.8 Hz, 2H, ArH), 7.27-7.11 (m, 9H, ArH), 6.96 (d, ^3J(H,H)=8.3 Hz, 2H, ArH), 5.94 (d, ^3J(H,H)=5.6 Hz, 1H, Os-ArH), 5.82 (d, ^3J(H,H)=5.5 Hz, 1H, Os-ArH), 5.70 (d, ^3J(H,H)=5.5 Hz, 1H, Os-ArH), 5.62 (d, ^3J(H,H)=5.6 Hz, 1H, Os-ArH), 4.19 (s, 1H, TsNCH), 3.84 (d, ^3J(H,H)=4.4 Hz, 1H, CH), 2.27 (s, 3H CH_3), 1.27 (d, ^3J(H,H)=6.9 Hz, 3H, CH(CH_3)$_2$), 1.18 (d, ^3J(H,H)=6.9 Hz, 3H, CH(CH_3)$_2$). ^{13}C NMR (125 MHz, DMSO): δ=146.9, 144.9, 144.3, 137.2, 129.3, 128.3, 128.2, 127.9, 127.4, 127.1, 126.9, 126.6, 98.3, 91.1, 82.7, 80.3, 76.8, 73.8, 71.4, 67.2, 40.6, 40.4, 40.2, 40.1, 32.6, 31.2, 23.7, 23.5, 20.8. UV/Vis λ_{max} 271, 412, 479 nm; HRMS (ESI): *m/z* calculated for $C_{30}H_{32}IN_2O_2OsS$ [M+H$^+$]: 803.0839; found: 803.0845. Elemental analysis: found (calculated for $C_{30}H_{31}N_2IO_2OsS$): C 44.71 (45.00), H 3.80 (3.90), N 3.49 (3.50).

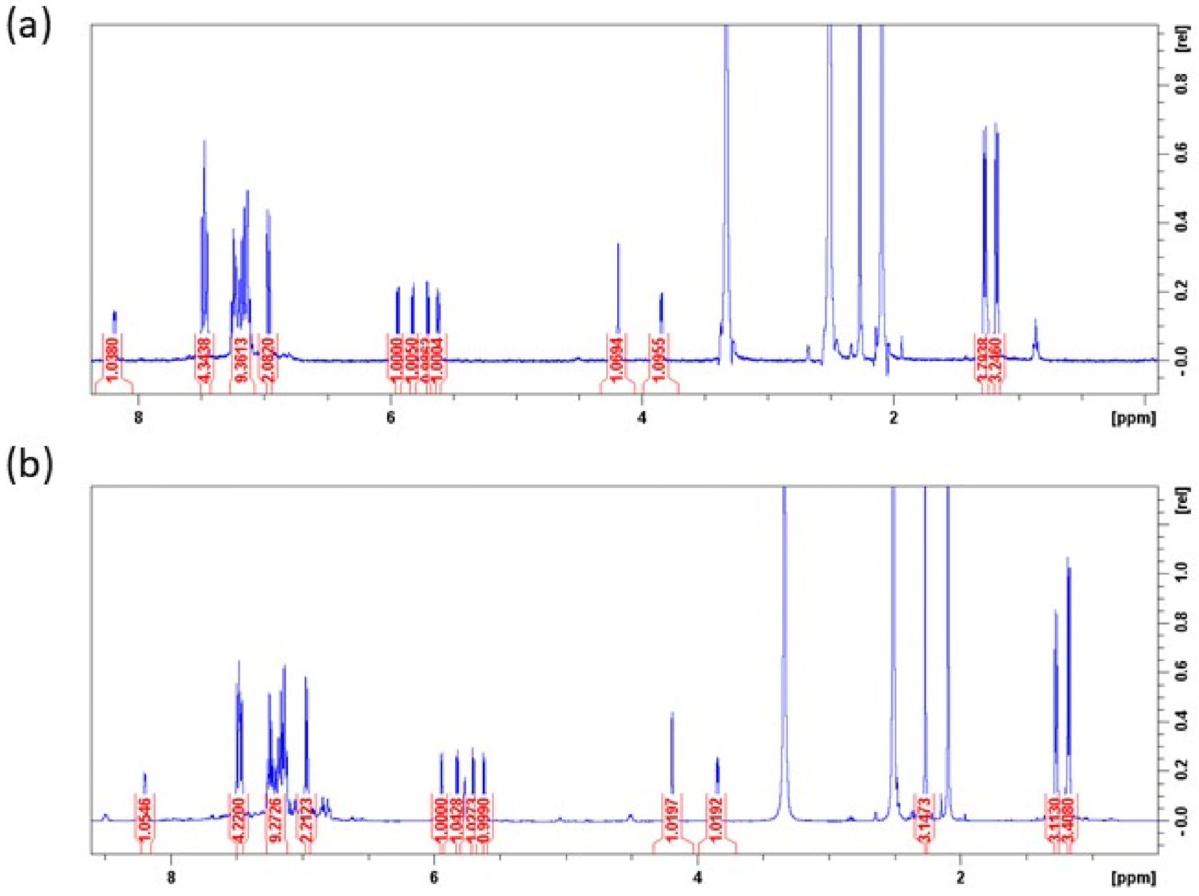

Figure S5. 500 MHz ^1H NMR spectra of (a) ***SS*-3** and (b) ***RR*-3** in d_6-DMSO.

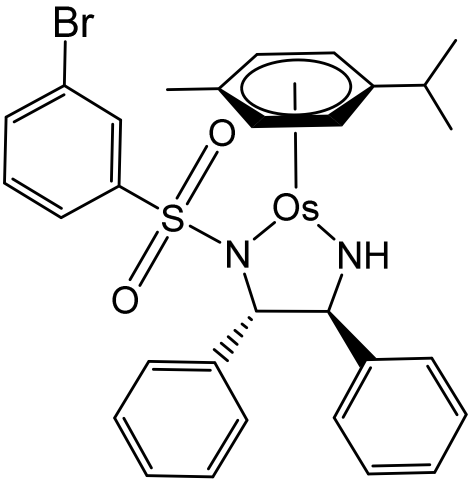

***SS*-5**

[Os(η^6-*p*-cymene)((1*S*, 2*S*)-*m*-BrPh-DPEN)] (*SS*-5). To a stirred solution of osmium *p*-cymene chloride dimer (50 mg, 0.06 mmol) in DCM (5 mL) was added (*1S*, *2S*)-*m*-BrPh-DPEN (***SS*-L3**, 19 mg, 0.05 mmol). A bright yellow solution was formed. Potassium hydroxide (50 mg) was added, followed by distilled water (30 mL) and brick-red organic layer was formed. The organic layer was extracted with DCM (3 x 10 mL) and the resulting red solution was concentrated *in vacuo*. A bright orange solid was obtained (0.043 g, 0.06 mmol, 93%). ^{1}H NMR (400 MHz, d_6-DMSO, 25 °C, TMS): δ=8.29 (d, ^{3}J(H,H)=3.8 Hz, 1H, NH), 7.55 (d, ^{3}J(H,H)=8.5 Hz, 1H, ArH), 7.46 (d, ^{3}J(H,H)=7.1 Hz, 2H, ArH), 7.40 (s, 1H, ArH), 7.31 (d, ^{3}J(H,H)=8.0 Hz, 1H, ArH), 7.24 (d, ^{3}J(H,H)=7.4 Hz, 2H, ArH), 7.22-7.13 (m, 7H, ArH), 5.92 (d, ^{3}J(H,H)=5.7 Hz, 1H, Os-ArH), 5.80 (d, ^{3}J(H,H)=5.7 Hz, 1H, Os-ArH), 5.67 (d, ^{3}J(H,H)=5.2 Hz, 1H, Os-ArH), 5.58 (d, ^{3}J(H,H)=5.6 Hz, 1H, Os-ArH), 4.23 (s, 1H, TsNCH), 3.85 (d, ^{3}J(H,H)=4.4 Hz, 1H, CH), 2.29 (s, 3H, $CH(CH_3)_2$), 2.27 (d, ^{3}J(H,H)=6.8 Hz, 3H, $CH(CH_3)_2$), 1.20 (d, ^{3}J(H,H)=6.8 Hz, 3H, CH_3). ^{13}C NMR (125 MHz, d_6-DMSO): δ=146.8, 146.7, 144.8, 133.8, 130.7, 129.3, 129.1, 128.3, 128.0, 127.3, 126.7, 126.6, 125.6, 121.7, 91.1, 82.8, 80.3, 77.0, 73.6, 71.5, 71.1, 67.6, 40.6, 40.5, 40.4, 33.5, 32.6, 24.5, 23.7, 23.6, 21.0, 20.8. UV/Vis λ_{max} 272, 412, 480 nm; $C_{30}H_{32}BrN_2O_2OsS$ [M+H$^+$]: 755.0977; found: 755.0956. C 46.93 (47.80), H 3.56 (4.15), N 3.58 (3.72).

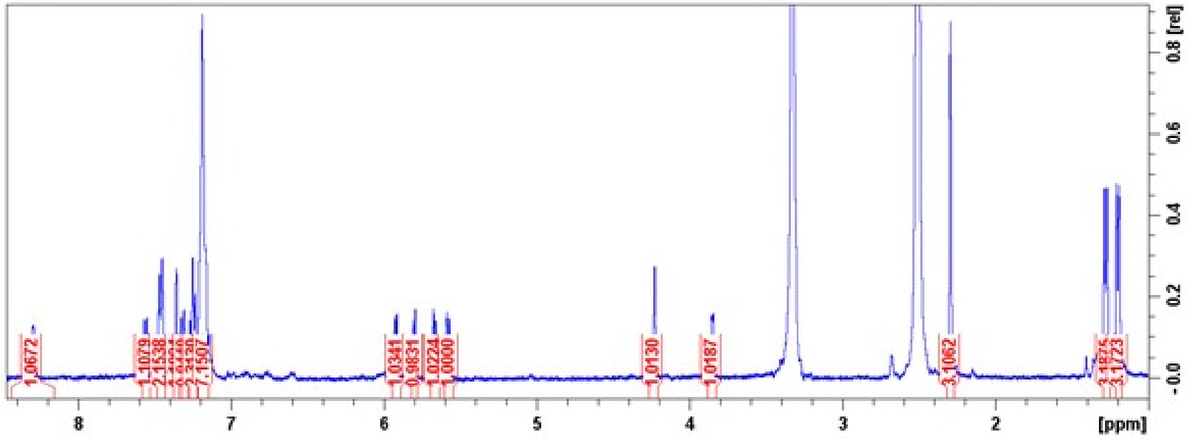

Figure S6. 500 MHz ^{1}H NMR spectra of ***SS*-5** in d_6-DMSO

ES4 X-ray crystal structures

***SS*-2** and ***SS*-3** were recrystallized in a minimum amount of dichloromethane and hexane (1:2) at room temperature. A single crystal was mounted on a glad fibre with oil and analysed on a Oxford Diffraction Gemini diffractometer with a ruby charge-coupled detector at 150 K. The structure was determined using Olex2,4 and the ShelXA5 solution programme through 'Direct' calculation, before further refinement using ShelXL5 'Least Square' method. ***SS*-2** was chiral (enantiospecific reaction product) so the structure was refined using BASF.TWIN in Shelx2014 to give low Flack parameters. Data were visualized and analysed in Mercury 3.3 software.

X-ray crystallographic data for complexes ***SS*-1**,[3] ***SS*-2** and ***SS*-3** have been deposited in the Cambridge Crystallographic Data Centre under the accession numbers CCDC 1035612, 2047054 and 2047055, respectively. X-ray crystallographic data in CIF format are available from the Cambridge Crystallographic Data Centre (http://www.ccdc.cam.ac.uk/). Complexes ***SS*-2** and ***SS*-3** had orthorhombic crystal systems, with $P2_12_12_1$ space groups and were closely comparable to ***SS*-1** (CCDC number 1035612). The *para*-cymene was coordinated to the osmium centre via η^6-coordination, resulting in a pseudo-octahedral coordination geometry (**Table S1**, **Figure S7** and **S8**).

Table S1. X-ray crystallographic data for ***SS*-1** (*p*-CH_3), ***SS*-2** (*p*-Br) and ***SS*-3** (*p*-I).

**Complex**	***SS*-1**[a]	***SS*-2**	***SS*-3**
a **(Å)**	10.6100(3)	9.9784(1)	9.33638(14)
b **(Å)**	13.8464(3)	14.2370(1)	14.31887(15)
c **(Å)**	18.9530(5)	19.7954(2)	21.7165(3)
α **(°)**	90	90	90
β **(°)**	90	90	90
γ **(°)**	90	90	90
**Crystal system**	Orthorhombic	Orthorhombic	Orthorhombic
**Temperature (K)**	150	150	150
**Reported volume (Å^3)**	2788.79(11)	2812.18(4)	2903.21(6)
**Space group**	$P2_12_12_1$	$P2_12_12_1$	$P2_12_12_1$
**D_x (g cm^{-3})**	1.641	1.780	1.832
**Z**	4	4	4
**Empirical formula**	$C_{31}H_{34}N_2O_2OsS$	$C_{30}H_{31}BrN_2O_2OsS$	$C_{30}H_{31}IN_2O_2OsS$
**MW (g/mol)**	688.86	754.74	800.73
**μ (mm^{-1})**	4.678	11.157	17.545
**F(000)**	1368.0	1472	1544
**Reflections measured**	33345	5531	5624
**WR_2 reflections**	0.0927	0.0771	0.0665

[a] Literature x-ray crystallographic data for ***SS*-1**.[3]

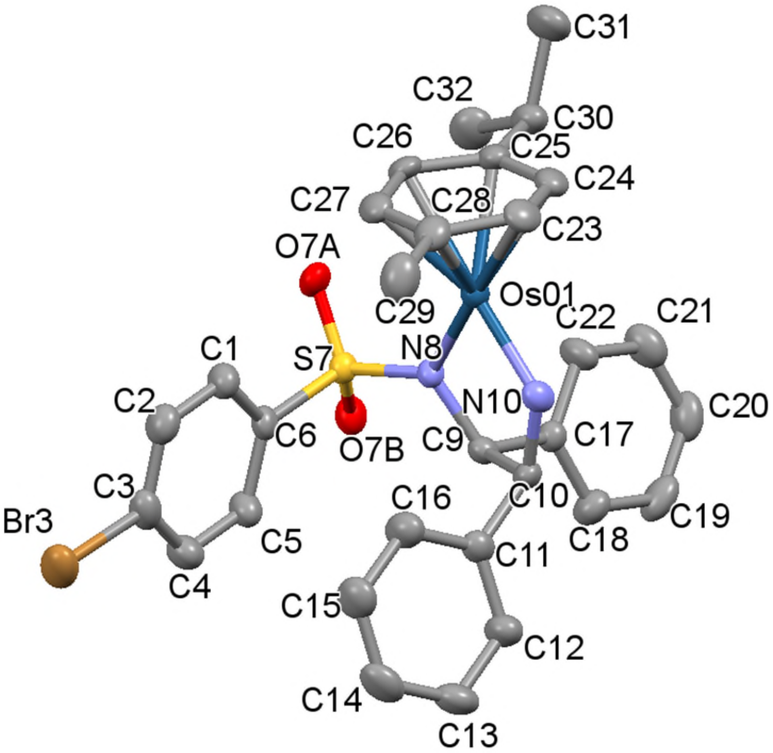

Figure S7. ORTEP x-ray crystal structures of ***SS*-2** (CCDC number 2047054) showing carbon (■), nitrogen (■), oxygen (■), sulfur (■), osmium (■), bromine (■), as viewed in Mercury 3.3 software. Thermal ellipsoids (atomic displacement parameters) are presented at 50% probability level.

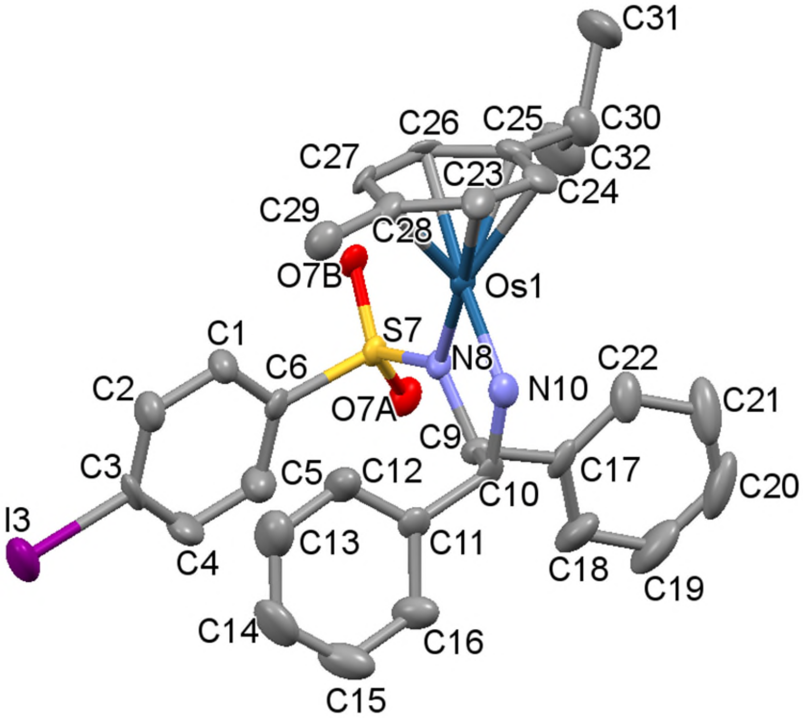

Figure S8. ORTEP x-ray crystal structure of ***SS*-3** (CCDC number 2047055) showing carbon (■), nitrogen (■), oxygen (■), sulfur (■), osmium (■), iodine (■), as viewed with Mercury 3.3 software. Thermal ellipsoids (atomic displacement parameters) are presented at 50% probability level.

ES5 Lipophilicity (Log P)

Octanol-saturated water (OSW) and water-saturated octanol (WSO) were prepared by mixing equivalent volumes of octanol and milliQ water for 24 h. The aqueous and organic phases were extracted. Osmium complex (~3-4 mg) was dissolved in OSW (10 mL) in duplicate, shaken and sonicated until the solution was fully saturated. Once saturated, the solution was syringe filtered to remove any excess solid. From the filtered solution, 2 mL was mixed with WSO (2 mL) and shaken for 24 h and performed in triplicate. The remaining filtered solution was analysed by ICP-MS (1:10 and 1:100 dilutions) in 3.6% *v*/*v* stabilised nitric acid (380.6 mg thiourea, 50 mg L-ascorbic acid, 25 mL 72% *v*/*v* nitric acid and 475 mL milliQ water) to calculate the 'before' concentration (concentration in filtered OSW). After 24 h, the OSW (water layer) bottom layer was extracted using a 2 mL syringe. The resulting samples were diluted appropriately in 3.6% stabilized nitric acid to fit the 0-1000 ppb calibration range and analysed by ICP-MS. LogP values were calculated using **Equation 1**. The apparent Log P of complexes **1**-**5** were determined using the shake-flask method in octanol and water, followed by elemental analysis by ICP-MS (**Table S2**).

$$\mathrm{LogP} = \frac{[\mathrm{Os\ in\ octanol}]}{[\mathrm{Os\ in\ water}]} = \frac{[\mathrm{Os\ in\ water\ before}] - [\mathrm{Os\ in\ water\ after}]}{[\mathrm{Os\ in\ water\ after}]}$$

Equation 1. Determination of the partition coefficient (LogP) as the ratio of concentrations of osmium complexes in octanol/water using ICP-MS under nitric acid conditions.

Table S2. Octanol-water partition coefficients (Log P) for complexes **1**-**5**, and **cisplatin**. All experiments were performed in triplicate.

Complex	Metal	R_1/R_2	Log P
***RR*-1**	Os	*p*-Me	1.45±0.02[a]
***SS*-2**	Os	*p*-Br	1.01±0.01
***RR*-2**	Os	*p*-Br	1.02±0.02
***SS*-3**	Os	*p*-I	1.02±0.03
***RR*-3**	Os	*p*-I	1.01±0.01
***RR*-4**	Os	*p*-F	0.30±0.03[a]
***SS*-5**	Os	*m*-Br	1.04±0.02
**Cisplatin**	Pt	-	-2.21±0.06[b]

[a]Literature Log P value of ***RR*-1** and ***RR*-4**.[4] [b] Literature Log P value of **cisplatin**.[5]

ES6 Density Functional Theory (DFT)

DFT calculations were carried out using Gaussian 03W software. Geometry optimization (lowest energy conformation) was calculated in the gas-phase using hybrid PBE0 (Perdew-Burke-Ernzerhof functional) with a combination of 75% DFT (Density functional theory) and 25% (Hartree Fock) computational methods. The Lan12DZ basis set including the effective core potential was used for Os and 6-31+G** basis set for all non-metal elements. Structures were optimized based on the x-ray crystallographic data for ***RR*-1** and were altered appropriately using GaussView 3.0. EPS (electrostatic potential surfaces were mapped to represent the electron density (isovalue = 0.04), ranging from red to blue (-0.025 to +0.250 au; **Table S4-6**). Mulliken partial chargers for complexes ***RR*-2** (R_1=Br) and ***RR*-3** (R_1=I) and ***RR*-4** (R_1=F) were calculated using DFT at the PBE0/Lanl2DZ/6-31+G** level of theory (**Table S3**, **Figure S9**).

Table S3. Mulliken partial charges calculated for complexes ***RR*-2** (X=Br) and ***RR*-3** (X=I) and ***RR*-4** (X=F), at the PBE0/Lanl2DZ/6-31+G** level of theory.

	**Mulliken Partial Charge**		
Atom	***RR*-2**	***RR*-3**	***RR*-4**
Os	0.566	0.566	0.566
S	1.371	1.371	1.369
O	-0.597	-0.597	-0.599
O	-0.550	-0.550	-0.551
N	-0.889	-0.889	-0.891
N	-0.820	-0.821	-0.821
X	0.065	0.172	-0.323

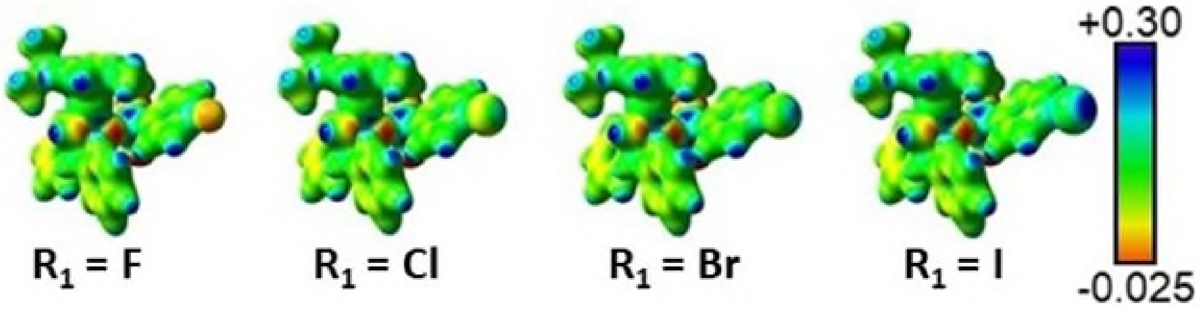

Figure S9. Calculated electrostatic potential energy surfaces for complexes ***RR*-2** (X=Br), ***RR*-3** (X=I), **modelled complex** (X=Cl) and ***RR*-4** (X=F) at the PBE0/Lanl2DZ/6-31+G** level of theory. EPS shown mapped onto total electron density. Isovalue = 0.04. (*R*,*R*)-catalyst only. Surface mapping colours range from red (-0.025 au) to blue (+0.30 au).

Table S4. Cartesian coordinates and electrostatic potential surface (EPS) calculated for ***RR*-2** (X = Br) calculated at the PBE0/Lanl2DZ/6-31+G** level. EPS shown mapped onto total electron density. Isovalue = 0.04. (*R*,*R*)-catalyst only. Surface mapping colours range from red (-0.025 au) to blue (+0.30 au).

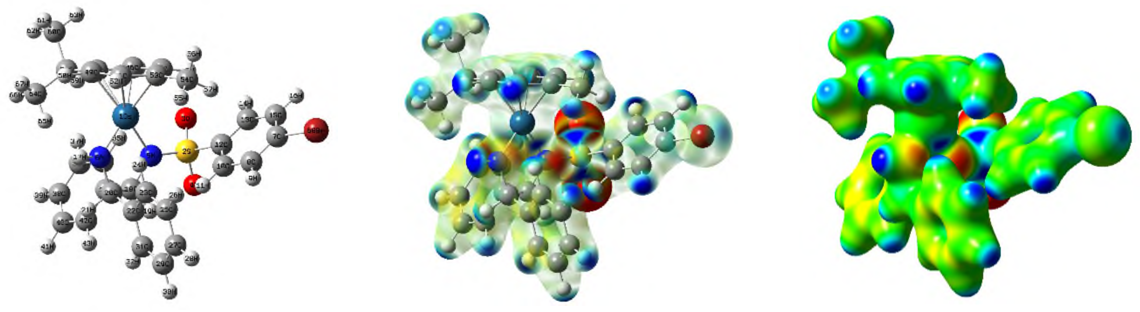

Atom	x	y	z
Os 1	-1.0486	-0.8113	-0.6906
S 2	0.2446	0.3755	2.0625
O 3	-0.2102	-0.8538	2.7228
O 4	0.2540	1.6268	2.8207
N 5	-0.5498	0.6199	0.6479
N 6	-1.5834	0.7881	-1.6005
C 7	4.5748	-0.3864	0.9239
C 8	3.8801	0.6265	0.2721
H 9	4.3653	1.2356	-0.4823
C 10	2.5468	0.8444	0.6017
H 11	1.9821	1.6193	0.0934
C 12	1.9336	0.0449	1.5629
C 13	2.6434	-0.9586	2.2160
H 14	2.1441	-1.5588	2.9704
C 15	3.9804	-1.1830	1.8940
H 16	4.5473	-1.9624	2.3908
H 17	-1.9529	0.8207	-2.5432
C 18	-1.2107	1.9167	0.4694
H 19	-0.5917	2.7061	0.9094
C 20	-1.2818	2.0991	-1.0579
H 21	-2.1103	2.7864	-1.2777
C 22	-0.0113	2.7213	-1.6286
C 23	0.8691	2.0065	-2.4404
H 24	0.6375	0.9716	-2.6745
C 25	2.0311	2.6028	-2.9300
H 26	2.7056	2.0300	-3.5613
C 27	2.3277	3.9249	-2.6149
H 28	3.2326	4.3903	-2.9952
C 29	1.4500	4.6515	-1.8113
H 30	1.6690	5.6859	-1.5616
C 31	0.2909	4.0546	-1.3290
H 32	-0.3880	4.6313	-0.7034
C 33	-2.5914	2.0024	1.1110
C 34	-3.1575	0.9444	1.8213

Atom	x	y	z
H 35	-2.5927	0.0256	1.9443
C 36	-4.4217	1.0703	2.3955
H 37	-4.8428	0.2366	2.9514
C 38	-5.1369	2.2567	2.2735
H 39	-6.1207	2.3540	2.7239
C 40	-4.5719	3.3267	1.5829
H 41	-5.1112	4.2659	1.4954
C 42	-3.3106	3.1980	1.0122
H 43	-2.8751	4.0480	0.4896
C 44	-0.0790	-2.6675	0.1747
H 45	0.6322	-2.6606	0.9920
C 46	-1.4587	-2.6585	0.4771
H 47	-1.7666	-2.6541	1.5161
C 48	-2.4353	-2.4698	-0.5569
C 49	-1.9852	-2.3968	-1.9134
H 50	-2.6994	-2.2138	-2.7081
C 51	-0.6015	-2.3204	-2.1992
H 52	-0.2711	-2.1184	-3.2131
C 53	0.3790	-2.4270	-1.1472
C 54	1.8401	-2.3348	-1.4631
H 55	2.0235	-1.6270	-2.2759
H 56	2.2158	-3.3156	-1.7788
H 57	2.4153	-2.0130	-0.5925
C 58	-3.9026	-2.4175	-0.1933
H 59	-3.9466	-2.0536	0.8414
C 60	-4.4854	-3.8344	-0.2315
H 61	-4.4559	-4.2406	-1.2487
H 62	-5.5297	-3.8263	0.0961
H 63	-3.9297	-4.5164	0.4194
C 64	-4.7154	-1.4520	-1.0509
H 65	-4.2623	-0.4561	-1.0505
H 66	-5.7314	-1.3677	-0.6538
H 67	-4.8039	-1.8005	-2.0863
Br 68	6.4409	-0.7006	0.4586

Table S5. Cartesian coordinates and electrostatic potential surface (EPS) calculated for ***RR*-3** (X=I) calculated at the PBE0/Lanl2DZ/6-31+G** level. EPS is shown mapped onto the total electron density. Isovalue = 0.04. (*R*,*R*)-catalyst only. Surface mapping colours range from red (-0.025 au) to blue (+0.30 au).

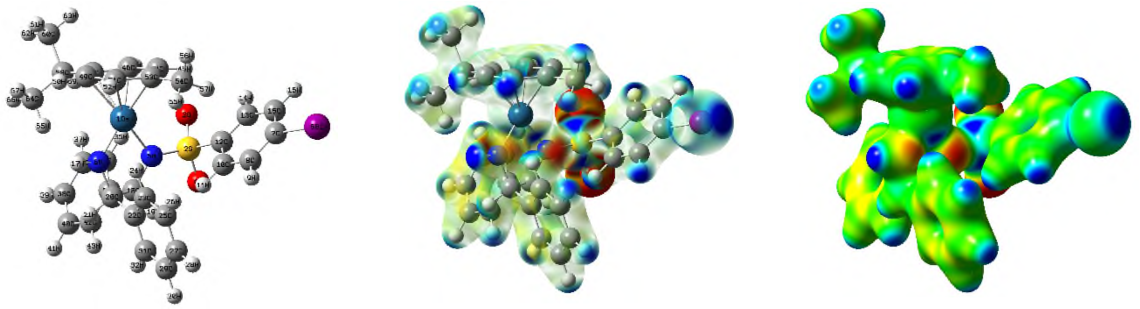

Atom	x	y	z
Os 1	-1.3565	-0.8153	-0.6914
S 2	-0.0670	0.3984	2.0515
O 3	-0.4760	-0.8468	2.7125
O 4	-0.0865	1.6462	2.8155
N 5	-0.8846	0.6254	0.6472
N 6	-1.9504	0.7711	-1.5873
C 7	4.2775	-0.2185	0.8501
C 8	3.5297	0.7639	0.2060
H 9	3.9737	1.3894	-0.5605
C 10	2.1939	0.9362	0.5530
H 11	1.5929	1.6854	0.0477
C 12	1.6240	0.1221	1.5278
C 13	2.3790	-0.8514	2.1747
H 14	1.9132	-1.4659	2.9391
C 15	3.7184	-1.0284	1.8332
H 16	4.3130	-1.7869	2.3305
H 17	-2.3367	0.7962	-2.5235
C 18	-1.5929	1.8991	0.4854
H 19	-0.9968	2.7065	0.9245
C 20	-1.6864	2.0882	-1.0397
H 21	-2.5398	2.7485	-1.2466
C 22	-0.4438	2.7566	-1.6204
C 23	0.4432	2.0821	-2.4592
H 24	0.2400	1.0444	-2.7065
C 25	1.5777	2.7213	-2.9592
H 26	2.2580	2.1792	-3.6109
C 27	1.8396	4.0469	-2.6281
H 28	2.7229	4.5456	-3.0165
C 29	0.9546	4.7334	-1.7978
H 30	1.1466	5.7700	-1.5350
C 31	-0.1768	4.0937	-1.3049
H 32	-0.8616	4.6396	-0.6584
C 33	-2.9687	1.9325	1.1421
C 34	-3.4870	0.8531	1.8569

Atom	x	y	z
H 35	-2.8878	-0.0447	1.9708
C 36	-4.7478	0.9318	2.4467
H 37	-5.1309	0.0823	3.0062
C 38	-5.5075	2.0913	2.3362
H 39	-6.4884	2.1518	2.7991
C 40	-4.9908	3.1823	1.6407
H 41	-5.5653	4.1012	1.5613
C 42	-3.7327	3.1009	1.0544
H 43	-3.3353	3.9673	0.5284
C 44	-0.3244	-2.6433	0.1626
H 45	0.3890	-2.6160	0.9773
C 46	-1.7026	-2.6778	0.4707
H 47	-2.0064	-2.6862	1.5109
C 48	-2.6892	-2.5171	-0.5589
C 49	-2.2470	-2.4261	-1.9169
H 50	-2.9698	-2.2646	-2.7086
C 51	-0.8677	-2.3028	-2.2074
H 52	-0.5482	-2.0860	-3.2217
C 53	0.1205	-2.3829	-1.1600
C 54	1.5757	-2.2384	-1.4829
H 55	1.7297	-1.5048	-2.2788
H 56	1.9796	-3.1988	-1.8253
H 57	2.1470	-1.9191	-0.6088
C 58	-4.1562	-2.5132	-0.1907
H 59	-4.2084	-2.1573	0.8464
C 60	-4.6943	-3.9474	-0.2352
H 61	-4.6580	-4.3459	-1.2553
H 62	-5.7365	-3.9743	0.0981
H 63	-4.1140	-4.6160	0.4080
C 64	-5.0017	-1.5686	-1.0400
H 65	-4.5818	-0.5582	-1.0319
H 66	-6.0196	-1.5214	-0.6416
H 67	-5.0794	-1.9113	-2.0782
I 68	6.3183	-0.4877	0.3171

Table S6. Cartesian coordinates and electrostatic potential surface (EPS) calculated for ***RR*-4** (X= F) calculated at the PBE0/Lanl2DZ/6-31+G** level. EPS is shown mapped onto the total electron density. Isovalue = 0.04. (*R*,*R*)-catalyst only. Surface mapping colours range from red (-0.025 au) to blue (+0.30 au).

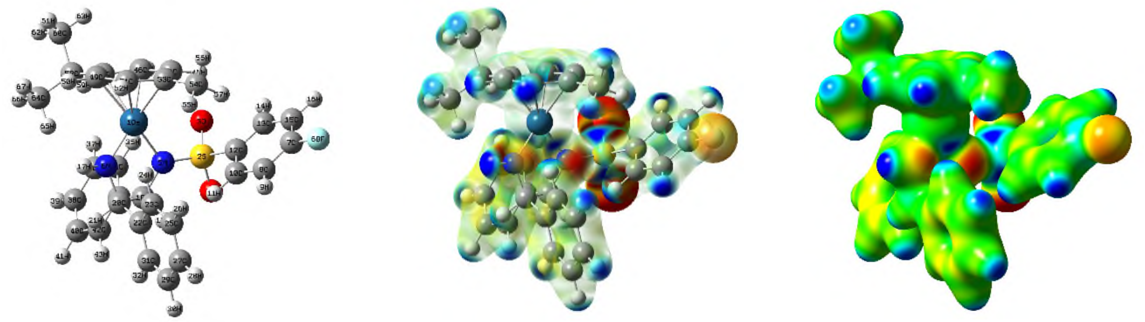

Atom	x	y	z
Os 1	-0.6540	-0.7843	-0.6626
S 2	0.7115	0.2380	2.1218
O 3	0.0200	-0.8779	2.7792
O 4	0.8909	1.4910	2.8562
N 5	0.0344	0.5768	0.6629
N 6	-0.8765	0.8563	-1.6271
C 7	4.8976	-1.2587	1.2232
C 8	4.4061	-0.1790	0.4999
H 9	5.0303	0.2991	-0.2473
C 10	3.1141	0.2636	0.7541
H 11	2.7022	1.0948	0.1913
C 12	2.3380	-0.3848	1.7132
C 13	2.8472	-1.4611	2.4362
H 14	2.2233	-1.9400	3.1843
C 15	4.1416	-1.9094	2.1885
H 16	4.5695	-2.7456	2.7310
H 17	-1.1952	0.9232	-2.5864
C 18	-0.3919	1.9608	0.4335
H 19	0.3366	2.6472	0.8788
C 20	-0.3833	2.1137	-1.0993
H 21	-1.0820	2.9207	-1.3600
C 22	0.9861	2.5123	-1.6403
C 23	1.7889	1.6338	-2.3681
H 24	1.4249	0.6281	-2.5578
C 25	3.0415	2.0334	-2.8340
H 26	3.6528	1.3355	-3.4005
C 27	3.5084	3.3188	-2.5793
H 28	4.4833	3.6308	-2.9431
C 29	2.7117	4.2068	-1.8579
H 30	3.0639	5.2145	-1.6556
C 31	1.4624	3.8058	-1.3989
H 32	0.8466	4.5074	-0.8390
C 33	-1.7578	2.2925	1.0247
C 34	-2.5191	1.3547	1.7215

Atom	x	y	z
H 35	-2.1229	0.3546	1.8677
C 36	-3.7600	1.7012	2.2537
H 37	-4.3343	0.9584	2.8013
C 38	-4.2570	2.9913	2.1021
H 39	-5.2224	3.2611	2.5208
C 40	-3.4964	3.9406	1.4228
H 41	-3.8640	4.9572	1.3122
C 42	-2.2585	3.5918	0.8940
H 43	-1.6680	4.3487	0.3805
C 44	-0.1004	-2.7557	0.3184
H 45	0.5294	-2.8456	1.1955
C 46	-1.4752	-2.4890	0.4993
H 47	-1.8639	-2.4001	1.5071
C 48	-2.3165	-2.1701	-0.6209
C 49	-1.7516	-2.2266	-1.9334
H 50	-2.3546	-1.9494	-2.7905
C 51	-0.3561	-2.3953	-2.0967
H 52	0.0872	-2.2852	-3.0813
C 53	0.5010	-2.6387	-0.9616
C 54	1.9775	-2.8007	-1.1502
H 55	2.3403	-2.1619	-1.9598
H 56	2.2123	-3.8407	-1.4071
H 57	2.5203	-2.5390	-0.2399
C 58	-3.7743	-1.8475	-0.3796
H 59	-3.8208	-1.3884	0.6164
C 60	-4.5851	-3.1478	-0.3515
H 61	-4.5564	-3.6450	-1.3274
H 62	-5.6324	-2.9395	-0.1113
H 63	-4.1980	-3.8484	0.3946
C 64	-4.3567	-0.8449	-1.3709
H 65	-3.7412	0.0586	-1.4177
H 66	-5.3648	-0.5568	-1.0586
H 67	-4.4417	-1.2685	-2.3783
F 68	6.1412	-1.6882	0.9780

ES7 Asymmetric Transfer Hydrogenation (ATH)

^{1}H NMR Asymmetric Transfer Hydrogenation (ATH)

Osmium catalyst (5 µmol, 0.5% mol eq.) was weighed into a small glass vial and dissolved in d_6-benzene (100 µL) and 5:2 formic acid/TEA azeotrope (500 µL) under nitrogen at 310 K. The mixture was stirred for *ca.* 30 min. Acetophenone (1 mmol, 200 mol eq.) was added and the mixture transferred to a nitrogen-purged 5 mm NMR tube. A ^{1}H NMR spectrum was recorded every 2 min for 1 h and the performed in triplicate. ^{1}H NMR spectra were also taken every 10 min for 15 h.

GC-FID Asymmetric Transfer Hydrogenation (ATH)

Formic acid: TEA 5:2 azeotrope (500 µL) was added to round-bottom flask containing osmium catalyst (5 µmol, 1 eq.) under nitrogen and acetophenone (1 mmol, 0.17 mL, 200 eq.) and stirred at 310K for 24 h. From the resulting solution, 2-3 drops were placed in 1:1 ethyl acetate: sodium bicarbonate (2 mL). The organic ethyl acetate layer was extracted and filtered through a silica micro pipette column. Conversion and enantiomeric excess were determined by chiral GC analysis (CROMPAC CYCLODEXTRIN-β- 236M-19, 50 m × 0.25 mm × 0.25 µm, gas hydrogen, T = 383 K, P = 15 *psi*, FID temp 523 K, injector temp 493 K): ketone 9.5 min, *R* isomer 14.9 min, *S* isomer 15.9 min.

ES8 *In vitro* antiproliferative screening

Culture medium. DMEM was supplemented with 10% fetal calf serum (FCS), 1% penicillin/streptomycin and 1% L-glutamine (2 mM) solution and incubated at 310 K prior to use. DMEM was prepared by the technical staff at the School of Life Sciences, and contains 0.2 mM of L-cystine (oxidized).

Defrosting cells. A frozen ampoule of cells (1×10^6 cells per vial) stored in liquid nitrogen was placed in dry ice and the cells rapidly defrosted in a 310 K water bath. Once defrosted, the cell solution was transferred to a falcon tube and re-suspended in DMEM (5 mL), followed by centrifugation (298 K, 5 min, 1000 rpm). The supernatant was removed, the pellet re-suspended in DMEM (3 mL) and transferred to a T25 cell culture flask before incubating for 24-48 h (310 K, 5% CO_2).

General cell maintenance. Upon 80-90% cell coverage onto the surface of the culture flask, the supernatant was removed, cells washed PBS and 0.25% trypsin/EDTA (1 mL) was added (5 min, 310 K). Once cells were in solution, DMEM was added to quench trypsin activity and pipetted to form a single cell suspension, which was then transferred to a T75 culture flask for further incubation (310 K, 5% CO_2).

Sulforhodamine B (SRB) Assay. The antiproliferative activity of complexes was determined using the well-established colorimetric SRB assay.[6] At 80-90% confluence of cells, a single cell suspension was obtained as previously described. Cells were counted in duplicate using haemocytometer (2 × 10 µL) and two 96-well F-bottom plates were seeded (5000 cells/ well in 150 µL) and incubated for 48 h (310 K, 5% CO_2). Stock solutions of osmium (150 µM) and CDDP (1000 µM) complexes were prepared in 5% *v*/*v* DMSO and 95% *v*/*v* culture media and diluted to 6 concentrations in the range 0.01-150 µM. Note that all untreated control cells were exposed to 5% *v*/*v* DMSO and 95% *v*/*v* culture media, which did not affect the cell viability compared to cells exposed to just culture media. The supernatant media from each plate was removed with suction and solutions of osmium or platinum were added to each well (in triplicate) on each plate and incubated for 24 h. The supernatant was removed, plates washed with PBS and cells incubated in complex-free DMEM for 72 h (310 K, 5% CO_2). 50 % TCA (50 µL) was added to each well and incubated at 277 K for 1 h. The plates were washed (10×) with water and air-dried. 0.4% SRB dye prepared in 1 % acetic acid (50 µL) was added to each well and left to stand (30 min). The plates were washed with 1 % acetic acid (7×) and heat-dried. 10 mM Tris Base pH 10.5 (150 µL) was added to each well and left to stand for 1 h. The UV absorbance at 492 nm was measured using SkanIt multiplate analyse. Data were processed in Excel and plotted as a sigmoidal dose-response curve in Origin Pro 2016 normalised to the untreated (control) wells.

Table S7. Half-maximal inhibitory concentrations (IC_{50} / µM) of **1-5** and cisplatin against A2780 (ovarian), A549 (lung), PC3 (prostate) and MCF7 (breast) cancer cells and MRC5 healthy lung fibroblasts upon 24 h drug exposure, followed by 72 h recovery (drug-free media).

Complex	R	A2780	A549	PC3	MCF7	MRC5
***RR*-1**[a]	*p*-CH_3	15.5±0.5	21.1±0.3	12±0.3	10.9±0.7	21.4±0.7
***SS*-2**	*p*-Br	31±2	31±1	41±2	32±1	37.8±0.7
***RR*-2**	*p*-Br	27.4±0.6	29.5±0.5	38.9±4.3	26.3±4.6	37±1
***SS*-3**	*p*-I	27.5±0.8	33±0.4	37±2	28±3	-
***RR*-3**	*p*-I	29±3	32±0.4	39.5±0.6	22.7±5.6	-
***RR*-4**[a]	*p*-F	17±1	-	-	-	-
***SS*-5**	*m*-Br	27±2	32±2	-	-	-
**Cisplatin**	-	1.2±0.3	3.2±0.1	4.1±0.5	6.6±0.4	12.8±0.5

[a] Literature IC_{50} values of ***RR*-1**, ***RR*-4** and **cisplatin** using the specified conditions.[7] Note that all precursor Ph-DPEN ligands were determined to be non-toxic (IC_{50}> 150 µM), thus, data are not displayed.

Formate and acetate-dependent cell viability. The formate and acetate-dependent cell viability of ***SS*-2** or ***RR*-2** in A549 and MRC5 cells was determined following the general SRB protocol but by co-administering with 0.5 or 1 × IC_{50} of ***SS-2*** or ***RR*-2** (30 µM) with sodium formate (0-2 mM in DMEM) in 200 µL per well (**Figure S10-12**).

	**Normalised cell viability (%)**			
	***SS*-2**		***RR*-2**	
[Formate] / mM	0.5 × IC_{50}	1.0 × IC_{50}	0.5 × IC_{50}	1.0 × IC_{50}
0.0	100 ± 3	100 ± 7	100 ± 2	100 ± 4
0.5	60 ± 12 (P<0.05)	42 ± 8 (P<0.05)	57 ± 7 (P<0.01)	46 ± 4 (P<0.01)
1.0	36 ± 8 (P<0.01)	20 ± 3 (P<0.05)	38 ± 8 (P<0.01)	34 ± 7 (P<0.01)
2.0	33 ± 3 (P<0.01)	23 ± 5 (P<0.05)	29 ± 11 (P<0.01)	28 ± 4 (P<0.01)

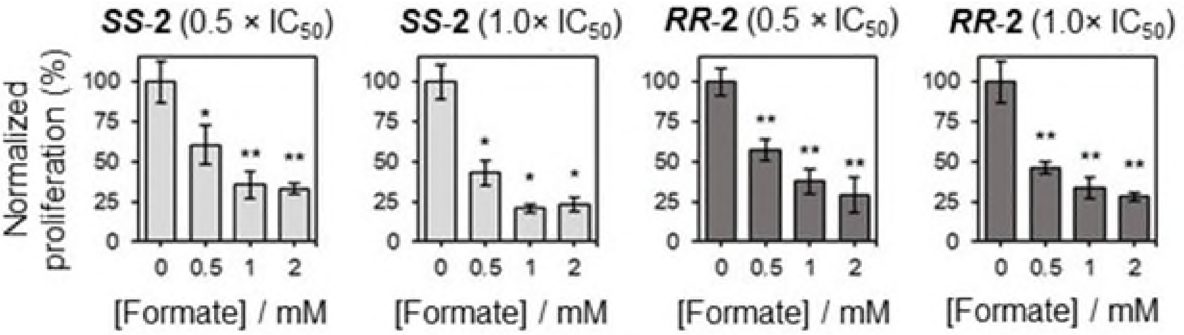

Figure S10. Normalized cell proliferation (%) of A549 (human lung) cancer cells treated with 0.5 or 1 × IC_{50} of ***SS*-2** or ***RR*-2** in A549 (lung) cancer cells upon co-administration with sodium formate (0-2 mM) for 24 h, followed by 72 h recovery in complex-free media. Statistical significance was calculated using a two-tailed t-test assuming unequal variances (Welch's t-test) compared to the untreated controls, $*p < 0.05$, $**p < 0.01$ and $***p < 0.001$.

	Normalized cell viability (%)			
	***SS*-2**		***RR*-2**	
[Acetate] / mM	0.5× IC_{50}	1.0× IC_{50}	0.5× IC_{50}	1.0× IC_{50}
0.0	100 ± 6	100 ± 7	100 ± 2	100 ± 4
0.5	97 ± 7 (P>0.05)	96 ± 5 (P>0.05)	100 ± 8 (P>0.05)	97 ± 5 (P>0.05)
1.0	102 ± 4 (P>0.05)	97 ± 5 (P>0.05)	97 ± 6 (P>0.05)	100 ± 7 (P>0.05)
2.0	99 ± 4 (P>0.05)	94 ± 8 (P>0.05)	98 ± 2 (P>0.05)	95 ± 5 (P>0.05)

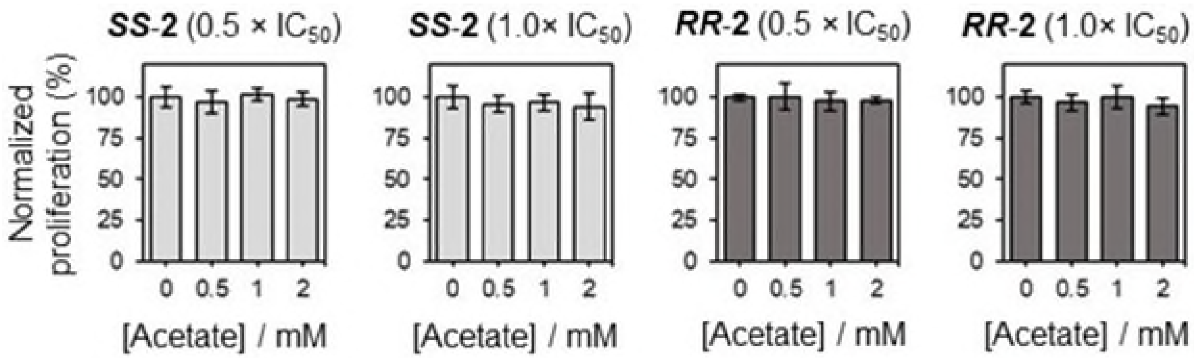

Figure S11. Normalized cell proliferation (%) of A549 (human lung) cancer cells treated with 0.5 - 1.0 × IC_{50} of ***SS*-2** or ***RR*-2** upon co-administration with sodium acetate (0-2 mM) for 24 h, followed by 72 h recovery in complex-free media. Statistical significance was calculated using a two-tailed t-test assuming unequal variances (Welch's t-test) compared to the untreated controls, *$p < 0.05$, **$p < 0.01$ and ***$p < 0.001$.

	Normalized cell viability (%)			
	***SS*-2 (0.5 × IC_{50})**		***RR*-2 (0.5 × IC_{50})**	
Conc/mM	Acetate	Formate	Acetate	Formate
0.0	100 ± 14	100 ± 12	100 ± 11	100 ± 10
0.5	97 ± 10 (P>0.05)	92 ± 16 (P>0.05)	92 ± 11 (P>0.05)	99 ±11 (P>0.05)
1.0	101 ± 10 (P>0.05)	100 ± 11 (P>0.05)	98 ± 8 (P>0.05)	103 ± 8 (P>0.05)
2.0	96 ± 10 (P>0.05)	95 ± 17 (P>0.05)	96 ± 9 (P>0.05)	89 ± 7 (P>0.05)

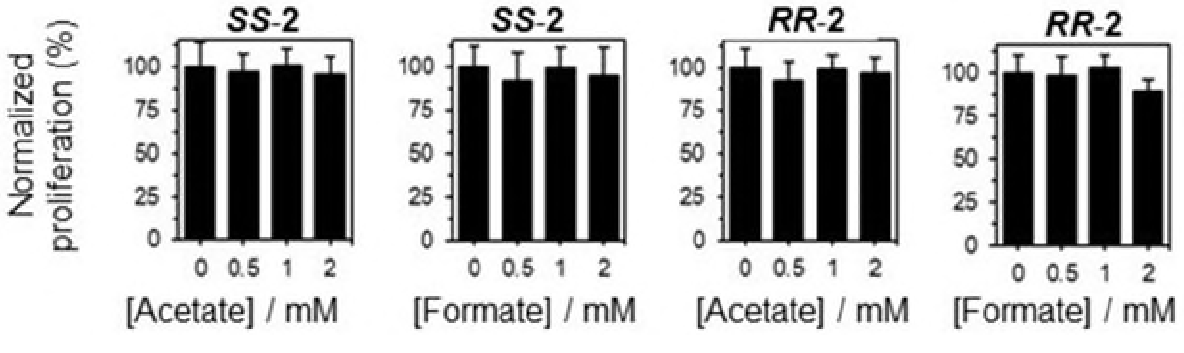

Figure S12. Normalized cell proliferation (%) MRC5 (healthy lung) fibroblasts treated with 0.5 × IC_{50} of ***SS*-2** or ***RR*-2** upon co-administration with sodium acetate or formate (0-2 mM) for 24 h, followed by 72 h recovery in drug-free media. Statistical significance was calculated using a two-tailed t-test assuming unequal variances (Welch's t-test) compared to the untreated controls, *$p < 0.05$, **$p < 0.01$ and ***$p < 0.001$.

ES9 Membrane integrity

Membrane integrity analysis. A cell suspension of 1 × 10^6 A549 cells were seeded in a 6-well plate for 24 h (310 K, 5% CO_2). The supernatant medium was removed and cells were treated with 30 µM of ***SS*-2** (5% *v*/*v* DMSO, 95% *v*/*v* DMEM) and 0-2 mM sodium formate (5% DMSO *v*/*v*, 95% *v*/*v* DMEM) for 24 h (310 K, 5% CO_2). Note that all untreated control cells were exposed to 5% *v*/*v* DMSO and 95% *v*/*v* culture media, which did not affect the cell viability compared to cells exposed to just culture media. The supernatant was removed, cells washed with PBS, and 0.25% trypsin/EDTA used to remove cells from the surface. The cell suspensions from each well were centrifuged (298 K, 1500 rpm, 5 min) to obtain pellets. The pellets were washed with PBS, before re-suspending in a staining buffer containing 80 µg/mL RNAse and 50 µg/mL propidium iodide (500 µL) for 30 min protected from light. The cells were centrifuged and the supernatant removed, before being re-suspended in PBS and analysed by flow cytometry (**Table S8**, **Figure S13**).

Table S8. Membrane integrity flow cytometry analysis for a normalized population of A549 (human lung) cancer cells treated with 1 × IC_{50} (30 µM) of ***SS*-2** for 24 h upon co-administration with sodium formate (0 or 2 mM). Statistical significance was calculated using a two-tailed t-test assuming unequal variances (Welch's t-test) compared to the untreated controls, *$p < 0.05$, **$p < 0.01$ and ***$p < 0.001$. No statistical differences in membrane viability was observed between the untreated controls and cells treated with ***SS*-2** (p>0.05), suggesting that ***SS*-2** (in the presence or absence of sodium formate) does not damage the cell membrane.

Complex	Viable membrane (FL2-)	Non-viable membrane (FL2+)
**Untreated control**	99.73±0.07	0.27±0.07
**Untreated + Formate (2 mM)**	99.76±0.05	0.24±0.05
***SS*-2**	99.82±0.11	0.18±0.11
***SS*-2 +Formate (2 mM)**	99.76±0.05	0.24±0.05

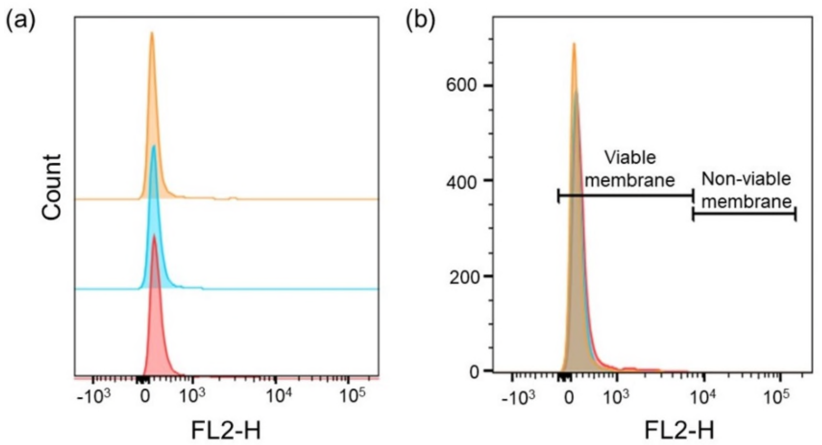

Figure S13. Membrane integrity assessed by PI (FL2) staining of A549 cancer cells treated with ***SS*-2** (1×IC_{50}) for 24 h (310 K, 5% CO_2) upon co-administration with sodium formate (0-2 mM). (a) untreated control (■), ***SS*-2** (■) and ***SS*-2** + 2 mM sodium formate (■); (b) Overlay of propidium idodide fluorescence with gated bars indicating viable (FL2-) and non-viable membranes (FL2+).

ES10 Cell cycle analysis

Cell cycle analysis. Cell pellets treated with 30 µM of ***SS*-2** were obtained as described in the membrane integrity procedure. The obtained pellets were washed in PBS before re-suspending in ice-cold ethanol (fixation) for 30 min. The supernatant ethanol was removed and the cells washed with PBS. The cells were then treated with 500 µL of staining buffer (as described for membrane integrity analysis) for 30 min. Cells were then pelleted by centrifugation, supernatant removed, before being re-suspended in PBS (**Table S9**).

Table S9. Cell cycle flow cytometry analysis for a normalized population of A549 (human lung) cancer cells treated with 1 × IC_{50} (30 µM) of ***SS*-2** for 24 h. Statistical significance was calculated using a two-tailed t-test assuming unequal variances (Welch's t-test) compared to the untreated controls, *$p < 0.05$, **$p < 0.01$ and ***$p < 0.001$. Cells treated with ***SS*-2** showed slight G_1-arrest compared to the untreated controls.

Complex	Sub G_1-phase	G_1 phase	S Phase	G_2/M Phase
**Untreated control**	1.28±0.23	70.40±0.97	20.70±0.67	7.99±0.51
	(*p*=1.0000)	(*p*=1.0000)	(*p*=1.0000)	(*p*=1.0000)
***SS*-2**	2.47±0.49	75.84±0.42	17.40±0.58	4.29±0.48
	(*p*=0.0353)	(*p*=0.0044)	(*p*=0.0032)	(*p*=0.0008)

[a] P-values calculated using a two-tailed t-test assuming unequal sample variance (Welch's t-test). All t-tests are compared to the untreated control.

ES11 ^{189}Os ICP-MS cellular accumulation studies

ICP-OES of drug stock solutions. Am osmium calibration (1000±10 µg/mL hexachlorodiammonium osmate in 15% *v*/*v* hydrochloric acid) in the range 0-700 ppb was prepared in 3.6% *v*/*v* nitric acid (supplemented with 10 mM thourea and 100 mg/L L-ascorbic acid to prevent formation of osmium tetroxide).[8] The calibration salinity was adjusted with sodium chloride to match the matrix of DMEM in the osmium stock solutions. Prior to all cellular accumulation experiments, solutions of osmium drug were prepared in 5% *v*/*v* DMSO: 95% *v*/*v* DMEM and their exact concentrations determined by ICP-OES.

General cellular accumulation protocol. A single cell suspension of cancer cells was obtained by following the general cell maintenance procedure and *ca*. 4 × 10^6 cells were seeded in cell culture Petri dish in media for 24 h. The cells were then treated with ***SS*-2** (5% *v*/*v* DMSO and 95% *v*/*v* DMEM) under variable conditions (concentration, time, temperature, formate, verapamil, methyl-β-cyclodextrin). After the specified time, the supernatant was removed using suction, the cells washed with PBS and detached with 0.25% trypsin/EDTA (310 K, 5 min), as previously described. Known volumes of DMEM were added to each Petri dish to form single cell suspension. Cells were counted in duplicate using a haemocytometer (2 ×10 µL) before centrifugation (298 K, 1500 rpm, 5 min) to obtain whole cell pellets. The supernatant was removed, the pellets re-suspended in PBS (1 mL) and centrifuged again (1300 rpm, 5 min). The supernatant was removed and the pellets stored at 253 K until ICP-MS analysis.

Nitric acid ICP-MS digestion. Cell pellets were digested in 72% nitric acid (200 μL) in an oven (347 K) for 12 h. The samples were diluted in 3.8 mL stabilised MilliQ water (containing 100 mg/L L-ascorbic acid and thiourea) so as not to exceed 0.1% *w*/*v* dissolved solids for ICP-MS analysis. ^{189}Os was monitored in no gas mode using an ^{166}Er (50 ppb) internal standard and calibrated to 0.1-1000 ppb osmium standard freshly prepared in 3.6% *v*/*v* nitric acid.

(i) ***Concentration.*** The concentration-dependent osmium cellular accumulation was determined using the general accumulation protocol. A2780 (ovarian) or A549 (lung) cancer cells were treated with 0.25, 0.5, 1 and 1.5 × the IC_{50} concentration (μM) ***SS*-2** for 24 h, no recovery (**Table S10**).

(ii) ***Time.*** The time-dependent osmium cellular accumulation was analysed for A2780 cells following the general cellular accumulation protocol. Cells were treated with 30 μM ***SS*-2** (10 mL) and incubated (310 K, 5% CO_2) for different drug exposure times (3, 6, 18 h, 24 h with no recovery and 24 h exposure with 24, 48 and 72 h recovery in drug-free media, **Table S11**).

(iii) ***Temperature.*** The temperature-dependent osmium cellular accumulation was analysed for A2780 cells following the general cellular accumulation protocol. Cells were treated with 30 μM ***SS*-2** (10 mL) for 3 or 6 h at 277 K or 310 K (**Table S12**).

(iv) ***Formate.*** The formate-dependent osmium cellular accumulation was analysed for A2780 cells following the general cellular accumulation protocol. Cells were treated with 30 μM of ***SS/RR*-2** and co-administered with 2 mM sodium formate (DMEM) for 24 h (310 K, 5% CO_2) with no recovery time in drug-free media (**Table S13**).

Table S10. Osmium (^{189}Os) ICP-MS cellular accumulation (ng Os / 10^6 cells) of **1-4** in A2780 (human ovarian) cancer cells when treated with equipotent 1 × IC_{50} for 24 h (no recovery).

Complex	R-group	ng Os / 10^6 cells
***SS*-1**[a]	CH_3	32±3
***RR*-1**[a]	CH_3	30±2
***SS*-2**	Br	29±3
***RR*-2**	Br	30±2
***SS*-3**	I	39±2
***RR*-3**	I	37±3
***RR*-4**[a]	F	10±2

[a] Literature values for the osmium cellular accumulation of ***SS*-1**, ***RR*-1** and ***RR*-4**.[7]

Table S11. Concentration-dependent osmium (^{189}Os) ICP-MS cellular accumulation in A2780 (human ovarian) cancer cells treated with 1 × IC_{50} of ***SS*-1**, ***SS*-2**, and ***RR*-2** for 24 h (no recovery). The osmium accumulation is the similar when treated with equipotent concentrations.

Complex	0.25 × IC_{50}	0.5 × IC_{50}	1 × IC_{50}	1.5 × IC_{50}
***RR*-1**[a]	7±1	16±1	30±2	53±7
***SS*-2**	7±1	15±1	29±3	48±6
***RR*-2**	7±1	16±1	30±2	52±7

[a] Literature values for the osmium cellular accumulation of ***RR*-1**.[4]

Table S12. Temperature-dependent osmium (^{189}Os) ICP-MS cellular accumulation of in A2780 (human ovarian) cancer cells treated with 1 × IC_{50} of ***SS*-1**, ***SS*-2**, and ***RR*-2** at 277 K or 310 K for 3-6 h. Statistical analysis was performed using the Welch's two-tailed t-test, assuming unequal variances. A statistically significant reduction in osmium accumulation was observed at lower temperatures for all cases.

**Complex**	**3 h**		**6 h**	
	**T = 277 K**	**T = 310 K**	**T = 277 K**	**T = 310 K**
***RR*-1**[a]	3±2	32±4	10.2±0.1	42±1
***SS*-2**	9±1	37±2	20±2	35±3
***RR*-2**	8.9±0.9	35±3	20±3	36±3

[a] Literature values for the osmium cellular accumulation of ***RR*-1**.[4]

Table S13. Formate-dependent osmium (^{189}Os) ICP-MS cellular accumulation in A2780 (human ovarian) cancer cells treated with 1 × IC_{50} (30 µM) of ***SS*-2** or ***RR*-2** upon co-administration with sodium formate (0 or 2 mM). Statistical significance was calculated using a two-tailed t-test assuming unequal variances (Welch's t-test), *$p < 0.05$, **$p < 0.01$ and ***$p < 0.001$. No statistically significant changes in osmium were observed in cells treated with ***SS*-2** or ***RR*-2** in the presence of sodium formate (p=0.7795 and p=1.0000).

**Complex**	**ng Os/ 10^6 cells**
***SS*-2**	29±3
***SS*-2 + Formate**	29.7±2.5
***RR*-2**	31±3
***RR*-2 + Formate**	31±1

ES12 ^{189}Os and ^{79}Br ICP-MS cellular accumulation studies

Optimisation of ICP-MS alkaline method

Prior to analysis of biological samples by ICP-MS, known analyte solutions of osmium and bromine were prepared in 1% *m*/*v* TMAH in triplicate, in addition to known concentrations of compounds containing both osmium and bromine. Solutions of compounds containing bromine, osmium or both were prepared directly in 1% *m/v* TMAH, achieving >95% recoveries. In addition, solutions of compounds containing bromine, osmium or both, and osmium/bromine calibration standards (ICP-grade) were prepared following the alkaline digestion method (e.g. digestion in 500 µL 25% *m*/*v* TMAH followed by 25-fold dilution).

To confirm that ***SS*-2** remains intact in solution, stock solutions of ***SS*-2** were prepared in 1% TMAH in triplicate and diluted to three concentrations in the calibration range 0-1000 ppb (**Figure S14**). All samples achieved >95% recovery. The average molar Br:Os ratio was 1.00 ± 0.03, confirming that the complex has a 1:1 Br:Os ratio prior to cell uptake studies.

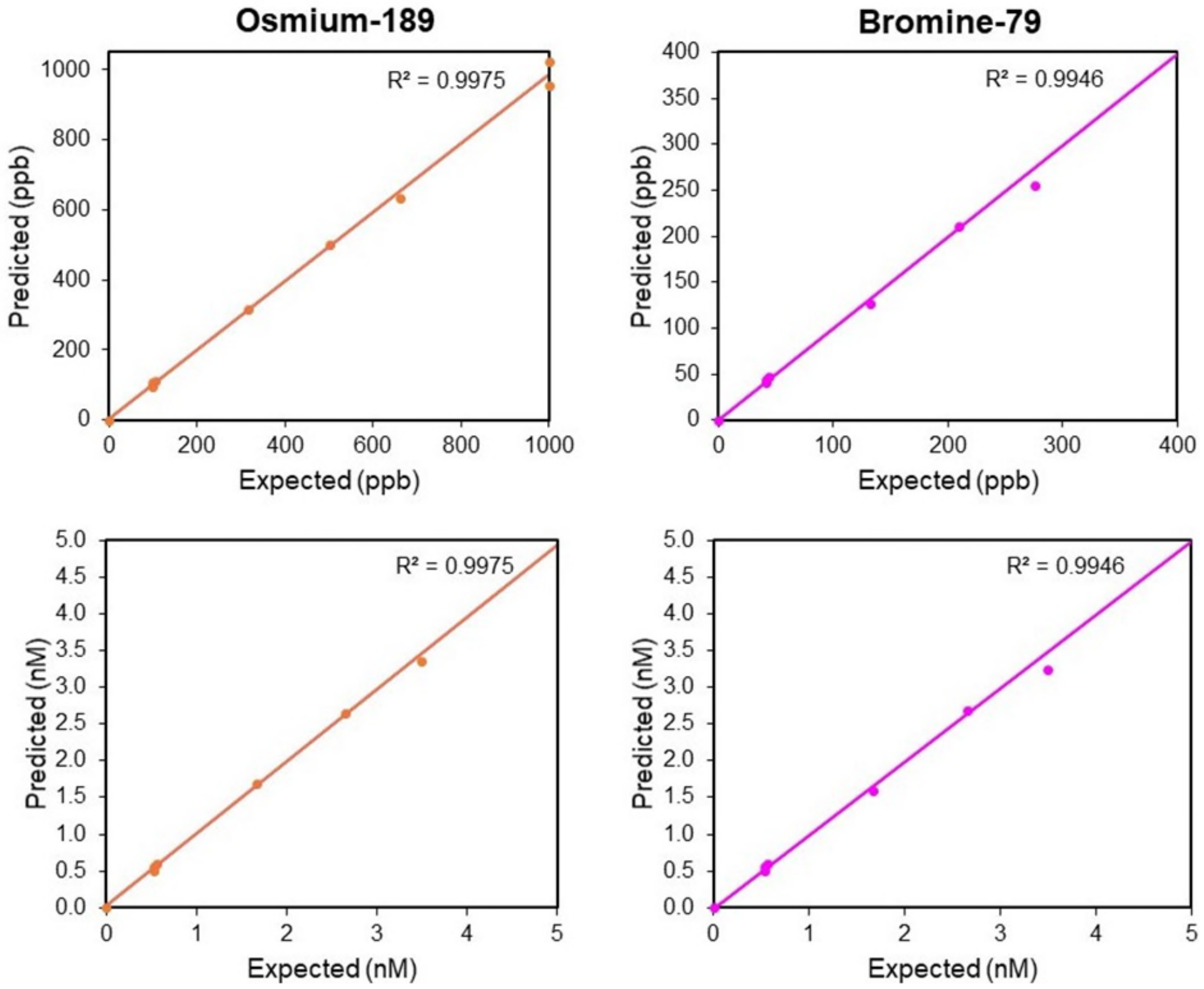

Figure S14. Dilutions of ***SS*-2** prepared in 1% *m/v* TMAH prepared from three separate stocks, and analysed by ICP-MS (1% TMAH in He-gas mode). The molar Br:Os ratio was determined to be 1.00 ± 0.03, confirming that Os and Br-labelled ligand are present at a 1:1 ratio prior to cell accumulation studies.

Alkaline ICP-MS digestion. Cell pellets were digested in 25% *m*/*v* TMAH in aqueous solution (500 µL) at 347 K for 12 h. The samples were diluted in milliQ water to 1% *v*/*v* TMAH for ICP-MS analysis. Dissolved solids did not exceed 1% *w*/*v*. ^{189}Os and ^{79}Br were monitored in He-gas mode using a ^{100}Ru (10 ppb) internal standard and calibrated to 0.1-1000 ppb osmium and bromine standards freshly prepared in 1% *m*/*v* TMAH solution. The limit of detection for ^{189}Os and ^{79}Br were determined to be 1 ppb and 3.7 ppb, respectively.

(i) ***Time-dependent OsBr accumulation.*** The time-dependent osmium cellular accumulation was analysed in A549 cells following the general cellular accumulation protocol. 4 × 10^6 cells were seeded in a 145 mm cell culture Petri dish in 20 mL for 24 h and treated with 30 µM ***SS*-2** in 20 mL and incubated (310 K, 5% CO_2) for different exposure times (3, 6, 18 h, 24 h with no recovery, 24 h exposure with 24, 48 and 72 h recovery in complex-free media). Cells were washed with PBS, detached with 0.25% trypsin/EDTA (2 mL) and single cell suspensions from every two Petri dishes were combined prior to counting and centrifugation as previously described.

(ii) ***Temperature.*** The temperature-dependent ^{189}Os-^{79}Br cellular accumulation was analysed in A549 cells following the general cellular accumulation protocol. Cells were treated with 30 µM concentrations of ***SS*-2** (20 mL) for 3 or 6 h at 277 K or 310 K, respectively. Cells were washed with PBS, detached with 0.25% trypsin/EDTA (2 mL) and single cell suspensions from every two petri dishes were combined prior to counting and centrifugation as previously described.

(iii) ***Inhibition of efflux.*** The verapamil-dependent osmium-bromide cellular accumulation was analysed in A549 cells following the general cellular accumulation protocol. 4 ×10^6 cells were seeded in a 145 mm cell culture Petri dish in 20 mL of DMEM for 24 h. Cells were treated with 30 µM ***SS*-2** (20 mL) for 24 h, and recovered in 20 mL of a 20 µM solution on verapamil hydrochloride (5% *v*/*v* DMSO, 95% *v*/*v* DMEM) for 24, 48 or 72 h. Cells were washed with PBS, detached with 0.25% trypsin/EDTA (2 mL) and single cell suspensions from every two Petri dishes were combined prior to counting and centrifugation as previously described.

(iv) ***Inhibition of caveolae endocytosis.*** The methyl-β-cyclodextrin dependent osmium-bromide cellular accumulation was analysed in A549 cells following the general cellular accumulation protocol. 4 ×10^6 cells were seeded in a 145 mm cell culture Petri dish in 20 mL of media for 24 h. Cells were treated with 30 µM of ***SS*-2** co-administered with 0, 0.1, 0.5 and 1 mM of methyl- β-cyclodextrin (prepared in DMEM) in a total volume of 20 mL for 24 h (310 K, 5% CO_2). Cells were washed with PBS, detached with 0.25% trypsin/EDTA (2 mL) and single cell suspensions from every two Petri dishes were combined prior to counting and centrifugation as previously described.

(v) ***Time-dependent cellular fractionation.*** Cell fractionation studies of A549 cells treated with 30 µM of ***SS*-2** for 3, 6, 18 and 24 h exposure times (no recovery) and 24 h exposure with 24, 48 and 72 h recovery in drug-free media was analysed using FractionPrep BioVision Fractionation Kit, but using twice the specified volumes of protease cocktail mix to account for the larger cell pellet size.

Table S14. Time-dependent osmium (^{189}Os) and bromine (^{79}Br) ICP-MS cellular accumulation in A549 (human lung) cancer cells treated with 30 µM of ***SS*-2** or ***SS*-L1** for different exposure times (3, 6, 24 h) and 24 h with different recovery times in drug-free media (24, 48 and 72 h).

ng / 10^6 cells				
	***SS*-L1**	***SS*-2**		
**Time / h**	**Bromine**	**Osmium**	**Bromine**	**Molar Br / Os**
3	-	46±3	181±20	9.4±1.2
6	-	46± 9	244±39	13±3
24	336±13	46±3	566±11	30±2
24 + 24[a]	-	9.1±2.4	270±54	71±23
24 + 48[a]	-	3.9±0.8	171±31	105±29
24 + 72[a]	-	3.0±0.9	146±44	116±50

[a] 24 h exposure, followed by 24, 48 or 72 h recovery in complex-free media.

Table S15. Time-dependent osmium (^{189}Os) fractional ICP-MS cellular accumulation in A549 (human lung) cancer cells treated with 1 × IC_{50} (30 µM) of ***SS*-2** for different exposure times showing the cytosolic, membrane, nuclear and cytoskeletal fractions. Osmium was predominantly distributed in the membrane fraction (>65% in all cases), but also present to a small extent in the cytoskeletal fraction (>6%). Importantly, the majority of Os was not in the nuclear fraction.

	Average % intracellular osmium			
**Time / h**	**Cytosolic**	**Membrane**	**Nuclear**	**Cytoskeletal**
3	3.4±0.6	84±12	5.7±0.5	7.1±1.4
6	1.7±0.8	86±9	6.0±0.3	6.2±0.5
18	2.7±0.2	83±6	6.2±1.1	8.0±0.4
24	5.6±2.8	81±9	5.5±0.4	8.2±1.4
24+24	1.4±0.1	80±5.2	5.5±0.3	12.9±0.9
24+48	1.6±0.2	84±18	4.6±1.6	9.4±2.1
24+72	4.7±2.9	66±9	9.2±2.7	19.6±6.7

Table S16. Time-dependent bromine (^{79}Br) fractional ICP-MS cellular accumulation in A549 (human lung) cancer cells treated with 1 × IC_{50} (30 µM) of ***SS*-2** for different exposure times showing the cytosolic, membrane, nuclear and cytoskeletal fractions. The majority of Br for all exposure times was distributed in the membrane fraction, however, unlike Os, Br was also distributed in the nuclear fraction >10% for all time points which may be suggestive of the BrPh-DPEN ligand entering the cell nucleus.

	Average % intracellular bromine			
**Time / h**	**Cytosolic**	**Membrane**	**Nuclear**	**Cytoskeletal**
3	17.2±1.4	58.1±7.4	19.7±1.2	4.9±2.1
6	8.9±6.1	72.9±6.9	15.3±0.4	2.9±0.2
18	6.2±0.1	80.7±5.9	10.0±0.6	3.1±0.3
24	10.6±2.6	74.9±9.8	11.8±0.8	2.7±0.004
24+24	6.5±0.2	79.5±3.5	11.2±0.3	2.8±0.2
24+48	7.1±0.8	77.6±16.2	11.1±1.4	4.2±0.9
24+72	10.3±2.3	64.5±0.65	17.6±1.4	7.5±1.8

Table S17. Normalized fractional moles of osmium (^{189}Os) and bromine (^{79}Br) in the nuclear fraction in A549 (human lung) cancer cells treated with 1 × IC_{50} (30 µM) of ***SS*-2** for different exposure times: osmium and bromine, as reported in fractional nmol ×10^{-8} per cell. Intracellular Br was found to be distributed in the nuclear fraction (*ca.* 10%) for all exposure times, in contrast to Os (<5%).

	Fraction nmol × 10^{-8} per cell	
**Time / h**	**Osmium**	**Bromine**
**3**	1.4±0.4	46.4±15.5
**6**	1.5±0.6	48±19
**24**	1.3±0.3	85±13
**24+24**	0.3±0.1	39±17
**24+48**	0.10±0.05	25±11
**24+72**	0.13±0.07	28± 8

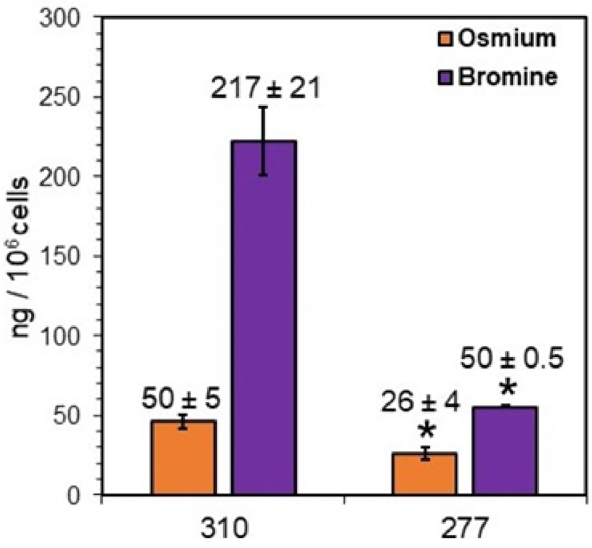

Figure S15. Temperature-dependent net accumulation of osmium (^{189}Os, orange) and bromine (^{79}Br, purple) in A549 (human lung) cancer cells treated with 1 × IC_{50} (30 µM) of ***SS*-2** at 310 K and 277 K for 6 h (no recovery). Statistical analysis was performed using the Welch's two-tailed t-test, assuming unequal variances, *$p < 0.05$, **$p < 0.01$ and ***$p < 0.001$. Significantly lower levels of both osmium (p=0.0074) and bromine (p=0.0052) at lower temperatures.

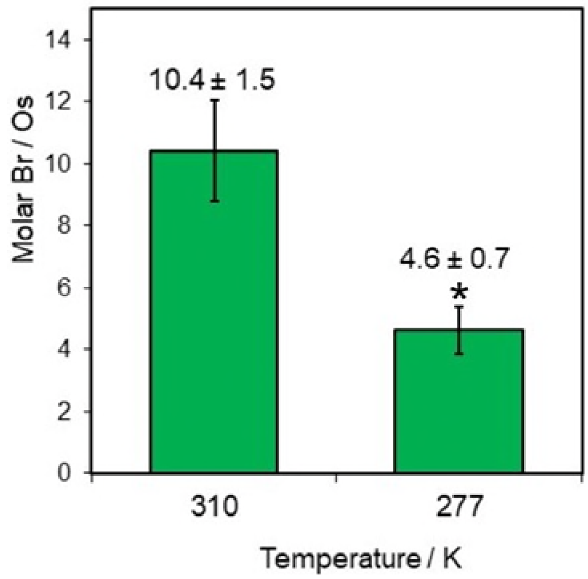

Figure S16. Temperature-dependent Br / Os molar ratios in A549 (human lung) cancer cells treated with 1 × IC_{50} (30 µM) of ***SS*-2** at 310 and 277 K for 6 h (no recovery). Statistical analysis was performed using the Welch's two-tailed t-test, assuming unequal variances, **p* < 0.05, ***p* < 0.01 and ****p* < 0.001. A significant decrease in the molar bromine-to-osmium (Br / Os) was observed at lower temperatures (*p*=0.0261).

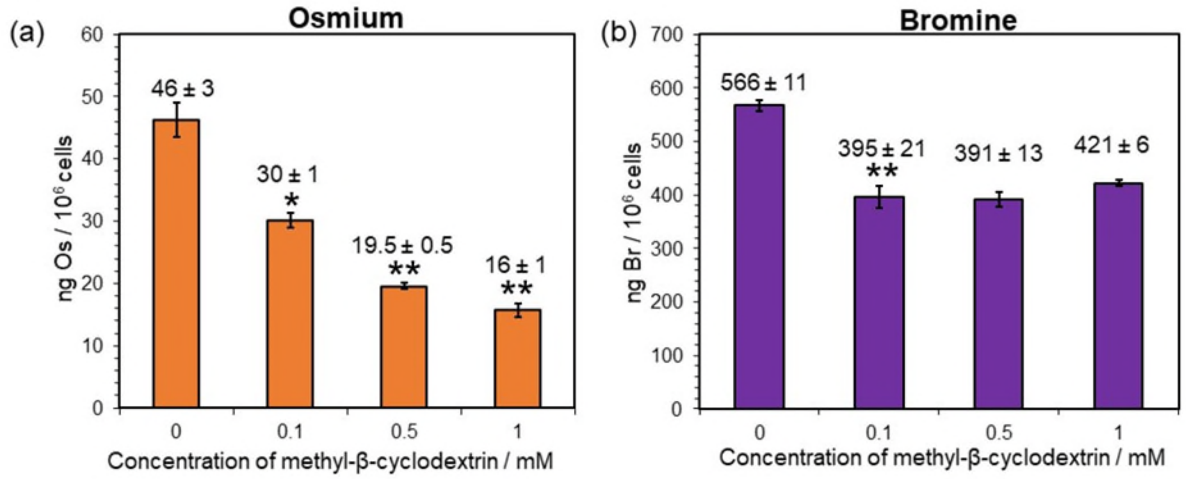

Figure S17. The effect of methyl-β-cyclodextrin (endocytotic inhibitor) co-administration on the amount of osmium (^{189}Os, **orange**) and bromine (^{79}Br, **purple**) in A549 (human lung) cancer cells treated with 1 × IC_{50} (30 µM) of ***SS*-2** for 24 h: (a) Os ; (b) Br. Statistical analysis was performed using Welch's two-tailed t-test, assuming unequal variances, **p* < 0.05, ***p* < 0.01 and ****p* < 0.001. Statistically significant decrease in osmium was observed upon increasing concentration of methyl-β-cyclodextrin when compared to the controls: *p*=0.0128 (0.1 mM *vs* controls), *p*=0.0038 (0.1 mM *vs.* 0.5 mM) and *p*=0.0324 (0.5 mM *vs.* 1 mM). In contrast, a significant decrease in Br accumulation was initially observed upon co-administration with the lowest concentration of methyl-β-cyclodextrin (0.1 mM) compared to the untreated controls (*p*=0.0011), but was independent on the inhibitor concentration. This may suggest that methyl-β-cyclodextrin reduces overall complex uptake, but, once in the cell, more of the Br-labelled ligand remains inside the cell.

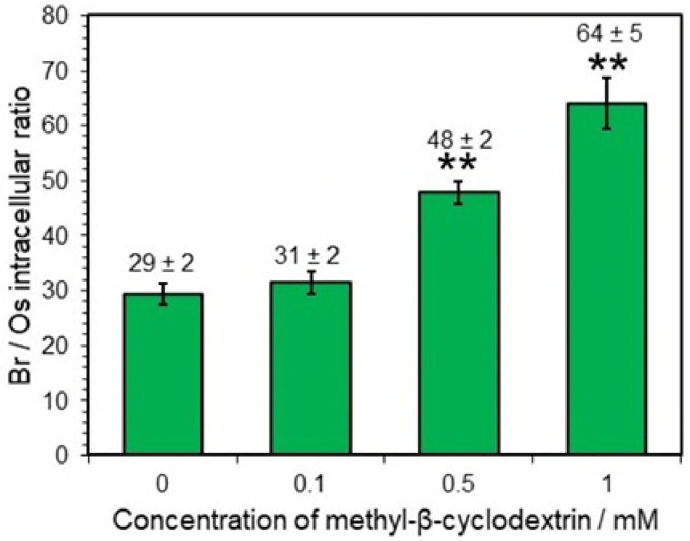

Figure S18. Intracellular Br / Os molar ratios determined for A549 cells treated with 1 × IC_{50} (30 µM) of ***SS-2*** upon co-administration with methyl-β-cyclodextrin (0.1-1 mM) for 24 h. Statistical analysis was performed using the Welch's two-tailed t-test, assuming unequal variables, *$p < 0.05$, **$p < 0.01$ and ***$p < 0.001$. No statistically significant different in molar Br / Os was observed between the cells treated with ***SS*-2** (positive controls) and cells co-administered with ***SS*-2** and 0.1 mM methyl-β-cyclodextrin (p>0.05), however, a statistically significant increase in Br / Os ratio was observed upon co-administration with 0.5-1 mM methyl-β-cyclodextrin when compared to the positive controls (p=0.0046 and p=0.007, respectively. This suggests that SS-2 enters cells via endocytosis, but once in the cell is rapidly degraded, with more of the Br-labelled ligand remains inside the cell.

Table S18. Verapamil-dependent efflux of osmium (^{189}Os) and bromine (^{79}Br) in A549 (human lung) cancer cells treated with 1 × IC_{50} (30 µM) of ***SS*-2** for 24 h, and recovery in 0-20 µM of verapamil hydrochloride (prepared in 5% *v/v* DMSO, 95% *v/v* DMEM) for 24-72 h. Statistical significance was calculated using a two-tailed t-test assuming unequal variances (Welch's t-test) and comparing to the untreated controls, *$p < 0.05$, **$p < 0.01$ and ***$p < 0.001$.

	**Complex-free media**		**+ Verapamil**	
**Recovery time / h**	**Os ng / 10^6 cells**	**Br ng / 10^6 cells**	**Os ng / 10^6 cells**	**Br ng / 10^6 cells**
**24**	9.1±2.4	270±54	11.5±2.4	238±35
**48**	3.9±0.8	171±31	6.6±0.06	173±20
**72**	3.0±0.9	146±44	5.7±2.3	178±64

Table S19. Intracellular molar ratio (Br / Os in A549 (human lung) cancer cells treated with 1×IC_{50} (30 µM) of ***SS*-2** for 24 h, and recovered in 0-20 µM of verapamil hydrochloride (prepared in 5% *v/v* DMSO, 95% *v/v* DMEM) for 24-72 h. Statistical significance was calculated using a two-tailed t-test assuming unequal variances (Welch's t-test) and comparing to the untreated controls, *$p < 0.05$, **$p < 0.01$ and ***$p < 0.001$.

	**Br / Os molar ratio**	
**Recovery time / h**	**Complex-free media**	**+ Verapamil**
24	71±23	50±13
48	105±29	63±7
72	116±50	75±40

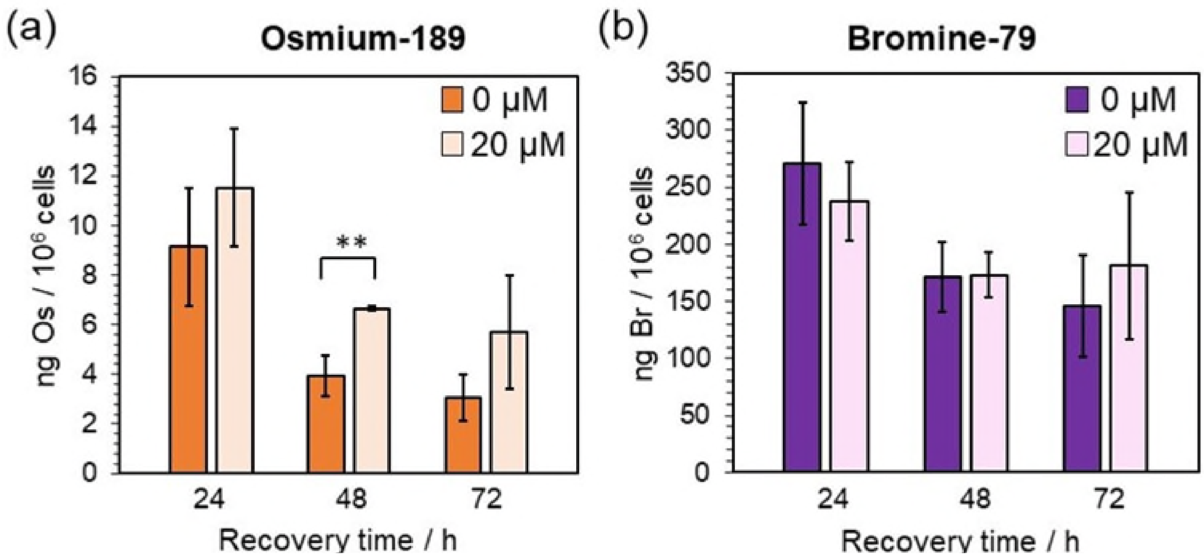

Figure S19. Effect of verapamil on the amount of of osmium and bromine in A549 (human lung) cancer cells treated with 1 × IC_{50} (30 µM) of ***SS*-2** for 24 h, followed by recovery in 0-20 µM verapamil for 24-72 h: (a) ^{189}Os (metal center - **orange**); (b) ^{79}Br (ligand - **purple**). Statistical analysis was performed using Welch's two-tailed t-test, assuming unequal variances, *$p < 0.05$, **$p < 0.01$ and ***$p < 0.001$. A significant reduction in Os accumulation was observed upon 24 h exposure followed by 48 h recovery in verapamil when compared to treated cells recovered in drug-free medium (p=0.0282). This suggests that Os efflux is dependent on PGP. No statistically significant differences in intracellular Br were observed upon recovery in verapamil (p>0.05).

ES13 Zebrafish embryo toxicity

The University of Warwick is a member of the Laboratory Animal Science Association and the Institute of Animal Technology.[7] All *in vivo* animal work was approved by the University Ethical Review Committee.[7] Zebrafish experiments were carried out at the School of Life Sciences (University of Warwick) under project AWERB.10/16-17, with the advice of Dr. Karuna Sampath and Ian Bagley (BSU Research Technology Platform, Warwick).[7]

12-well plates were seeded with a minimum a single SG-WT zebrafish embryo per well and treated with different concentrations of ***SS*-2** or ***RR*-2** (0.01-50 µM) and incubated at 301.5 K for 96 h. The mortality of the embryos was analysed by observing (a) tail detachment from body, (b) somite formation, (c) coagulation of embryo and (d) heartbeat. Sigmoidal dose-response curves were plotted by taking the logarithm of osmium complex concentration against the percentage survival to determine half-lethal inhibitory concentrations (LC_{50} / µM; **Table S20**).

Table S20. Half-maximal lethal concentrations (LC_{50} / µM) for ***RR*-1**, ***SS*-2**, ***RR*-2**, ***RR*-4** and cisplatin in SG-WT zebrafish embryos (96 h drug exposure, no recovery period).

Complex	R	LC_{50} (µM)
***RR*-1**[a]	*p*-Me	2.4±0.4
***SS*-2**	*p*-Br	3.7±0.3
***RR*-2**	*p*-Br	4.1±0.1
***RR*-4**[a]	*p*-F	0.7±0.2
**Cisplatin**[a]	-	0.6± 0.2

[a]Literature LC_{50} values of ***RR*-1** (R=*p*-CH_3), ***RR*-4** (R=*p*-F) and **cisplatin**.[7]

ES14 Synchrotron-XRF

Preparation of Tris-glucose buffer. Tris-glucose buffer was prepared by mixing D-glucose (11.756 g, 0.065 mol), tris(hydroxymethyl)aminomethane (0.307 g, 2.53 mmol) in 9 mM acetic acid (12.9 mL) and made up to 250 mL in milliQ pure water. The resulting solution had pH = 7.42. The buffer was then sterile filtered using a 500 mL Millipore Stericup filter unit (Durapore PVDF membrane, low protein-binding, pore size 0.22 µm).

Preparation of A549 cells on Si_3N_4 membranes. Silicon nitride TEM grids (frame size = 5 × 5 mm, frame thickness = 200 µm, membrane size 1.5 ×1.5 mm, membrane thickness 500 nm) were placed into a 6-well plate (1 per well) with the flat side down. The grids were then washed in 70% ethanol (3 mL / well), followed by 100% ethanol (3 mL / well). The ethanol was removed by suction and the grids left to air dry at room temperature in a sterile environment. Then, 2-3 drops of 0.01% poly-L-lysine were added directly to each membrane for 20 min. The grids were washed with PBS (2 × 3 mL). The PBS was removed and left to air-dry at room temperature. Then, 50 µL of a 60,000 cells / mL suspension of A2780 human ovarian cancer cells was added directly to each membrane and left to incubate for 2 h (310 K, 5% CO_2). 3 mL of the *same* 60,000 cells/mL cell suspension was added to each well and incubated for 24 h (37 C, 5% CO_2). After 24 h incubated, the media was removed using a suction pump and 3 mL of solutions of ***SS*-2** (30, 90 and 150 µM, prepared in 5% *v*/*v* DMSO, 95% *v*/*v* DMEM culture medium) were added to each well for 24 h (310 K, 5% CO_2). The DMEM medium was removed and the grids were washed with tirs-glucose buffer (2 × 3 mL). The Tris-glucose buffer was removed immediately before rinsing in sterile water, blotting with filter paper and plunge-freezing in liquid propane-ethane mixture (cooled in liquid nitrogen). The grids were then transferred to sterile cryo-vials in liquid nitrogen, then each vial was covered in parafilm pierced with a needle and placed in freeze-dryer for 24-48 h.

XRF elemental map acquisition at I14 Beamline. Si_3N_4 membranes (1.5 × 1.5 mm membrane size) were loaded into the sample holder available at I14 using fine tweezers, with the flat side on the surface. The grids were individually screwed into the sample holders, and, once secure, was loaded vertically onto the beamline (with the screw-side closest to the incoming x-ray beam). An incident energy beam of 15 keV was used, focussing the hard x-ray beam size of 100 nm. The flux was calculated by calibrating to an AXO standard (with known concentrations of Pd, Ca, La, Fe, Cu, Pb and Mo). Synchrotron-XRF elemental maps were obtained by raster-scanning using different step sizes and 100 nm Xspress Malcom detector. Coarse scans were first run to confirm the position of cells (500 nm step size, 0.1 s), before fine scans (100 nm step size, 0.1 s) were used to analyse elemental composition with higher resolution.

PyMca data analysis. The processed data obtained from the synchrotron-XRF experiment were analysed using PyMca X-ray Fluorescence toolkit developed by the software group at the ESRF. The flux was calculated by calibrating to an AXO standard (with known concentrations of Pd, Ca, La, Fe, Cu, Pb and Mo) and assuming a density of 0.0012 g/cm^3 and maximal cell thickness of 6 µm.[9] A configuration file for the AXO Si_3N_4 reference material was prepared in PyMCa. Once normalized, the input data .nxs files were selected, in addition to the prepared configuration file (with the specified flux) and all data were fit to the same configuration file. Once complete, .edf files were generated and visualized in fiji image-J win-64 using EdfRead plugin.

ImageJ data analysis. Analysis of elemental-XRF maps was performed using ImageJ (fiji64) software with 10 pixels = 1 µm. The area and roundness factors of each individual A549 cell

(and regions of interest) was measured in triplicate using the "multipoint" tool. The co-localization between elements in ROI was measured using the Coloc2 plugin and the statistical output Pearson R-value, Spearman Rank Correlation and Costes P-value have been reported. The quantification elemental analysis was performed in triplicate using the mass fraction output data file (calibrated to the AXO standard) and selecting ROI using the multipoint tool.

XRF Elemental Maps

XRF maps of cryo-fixed and freeze-dried A549 human lung cancer cells treated with 1-3 × IC_{50} of SS-2 for 24 h were generated at the I14 beamline (DLS) using a100×100 nm^2 step size, 0.1 s dwell time. Data were fitted in PyMCa toolkit (**Figure S19**). A total of 18 cells were mapped by XRF: (i) Untreated (**Figures S21-24**), (ii) 1 × IC_{50} (**Figures S25-28**), (iii) 3 × IC_{50} (**Figures S29-35**), (iv) 5 × IC_{50} (**Figures S36-38**).

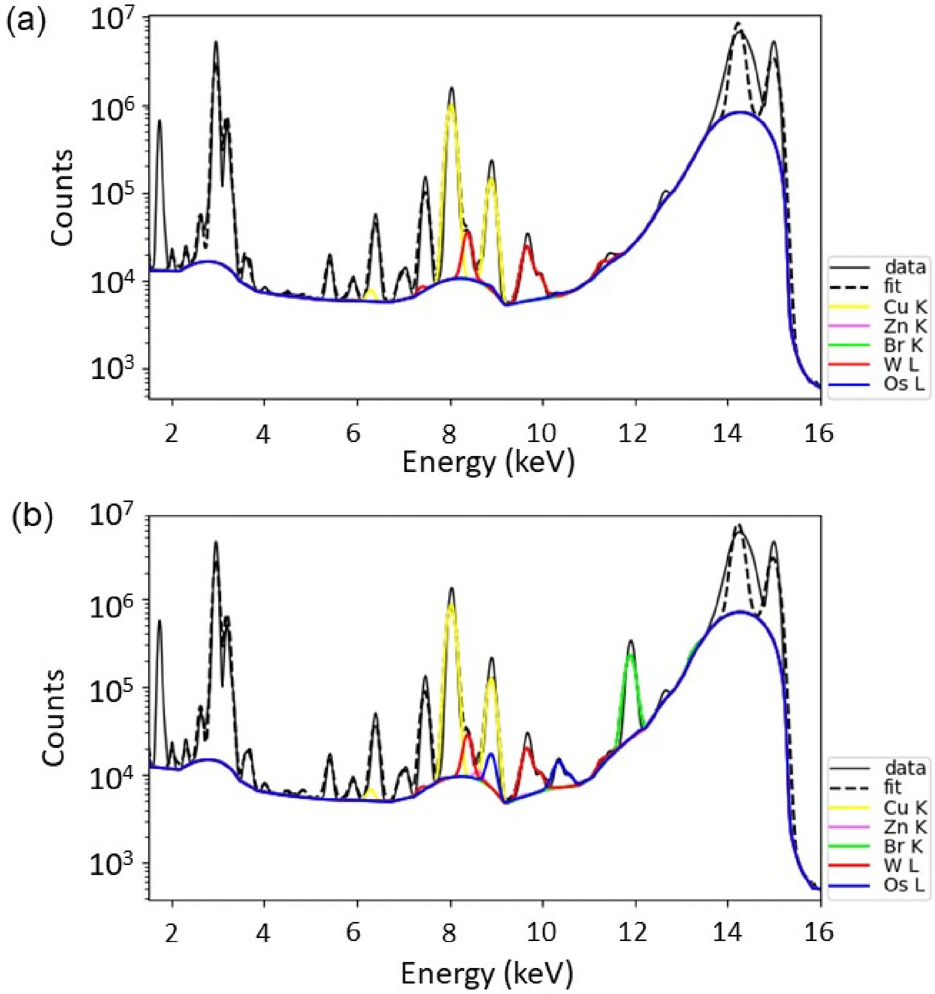

Figure S20. Representative XRF spectra for a cryo-fixed and freeze-dried A549 (human lung) cancer cell; raster scan: 100×100 nm^2 step size, 0.1 s dwell time as obtained by synchrotron-XRF. Data were fitted in PyMCa toolkit (ESRF).[10] The contribution of Cu K (**yellow**), Zn K (**pink**), Br K (**green**), W L (**red**) and Os L (**blue**) emission lines are shown. (a) Untreated control. (b) Cell treated with 1 × IC_{50} (30 µM) of ***SS*-2** for 24 h (no recovery in drug-free media).

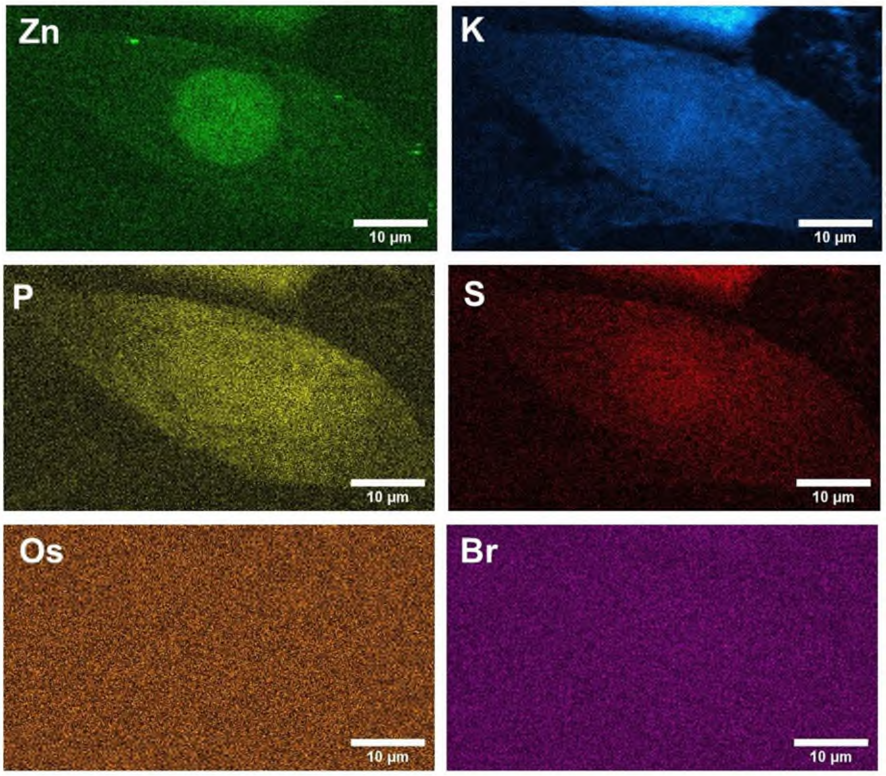

Figure S21. Synchrotron XRF elemental map of a cryo-fixed and freeze-dried A549 human lung cancer cell (**C1**): (i) Zn (**green**); (ii) K (**blue**); (iii) P (**yellow**); (iv) S (**red**); (v) Os (**orange**); (vi) Br (**pink**). Maps were collected using 100 nm step size and 0.1 s dwell time. Data were processed in PyMca toolkit developed by the ESRF,[10] and images generated in ImageJ.[11]

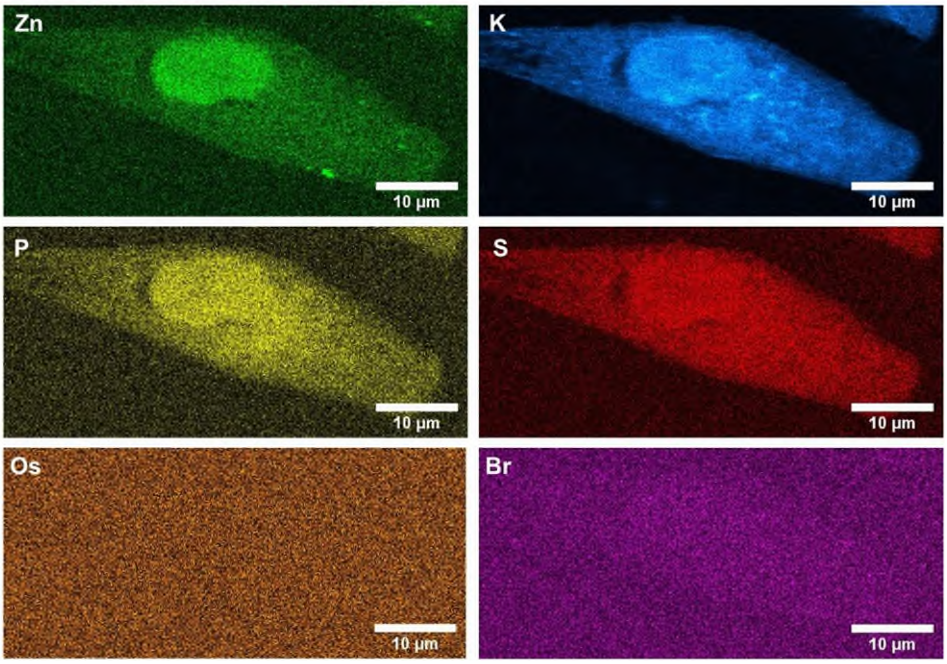

Figure S22. Synchrotron XRF elemental map of a cryo-fixed and freeze-dried A549 cell (**C2**): (i) Zn (**green**); (ii) K (**blue**); (iii) P (**yellow**); (iv) S (**red**); (v) Os (**orange**); (vi) Br (**pink**). Maps were collected using 100 nm step size and 0.1 s dwell time. Data were processed in PyMca toolkit developed by the ESRF,[10] and images generated in ImageJ.[11]

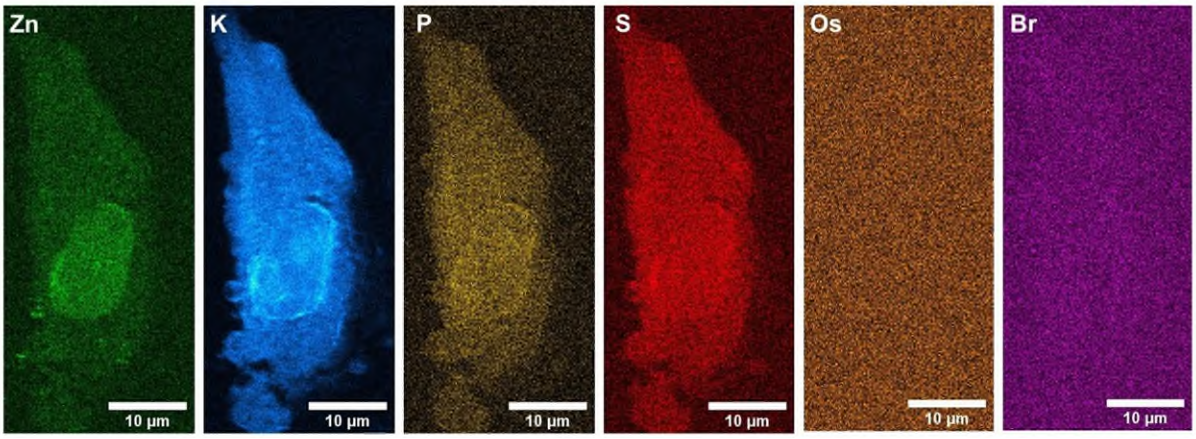

Figure S23. Synchrotron XRF elemental map of a cryo-fixed and freeze-dried A549 cell (**C3**): (i) Zn (**green**); (ii) K (**blue**); (iii) P (**yellow**); (iv) S (**red**); (v) Os (**orange**); (vi) Br (**pink**). Maps were collected using 100 nm step size and 0.1 s dwell time. Data were processed in PyMca toolkit developed by the ESRF,[10] and images generated in ImageJ.[11]

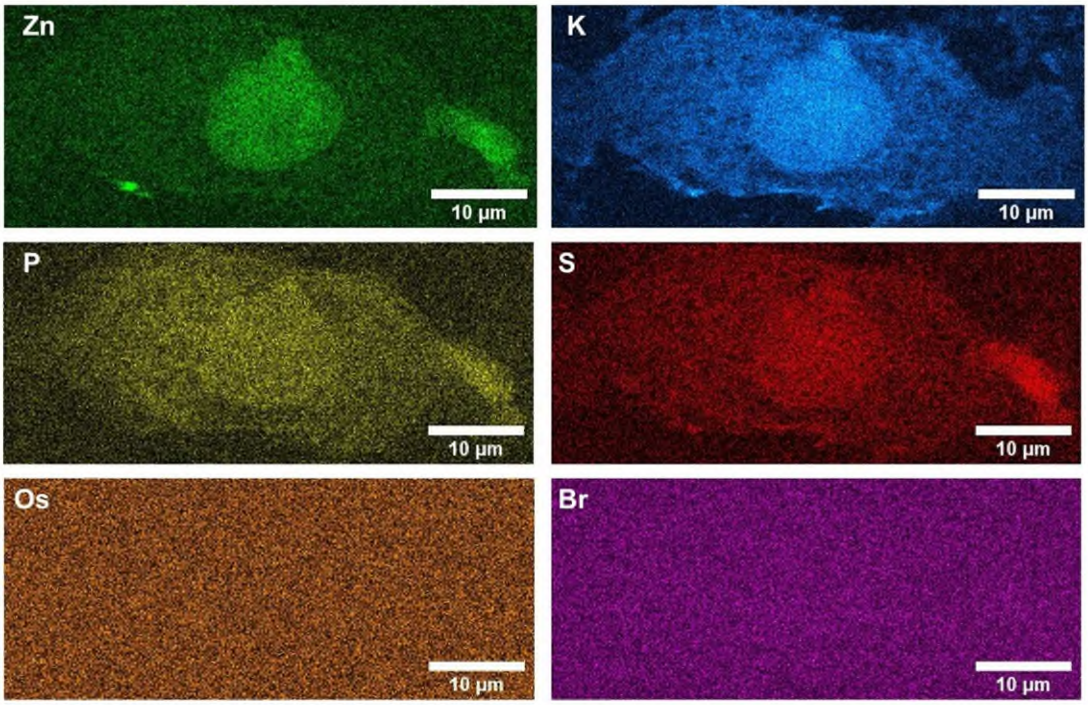

Figure S24. Synchrotron XRF elemental map of a cryo-fixed and freeze-dried A549 cell (**C4**): (i) Zn (**green**); (ii) K (**blue**); (iii) P (**yellow**); (iv) S (**red**); (v) Os (**orange**); (vi) Br (**pink**). Maps were collected using 100 nm step size and 0.1 s dwell time. Data were processed in PyMca toolkit developed by the ESRF,[10] and images generated in ImageJ.[11]

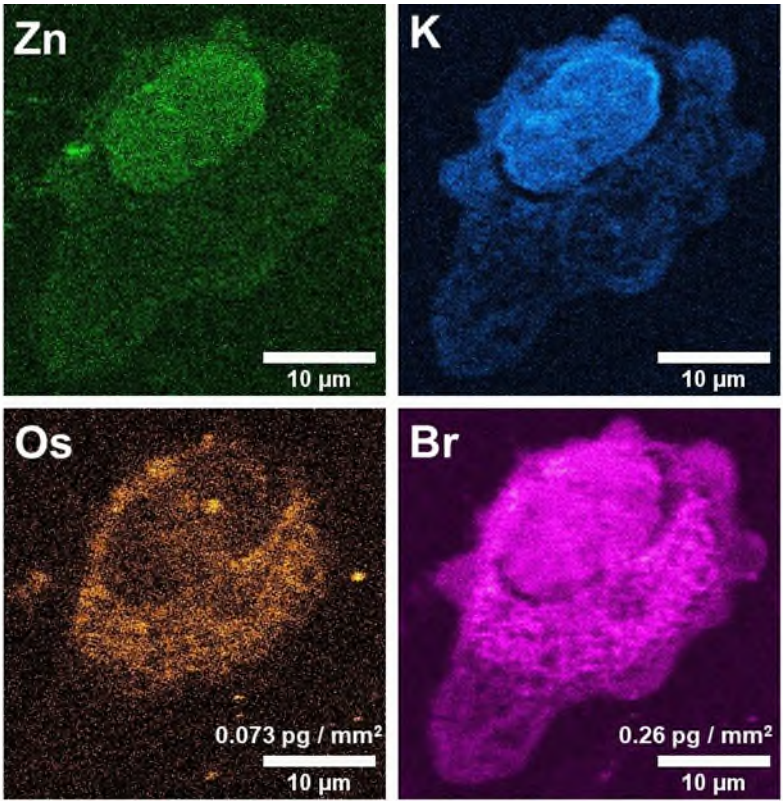

Figure S25. Synchrotron XRF elemental map of a cryo-fixed and freeze-dried A549 cell treated with 1 × IC_{50} (30 μM) of ***SS*-2** for 24 h (**C5**): (i) Zn (**green**); (ii) K (**blue**); (iii) Os (**orange**); (iv) Br (**pink**). Maps were collected using 100 nm step size and 0.1 s dwell time. Data were processed in PyMca toolkit developed by the ESRF,[10] and images generated in ImageJ.[11] Quantities of Os and Br are reported in pg mm^{-2}. Osmium and bromine co-localize in the cell cytoplasm. However, bromine is also strongly co-localized with zinc in the cell nucleus.

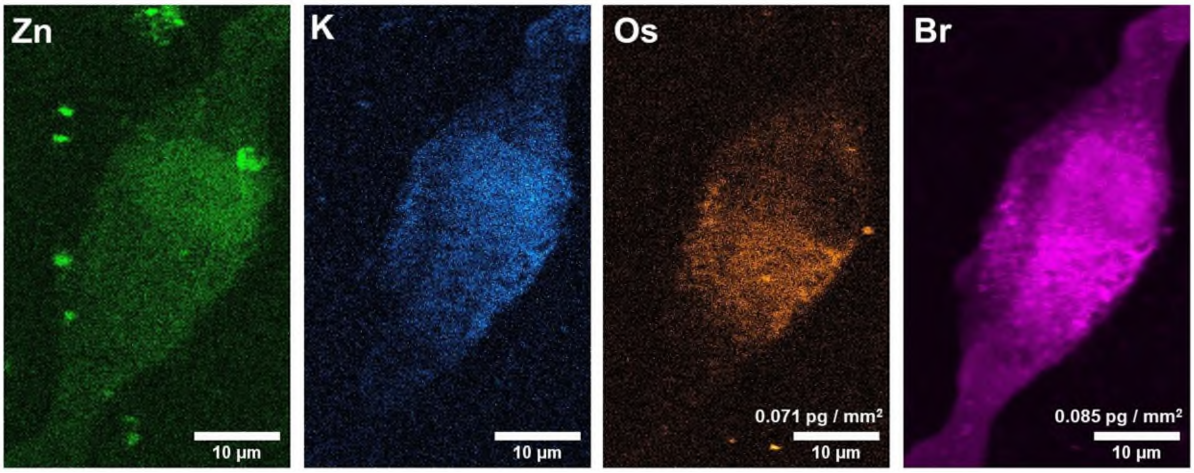

Figure S26. Synchrotron XRF elemental map of a cryo-fixed and freeze-dried A549 cell treated with 1 × IC_{50} (30 μM) of ***SS*-2** for 24 h (**C6**): (i) Zn (**green**); (ii) K (**blue**); (iii) Os (**orange**); (iv) Br (**pink**). Maps were collected using 100 nm step size and 0.1 s dwell time. Data were processed in PyMca toolkit developed by the ESRF,[10] and images generated in ImageJ.[11] Quantities of Os and Br are reported in pg mm^{-2}. Os and Br co-localize in the cell cytoplasm. However, Br is also strongly co-localized with Zn in the cell nucleus.

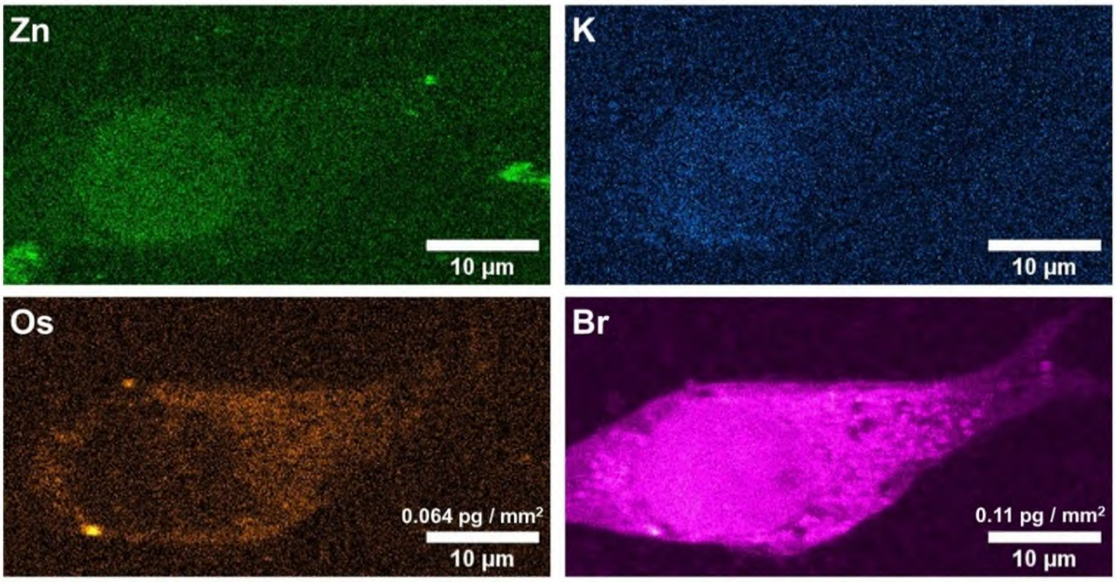

Figure S27. Synchrotron XRF elemental map of a cryo-fixed and freeze-dried A549 cell treated with 1 × IC_{50} (30 µM) of ***SS*-2** for 24 h (**C7**): (i) Zn (**green**); (ii) K (**blue**); (iii) Os (**orange**); (iv) Br (**pink**). Maps were collected using 100 nm step size and 0.1 s dwell time. Data were processed in PyMca toolkit developed by the ESRF,[10] and images generated in ImageJ.[11] Quantities of Os and Br are reported in pg mm^{-2}. Os and Br co-localize in the cell cytoplasm. However, Br is also strongly co-localized with Zn in the cell nucleus.

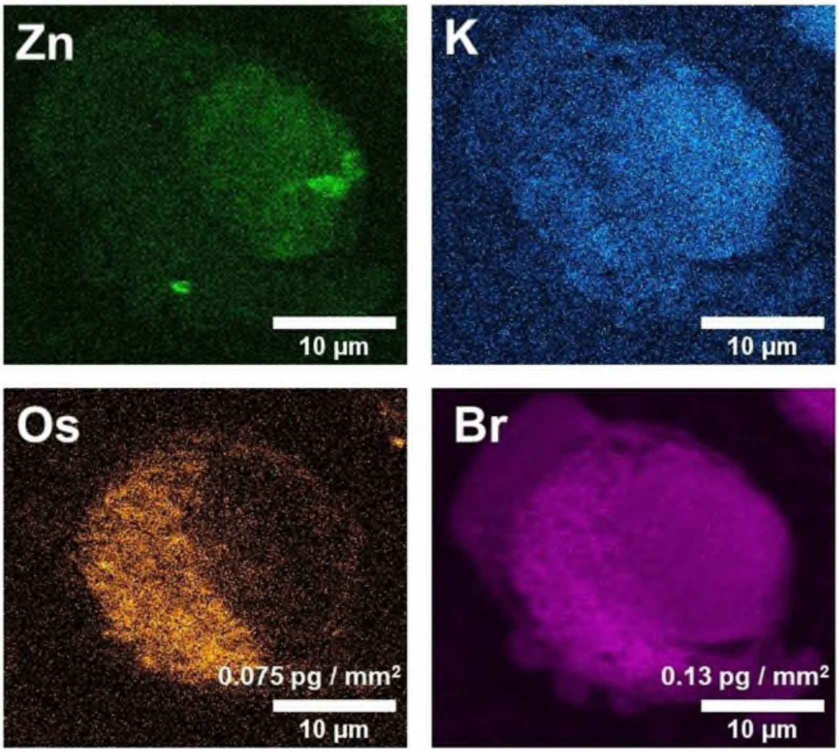

Figure S28. Synchrotron XRF elemental map of a cryo-fixed and freeze-dried A549 cell treated with 1 × IC_{50} (30 µM) of ***SS*-2** for 24 h (**C8**): (i) Zn (**green**); (ii) K (**blue**); (iii) Os (**orange**); (iv) Br (**pink**). Maps were collected using 100 nm step size and 0.1 s dwell time. Data were processed in PyMca toolkit developed by the ESRF,[10] and images generated in ImageJ.[11] Quantities of Os and Br are reported in pg mm^{-2}. Os and Br co-localize in the cell cytoplasm. However, Br is also strongly co-localized with Zn in the cell nucleus.

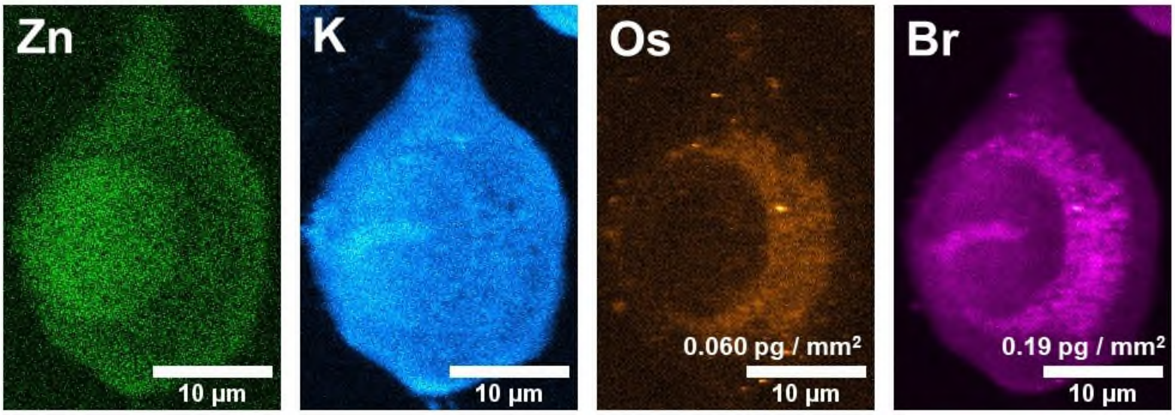

Figure S29. Synchrotron XRF elemental map of a cryo-fixed and freeze-dried A549 cell treated with 3 × IC_{50} (90 µM) of ***SS*-2** for 24 h (**C9**): (i) Zn (**green**); (ii) K (**blue**); (iii) Os (**orange**); (iv) Br (**pink**). Maps were collected using 100 nm step size and 0.1 s dwell time. Data were processed in PyMca toolkit developed by the ESRF,[10] and images generated in ImageJ.[11] Quantities of Os and Br are reported in pg mm^{-2}. Os and Br co-localize in the cell cytoplasm. However, Br is also strongly co-localized with Zn in the cell nucleus.

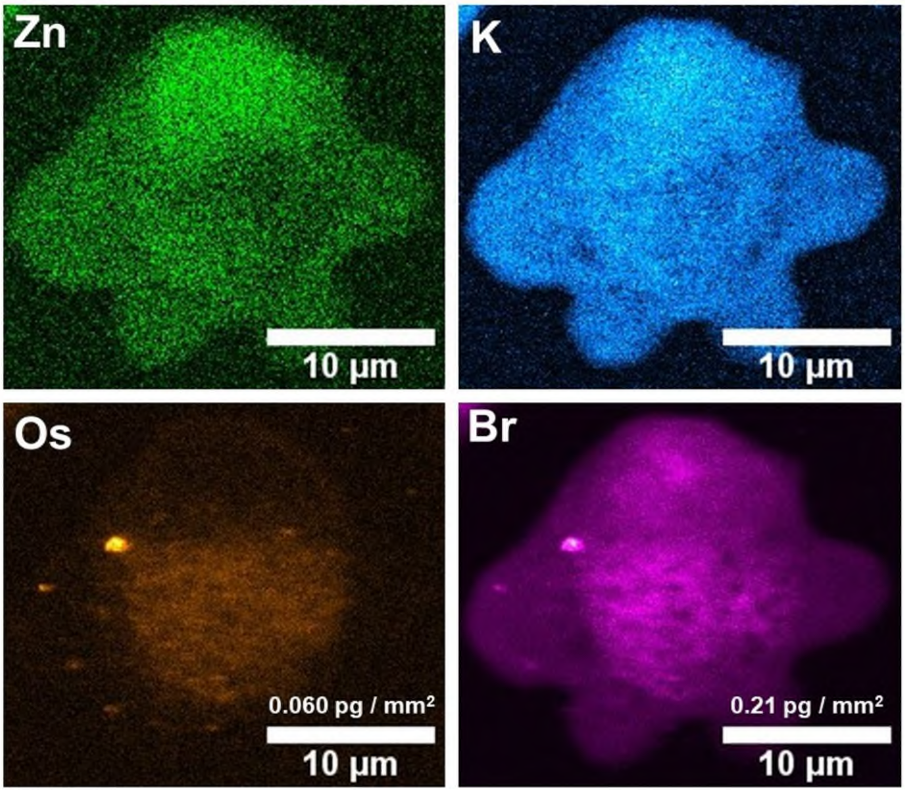

Figure S30. Synchrotron XRF elemental map of a cryo-fixed and freeze-dried A549 cell treated with 3 × IC_{50} (90 µM) of ***SS*-2** for 24 h (**C10**): (i) Zn (**green**); (ii) K (**blue**); (iii) Os (**orange**); (iv) Br (**pink**). Maps were collected using 100 nm step size and 0.1 s dwell time. Data were processed in PyMca toolkit developed by the ESRF,[10] and images generated in ImageJ.[11] Quantities of Os and Br are reported in pg mm^{-2}. Os and Br co-localize in the cell cytoplasm. However, Br is also strongly co-localized with Zn in the cell nucleus.

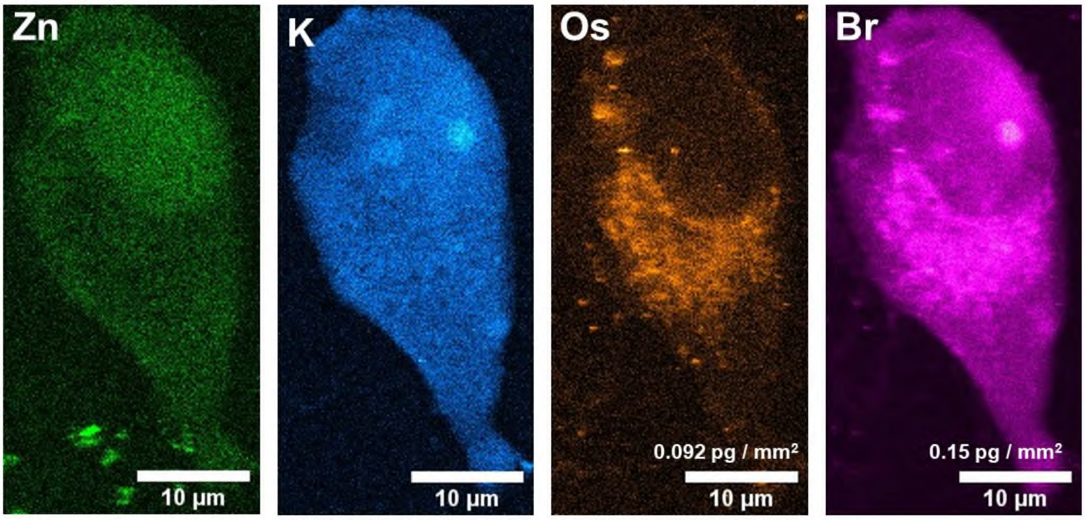

Figure S31. Synchrotron XRF elemental map of a cryo-fixed and freeze-dried A549 cell treated with 3 × IC_{50} (90 µM) of ***SS*-2** for 24 h (**C11**): (i) Zn (**green**); (ii) K (**blue**); (iii) Os (**orange**); (iv) Br (**pink**). Maps were collected using 100 nm step size and 0.1 s dwell time. Data were processed in PyMca toolkit developed by the ESRF,[10] and images generated in ImageJ.[11] Quantities of Os and Br are reported in pg mm^{-2}. Os and Br co-localize in the cell cytoplasm. However, bromine is also strongly co-localized with Zn in the cell nucleus.

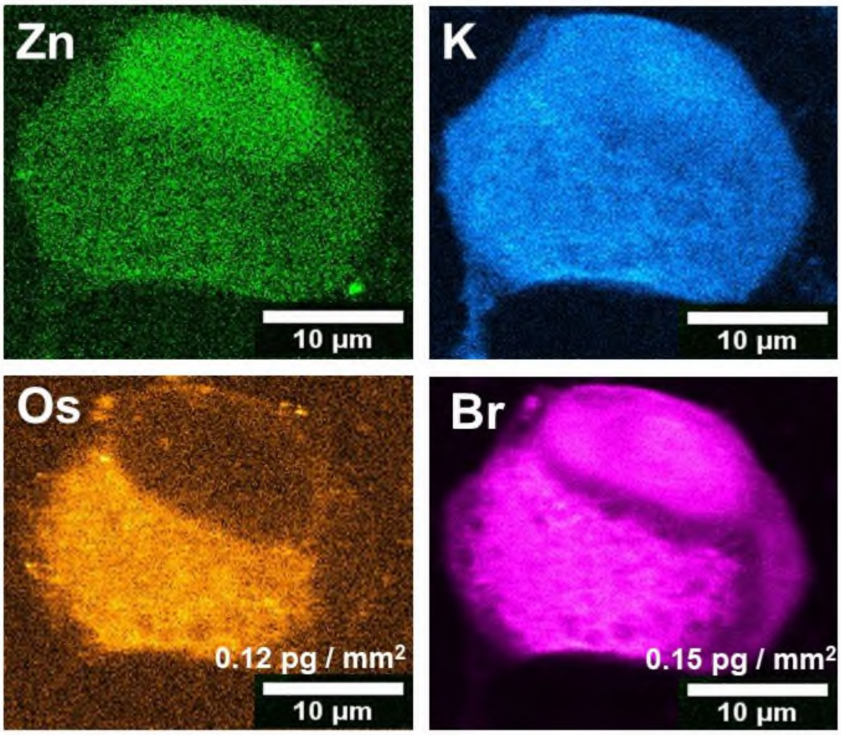

Figure S32. Synchrotron XRF elemental map of a cryo-fixed and freeze-dried A549 cell treated with 3 × IC_{50} (90 µM) of ***SS*-2** for 24 h (**C12**): (i) Zn (**green**); (ii) K (**blue**); (iii) Os (**orange**); (iv) Br (**pink**). Maps were collected using 100 nm step size and 0.1 s dwell time. Data were processed in PyMca toolkit developed by the ESRF,[10] and images generated in ImageJ.[11] Quantities of Os and Br are reported in pg mm^{-2}. Os and Br co-localize in the cell cytoplasm. However, Br is also strongly co-localized with Zn in the cell nucleus.

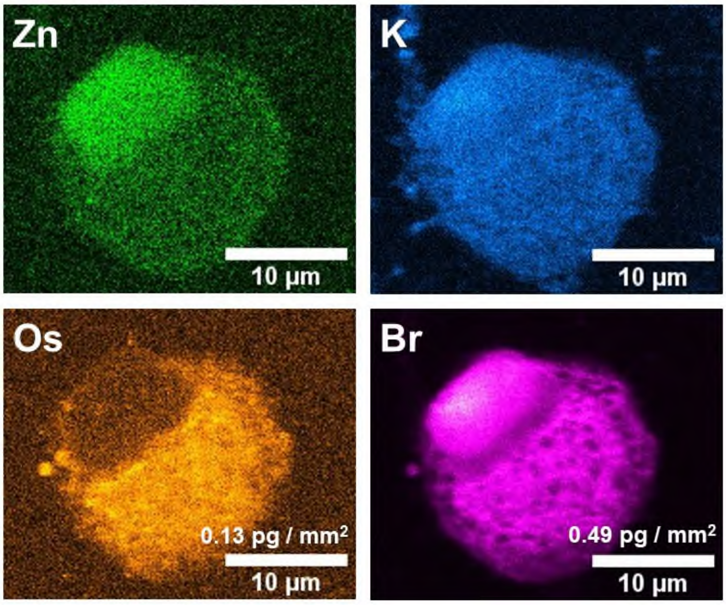

Figure S33. Synchrotron XRF elemental map of a cryo-fixed and freeze-dried A549 cell treated with 3 × IC_{50} (90 µM) of ***SS*-2** for 24 h (**C13**): (i) Zn (**green**); (ii) K (**blue**); (iii) Os (**orange**); (iv) Br (**pink**). Maps were collected using 100 nm step size and 0.1 s dwell time. Data were processed in PyMca toolkit developed by the ESRF,[10] and images generated in ImageJ.[11] Quantities of Os and Br are reported in pg mm^{-2}. Os and Br co-localize in the cell cytoplasm. However, Br is also strongly co-localized with Zn in the cell nucleus.

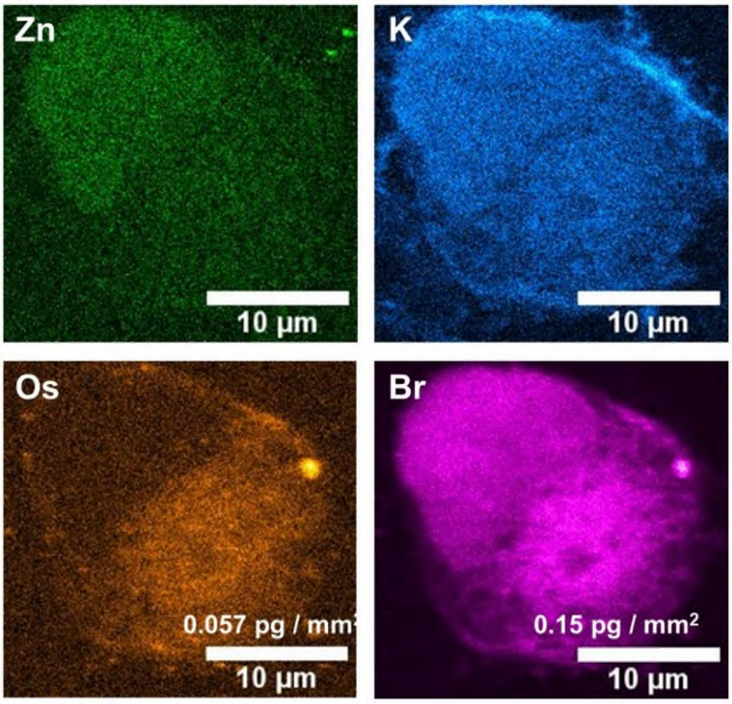

Figure S34. Synchrotron XRF elemental map of a cryo-fixed and freeze-dried A549 cell treated with 3 × IC_{50} (90 µM) of ***SS*-2** for 24 h (**C14**): (i) Zn (**green**); (ii) K (**blue**); (iii) Os (**orange**); (iv) Br (**pink**). Maps were collected using 100 nm step size and 0.1 s dwell time. Data were processed in PyMca toolkit developed by the ESRF,[10] and images generated in ImageJ.[11] Quantities of Os and Br are reported in pg mm^{-2}. Os and Br co-localize in the cell cytoplasm. However, Br is also strongly co-localized with Zn in the cell nucleus.

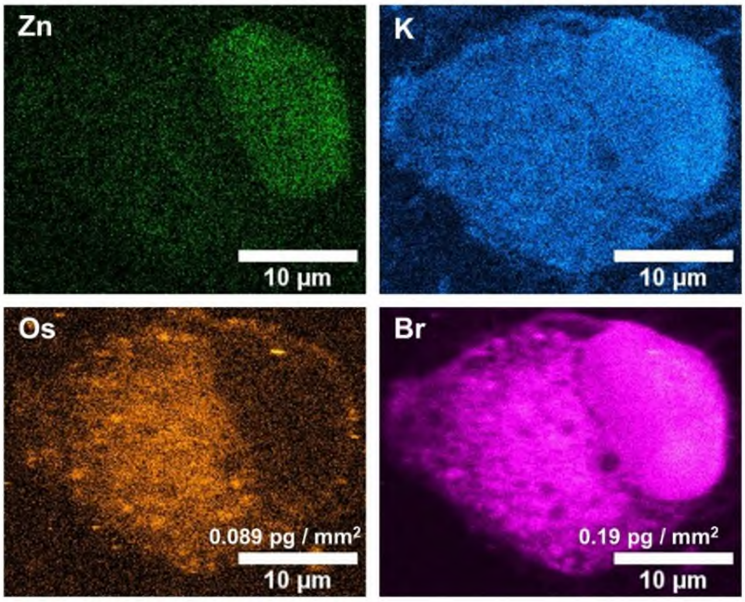

Figure S35. Synchrotron XRF elemental map of a cryo-fixed and freeze-dried A549 cell treated with 3 × IC_{50} (90 µM) of ***SS*-2** for 24 h (**C15**): (i) Zn (**green**); (ii) K (**blue**); (iii) Os (**orange**); (iv) Br (**pink**). Maps were collected using 100 nm step size and 0.1 s dwell time. Data were processed in PyMca toolkit developed by the ESRF,[10] and images generated in ImageJ.[11] Quantities of Os and Br are reported in pg mm^{-2}. Os and Br co-localize in the cell cytoplasm. However, Br is also strongly co-localized with Zn in the cell nucleus.

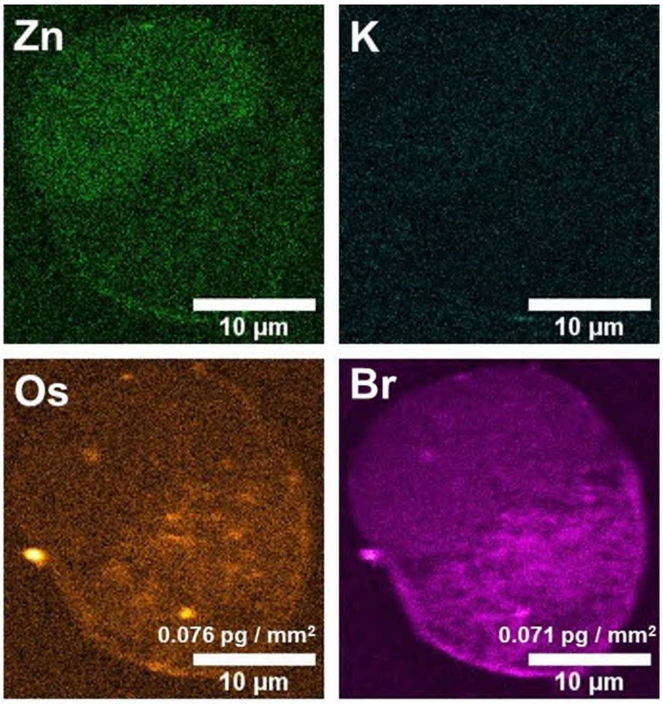

Figure S36. Synchrotron XRF elemental map of a cryo-fixed and freeze-dried A549 cell treated with 5 × IC_{50} (150 µM) of ***SS*-2** for 24 h (**C16**): (i(i) Zn (**green**); (ii) K (**blue**); (iii) Os (**orange**); (iv) Br (**pink**). Maps were collected using 100 nm step size and 0.1 s dwell time. Data were processed in PyMca toolkit developed by the ESRF,[10] and images generated in ImageJ.[11] Quantities of Os and Br are reported in pg mm^{-2}. Osmium and bromine co-localize in the cell cytoplasm. However, bromine is also strongly co-localized with zinc in the cell nucleus.

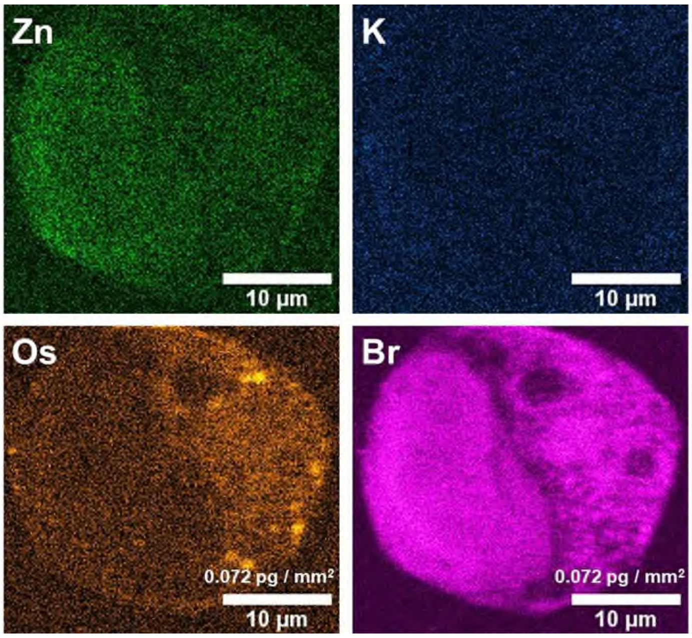

Figure S37. Synchrotron XRF elemental map of a cryo-fixed and freeze-dried A549 cell treated with 5 × IC_{50} (150 µM) of ***SS*-2** for 24 h (**C17**): (i) Zn (**green**); (ii) K (**blue**); (iii) Os (**orange**); (iv) Br (**pink**). Maps were collected using 100 nm step size and 0.1 s dwell time. Data were processed in PyMca toolkit developed by the ESRF,[10] and images generated in ImageJ.[11] Quantities of Os and Br are reported in pg mm^{-2}. Os and Br co-localize in the cell cytoplasm, however, Br is also strongly co-localized with Zn in the cell nucleus.

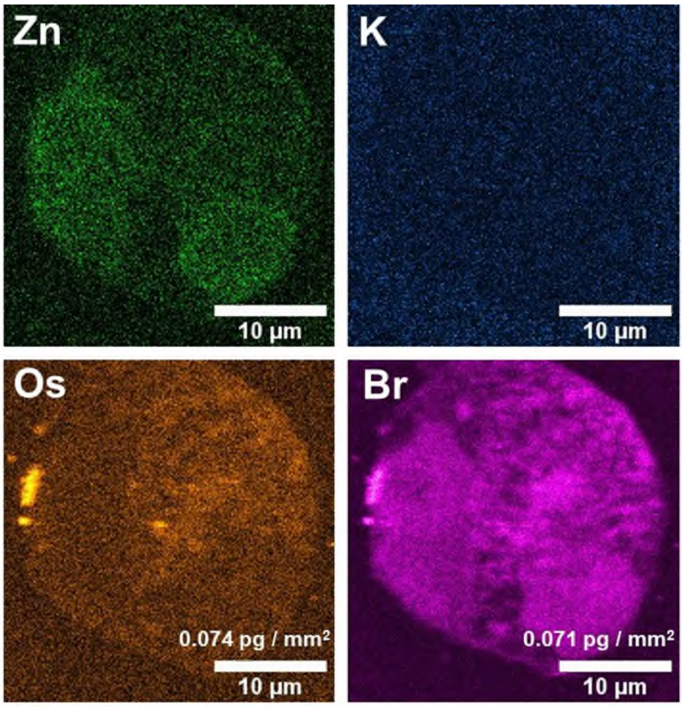

Figure S38. Synchrotron XRF elemental map of a cryo-fixed and freeze-dried A549 cell treated with 5 × IC_{50} (150 µM) of ***SS*-2** for 24 h (**C18**): (i) Zn (**green**); (ii) K (**blue**); (iii) Os (**orange**); (iv) Br (**pink**). Maps were collected using 100 nm step size and 0.1 s dwell time. Data were processed in PyMca toolkit developed by the ESRF,[10] and images generated in ImageJ.[11] Quantities of Os and Br are reported in pg mm^{-2}. Os and Br co-localize in the cell cytoplasm. However, Br is also strongly co-localized with Zn in the cell nucleus.

Cell area and roundness factor (RF)

Cell areas (µm^2) and roundness factors (a measure of circularity) of cryo-fixed and freeze-dried A549 human lung cancer cells treated with 1-3 × IC_{50} of ***SS*-2** for 24 h analysed by synchrotron XRF were determined in triplicate using ImageJ software (**Table S20**, **Figures S39-42**).[11]

Table S21. Cell areas (µm^2) and roundness factors (RF) of cryo-fixed and freeze-dried A549 cells treated with 0-5 × IC_{50} (0-150 µM) of ***SS*-2** for 24 h (no recovery), as identified by S, K, P and Zn distributions in the synchrotron-XRF elemental maps: (i) **C1-4** (0 µM); (ii) **C5-7** (1 × IC_{50}, 30 µM); (iii) **C9-15** (3 × IC_{50}, 90 µM); (iv) **C16-18** (5 × IC_{50}, 150 µM). Maps were collected using 100 nm step size and 0.1 s dwell time. Data were processed in PyMca toolkit developed by the ESRF,[10] and images generated in ImageJ.[11]

***SS*-2 / µM**	**Cell**	**Area (µm^2)**	**Mean area (µm^2)**	**Roundness factor (RF)**	**Mean RF**
**0**	**C1**	925±42	766±126	0.33±0.02	0.33±0.04
	**C2**	685±19		0.34±0.01	
	**C3**	829±23		0.36±0.02	
	**C4**	624±28		0.28±0.01	
**30**	**C5**	731±8	600±89	0.32±0.01	0.49±0.17
	**C6**	608±4		0.61±0.01	
	**C7**	501±5		0.34±0.02	
	**C8**	561±6		0.68±0.01	
**90**	**C9**	436±2	406±91	0.72±0.03	0.75±0.16
	**C10**	322±1		0.89±0.03	
	**C11**	569±3		0.43±0.02	
	**C12**	407±6		0.77±0.01	
	**C13**	278±5		0.94±0.02	
	**C14**	371±4		0.80±0.05	
	**C15**	457±3		0.719±0.004	
**150**	**C16**	484±2	567±63	0.83±0.02	0.88±0.04
	**C17**	622±4		0.886±0.003	
	**C18**	594±5		0.91±0.03	

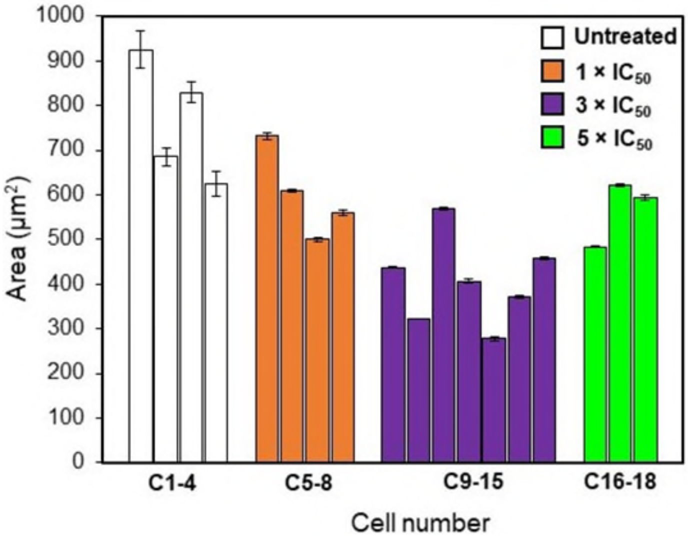

Figure S39. Individual area (µm^2) of A549 cells (**C1-18**) treated with 0-5 × IC_{50} (0-150 µM) of ***SS*-2** for 24 h (no recovery) as identified by S, K, P and Zn XRF elemental maps: (i) **C1-4** (0 µM, **white**); (ii) **C5-7** (1 × IC_{50}, 30 µM, **orange**); (iii) **C9-15** (3 × IC_{50}, 90 µM, **purple**); (iv) **C16-18** (5 × IC_{50}, 150 µM, **green**). Maps were collected using 100 nm step size and 0.1 s dwell time. Data were processed in PyMca toolkit developed by the ESRF,[10] and images generated in ImageJ.[11]

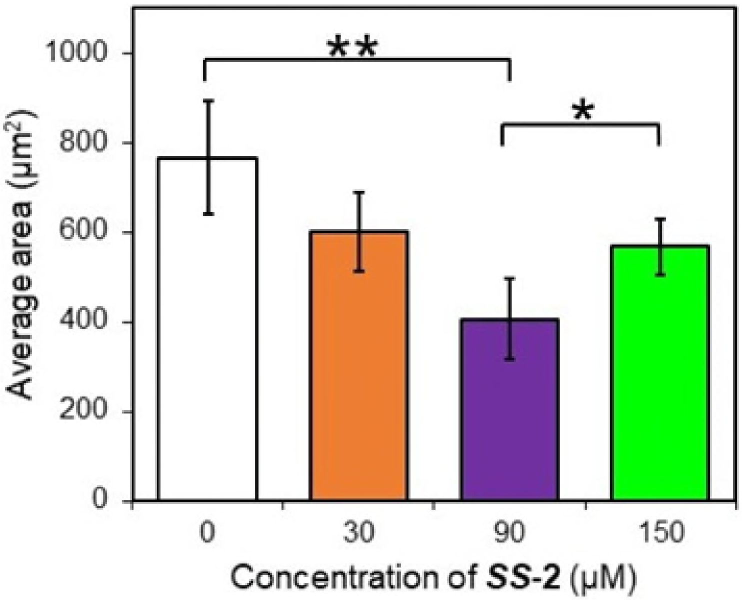

Figure S40. Average area (µm^2) of A549 cells (**C1-18**) treated with 0-5 × IC_{50} (0-150 µM) of ***SS*-2** for 24 h (no recovery) as identified by S, K, P and Zn XRF elemental maps. Maps were collected using 100 nm step size and 0.1 s dwell time. Data were processed in PyMca toolkit developed by the ESRF,[10] and images generated in ImageJ.[11] Statistical significance was calculated using a two-tailed t-test assuming unequal variances (Welch's t-test), *$p < 0.05$, **$p < 0.01$ and ***$p < 0.001$. A statistically significant decrease in cell area was observed between the negative control cells and cells treated with 3 × IC_{50} (90 µM) of ***SS*-2** (p=0.0074). A statistically significant increase in cell area was observed between cells treated with 3 × IC_{50} (90 µM) and 3 × IC_{50} (150 µM) of ***SS*-2** (p=0.0236).

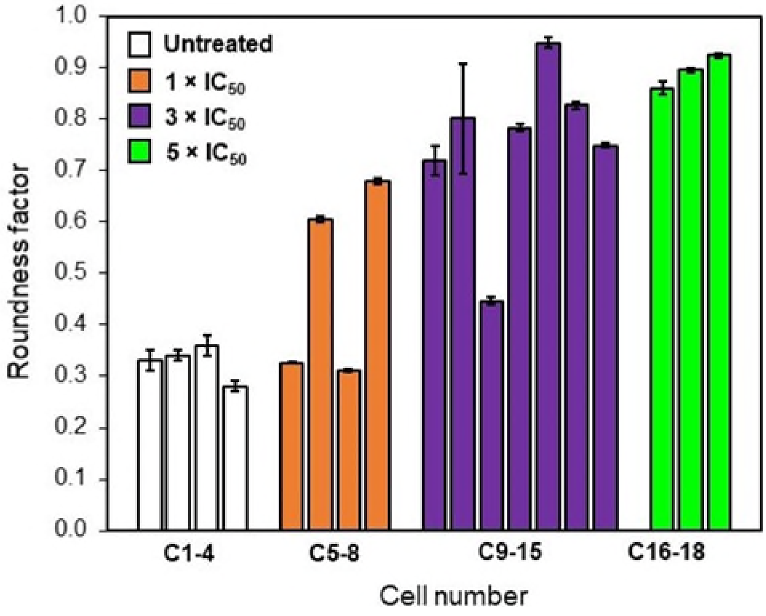

Figure S41. Individually calculated roundness factors of A549 cells (**C1-18**) treated with 0-5 × IC_{50} (0-150 µM) of ***SS*-2** for 24 h (no recovery) as identified by S, K, P and Zn XRF elemental maps: (i) **C1-4** (0 µM, **white**); (ii) **C5-7** (1 × IC_{50}, 30 µM, **orange**); (iii) **C9-15** (3 × IC_{50}, 90 µM, **purple**); (iv) **C16-18** (5 × IC_{50}, 150 µM, **green**). Maps were collected using 100 nm step size and 0.1 s dwell time. Data were processed in PyMca toolkit developed by the ESRF,[10] and images generated in ImageJ.[11]

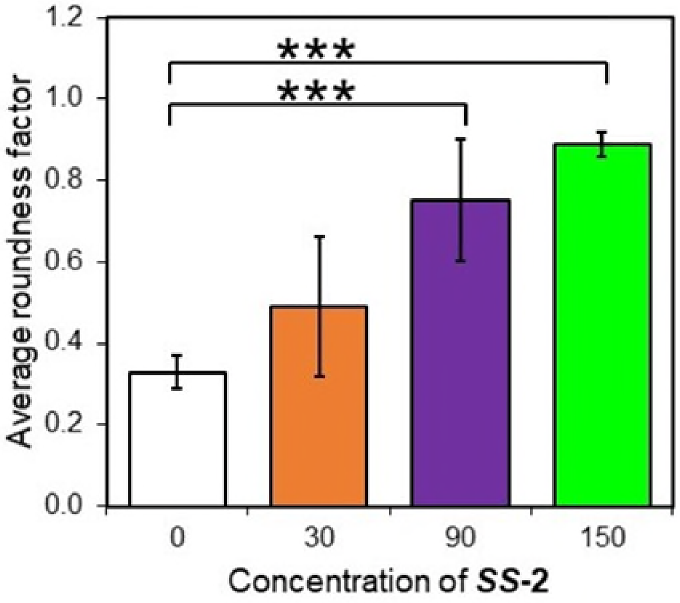

Figure S42. Average roundness factors of cryo-fixed and freeze-dried A549 (human lung) cancer cells (**C1-18**) treated with 0-5 × IC_{50} (0-150 µM) of ***SS*-2** for 24 h (no recovery) as identified by S, K, P and Zn XRF elemental maps. Maps were collected using 100 nm step size and 0.1 s dwell time. Data were processed in PyMca toolkit developed by the ESRF,[10] and images generated in ImageJ.[11] Statistical significance was calculated using a two-tailed t-test assuming unequal variances (Welch's t-test), *$p < 0.05$, **$p < 0.01$ and ***$p < 0.001$. A highly statistically significant increase in roundness factor was observed between the untreated controls and cells treated with 3 × IC_{50} (p=0.0002) and 5 × IC_{50} (p=0.0001).

XRF quantification of Os and Br

Elemental quantities of osmium and bromine in cryo-fixed and freeze-dried A549 human lung cancer cells treated with 1-3 × IC_{50} of ***SS*-2** for 24 h analysed by synchrotron XRF were determined in triplicate using ImageJ software (**Table S22** and **S23**).

Table S22. Percentage of intracellular Os and Br in regions of cryo-fixed and freeze-dried A549 (human lung) cancer cells treated with 1-5 × IC_{50} (30-150 µM) of ***SS*-2** for 24 h (no recovery), as determined by XRF. (i) **C5-7** (1 × IC_{50}, 30 µM); (ii) **C9-15** (3 × IC_{50}, 90 µM); (iii) **C16-18** (5 × IC_{50}, 150 µM). Maps were collected using 100 nm step size and 0.1 s dwell time. Data were processed in PyMca toolkit developed by the ESRF,[10] and analysed in triplicate in ImageJ.[11]

***SS*-2 / µM**	**Cell number**	**Non-nuclear Os (%)**	**Non-nuclear Br (%)**	**Nuclear Os (%)**	**Nuclear Br (%)**
**30**	**5**	83±1	83±2	17±1	17±2
	**6**	80±3	73±3	21±3	27±3
	**7**	76±3	61±1	24±3	39±1
	**8**	75±2	66±1	25±2	34±1
**90**	**9**	78±1	56±2	22±5	44±2
	**10**	85±4	63±3	15±4	37±3
	**11**	83±7	65±1	17±7	35±1
	**12**	89±2	63±2	11±2	37±2
	**13**	88±4	67±2	12±4	33±3
	**14**	86±2	57±3	14±2	43±2
	**15**	88±2	48±1	12±2	52±1
**150**	**16**	72±1	61±1	28±1	39±1
	**17**	65±1	45±1	35±1	54±1
	**18**	59±2	51±1	41±2	50±1

Table S23. Mole fraction quantities and molar Br / Os ratios in cryo-fixed and freeze-dried A549 (human lung) cancer cells treated with 1-5 × IC_{50} (30-150 µM) of ***SS*-2** for 24 h (no recovery), as analysed by XRF: (i) **C5-7** (1 × IC_{50}, 30 µM); (ii) **C9-15** (3 × IC_{50}, 90 µM); (iii) **C16-18** (5 × IC_{50}, 150 µM). Maps were collected using 100 nm step size and 0.1 s dwell time. Data were processed in PyMca toolkit developed by the ESRF,[10] and analysed in triplicate ImageJ.[11]

***SS*-2 / µM**	**Cell number**	**Mole Fraction of [Os] × 10^{-8}**	**Mole Fraction of [Br] × 10^{-8}**	**[Br]/[Os]**	**Mean [Br] / [Os]**
**30**	**5**	5.41±0.02	46.5±0.3	8.6±0.03	5±2.5
	**6**	5.22±0.01	14.86±0.04	2.9±0.01	
	**7**	4.70±0.01	19.6±0.1	4.2±0.01	
	**8**	5.54±0.02	23.48±0.15	4.2±0.02	
**90**	**9**	4.390±0.004	34.1±0.2	7.8±0.01	7.1±2.4
	**10**	5.50±0.01	37.0±0.9	6.7±0.01	
	**11**	6.82±0.01	25.9±0.1	3.8±0.06	
	**12**	8.72±0.05	98.4±0.8	11.3±0.06	
	**13**	9.91±0.07	86.4±0.9	8.7±0.01	
	**14**	4.20±0.01	26.0±0.2	6.4±0.01	
	**15**	6.53±0.01	33.7±0.1	5.2±0.01	
**150**	**16**	5.60±0.02	12.55±0.03	2.2±0.01	2.3±0.1
	**17**	5.29±0.01	12.89±0.02	2.4±0.01	
	**18**	5.47±0.02	12.49±0.04	2.3±0.01	

Elemental co-localisation of Os and Br

The colocalization between osmium and bromine in cryo-fixed and freeze-dried A549 human lung cancer cells treated with 1-5 × IC_{50} of ***SS*-2** for 24 h analysed by synchrotron XRF were determined in triplicate using ImageJ software. Colocalisation between osmium and bromine is reported as Pearson R-value's and Speark Rank Correlations in the whole cell, cytoplasm and the nucleus (**Table S24**).

Table S24. Pearson R-value and Spearman Rank Correlation between Os and Br in cryo-fixed and freeze-dried A549 (human lung) cancer cells treated with 1-5 × IC_{50} (0-150 µM) of ***SS*-2** for 24 h (no recovery) as analysed by XRF: (i) **C5-7** (1 × IC_{50}, 30 µM); (ii) **C9-15** (3 × IC_{50}, 90 µM); (iii) **C16-18** (5 × IC_{50}, 150 µM). Maps were collected using 100 nm step size and 0.1 s dwell time. Data were processed in PyMca toolkit developed by the ESRF,[10] and analysed in triplicate in ImageJ.[11] A positive correlation was observed between Os and Br in the cytoplasm and in whole cells.

***SS*-2 / µM**	**Cell**	**Pearson R-value**			**Spearman Rank Correlation**		
		**All**	**Cytoplasm**	**Nucleus**	**All**	**Cytoplasm**	**Nucleus**
**30**	**C5**	0.45	0.13	-0.02	0.346	0.120	-0.022
	**C6**	0.29	0.19	0.10	0.284	0.178	0.093
	**C7**	0.15	0.21	0.00	0.125	0.206	-0.003
	**C8**	0.42	0.42	0.04	0.364	0.364	-0.030
**90**	**C9**	0.48	0.42	-0.08	0.417	0.345	-0.073
	**C10**	0.56	0.48	0.03	0.510	0.469	-0.031
	**C11**	0.43	0.33	0	0.397	0.316	0.002
	**C12**	0.43	0.38	-0.08	0.406	0.349	-0.079
	**C13**	0.09	0.32	-0.05	0.139	0.301	-0.062
	**C14**	0.24	0.39	-0.05	0.160	0.356	-0.043
	**C15**	0.07	0.41	-0.11	0.074	0.423	-0.13
**150**	**C16**	0.22	0.18	0.08	0.214	0.172	0.043
	**C17**	0.11	0.16	0.02	0.100	0.147	0.022
	**C18**	0.22	0.17	0.04	0.139	0.159	0.031

XRF lysosomal structures

Regions of concentrated osmium in cryo-fixed and freeze-dried A549 human lung cancer cells treated with 1-5 × IC_{50} of ***SS*-2** for 24 h analysed by synchrotron XRF were identified. The areas (µm^2) of these compartments were determined in triplicate from the Os XRF maps using the multipoint selection tools in ImageJ.[11] and both the Os and Br in these regions were quantified (**Figures S43-53**, **Table S25**).

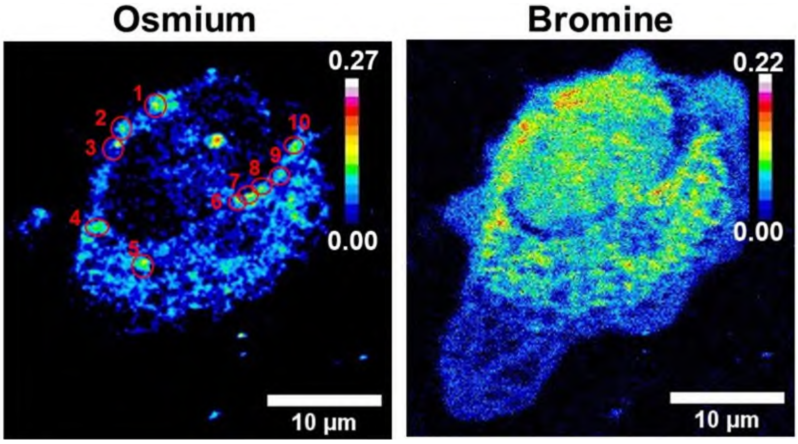

Figure S43. Synchrotron-XRF elemental maps of a cryo-fixed and freeze-dried A549 (human lung) cancer cell (**C5**) treated with 1× IC_{50} of ***SS*-2** (30 µM) for 24 h (no recovery). Maps were collected using 100 nm step size and 0.1 s dwell time. Data were processed in PyMca toolkit developed by the ESRF,[10] and images generated in ImageJ using the 16-colour setting.[11] The calibration bar is in pg mm^{-2}. Red circles represent small vesicle-sized regions of co-localisation of Os and Br. A total of 10 lysosomes were identified with an average area of 0.67±0.22 µm^2 and a calculated average molar bromine-to-osmium (Br / Os) ratio of 2.74±0.24.

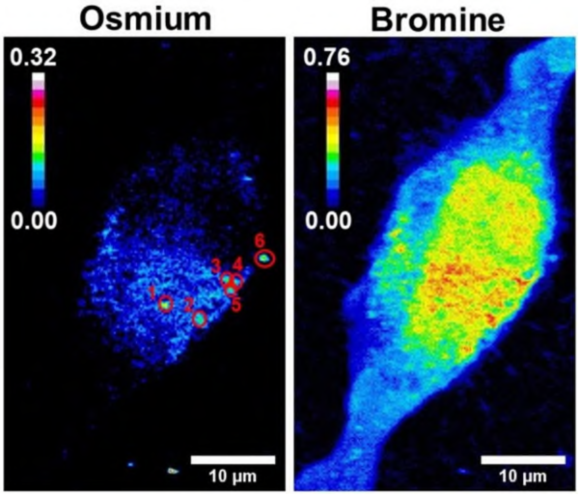

Figure S44. Synchrotron-XRF elemental maps of a cryo-fixed and freeze-dried A549 (human lung) cancer cell (**C6**) treated with 1× IC_{50} of ***SS*-2** (30 µM) for 24 h (no recovery). Maps were collected using 100 nm step size and 0.1 s dwell time. Data were processed in PyMca toolkit developed by the ESRF,[10] and images generated in ImageJ using the 16-colour setting.[11] The calibration bar is in pg mm^{-2}. Red circles represent small vesicle-sized regions of co-localisation of Os and Br. A total of 6 lysosomes were identified with an average area of 0.62±0.23 µm^2 and a calculated average molar bromine-to-osmium (Br / Os) ratio of 6.9±2.1.

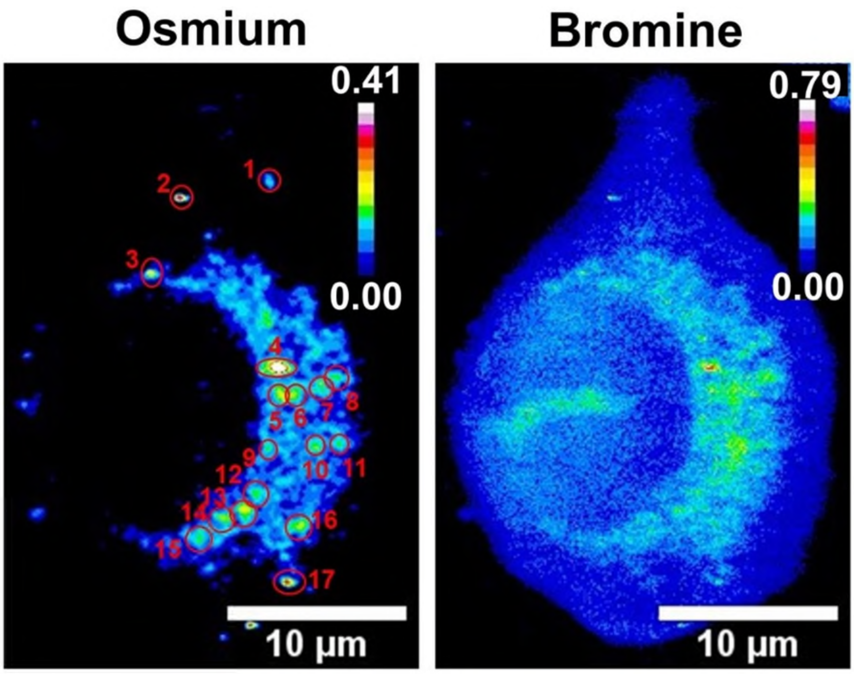

Figure S45. Synchrotron-XRF elemental maps of a cryo-fixed and freeze-dried A549 (human lung) cancer cell (**C9**) treated with 3× IC_{50} of ***SS*-2** (90 µM) for 24 h (no recovery). Maps were collected using 100 nm step size and 0.1 s dwell time. Data were processed in PyMca toolkit developed by the ESRF,[10] and images generated in ImageJ using the 16-colour setting.[11] The calibration bar is in pg mm^{-2}. Red circles represent small vesicle-sized regions of co-localisation of Os and Br. A total of 17 lysosomes were identified with an average area of 0.63±0.29 μm^2 and a calculated average molar bromine-to-osmium (Br / Os) ratio of 3.4±0.4.

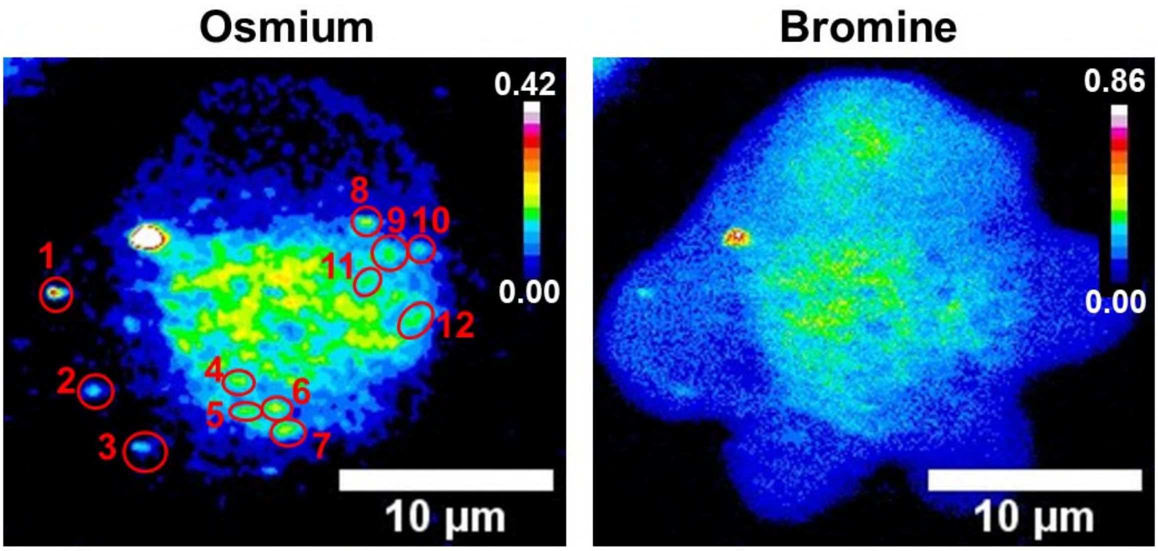

Figure S46. Synchrotron-XRF elemental maps of a cryo-fixed and freeze-dried A549 (human lung) cancer cell (**C10**) treated with 3× IC_{50} of ***SS*-2** (90 µM) for 24 h (no recovery). Maps were collected using 100 nm step size and 0.1 s dwell time. Data were processed in PyMca toolkit developed by the ESRF,[10] and images generated in ImageJ using the 16-colour setting.[11] The calibration bar is in pg mm^{-2}. Red circles represent small vesicle-sized regions of co-localisation of Os and Br. A total of 12 lysosomes were identified with an average area of 0.57±0.23 μm^2 and a calculated average molar bromine-to-osmium (Br / Os) ratio of 3.1±0.6.

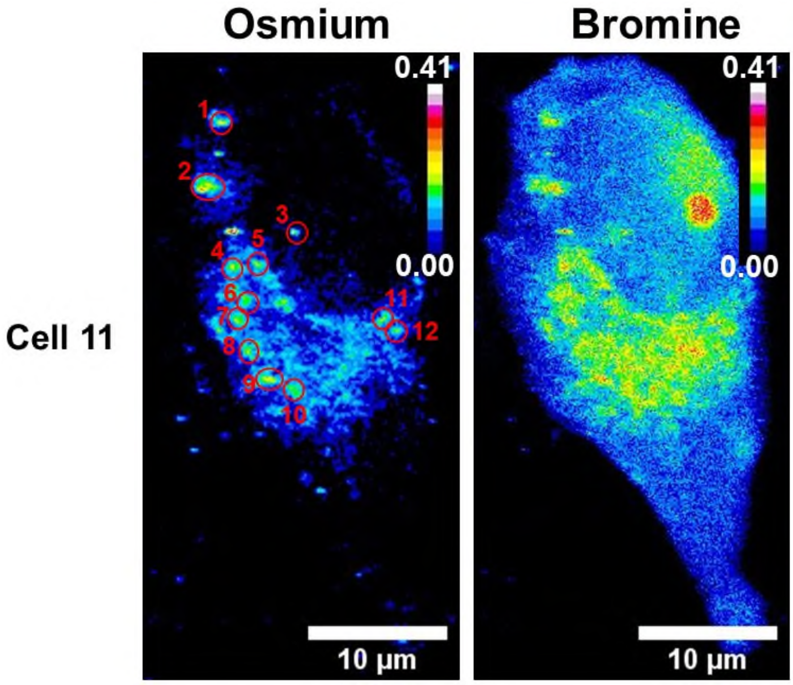

Figure S47. Synchrotron-XRF elemental maps of a cryo-fixed and freeze-dried A549 (human lung) cancer cell (**C11**) treated with 3× IC_{50} of ***SS*-2** (90 µM) for 24 h (no recovery). Maps were collected using 100 nm step size and 0.1 s dwell time. Data were processed in PyMca toolkit developed by the ESRF,[10] and images generated in ImageJ using the 16-colour setting.[11] The calibration bar is in pg mm^{-2}. Red circles represent small vesicle-sized regions of co-localisation of Os and Br. A total of 12 lysosomes were identified with an average area of 0.70±0.17 μm^2 and a calculated average molar bromine-to-osmium (Br / Os) ratio of 2.6±0.3.

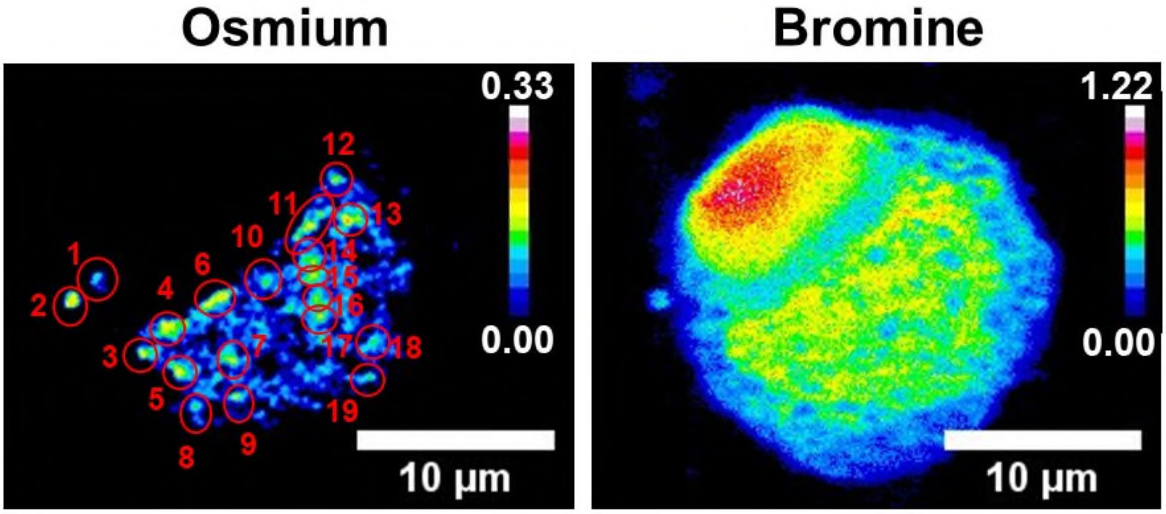

Figure S48. Synchrotron-XRF elemental maps of a cryo-fixed and freeze-dried A549 (human lung) cancer cell (**C12**) treated with 3× IC_{50} of ***SS*-2** (90 µM) for 24 h (no recovery). Maps were collected using 100 nm step size and 0.1 s dwell time. Data were processed in PyMca toolkit developed by the ESRF,[10] and images generated in ImageJ using the 16-colour setting.[11] The calibration bar is in pg mm^{-2}. Red circles represent small vesicle-sized regions of co-localisation of Os and Br. A total of 19 lysosomes were identified with an average area of 0.67±0.19 μm^2 and a calculated average molar bromine-to-osmium (Br / Os) ratio of 5.7±0.8.

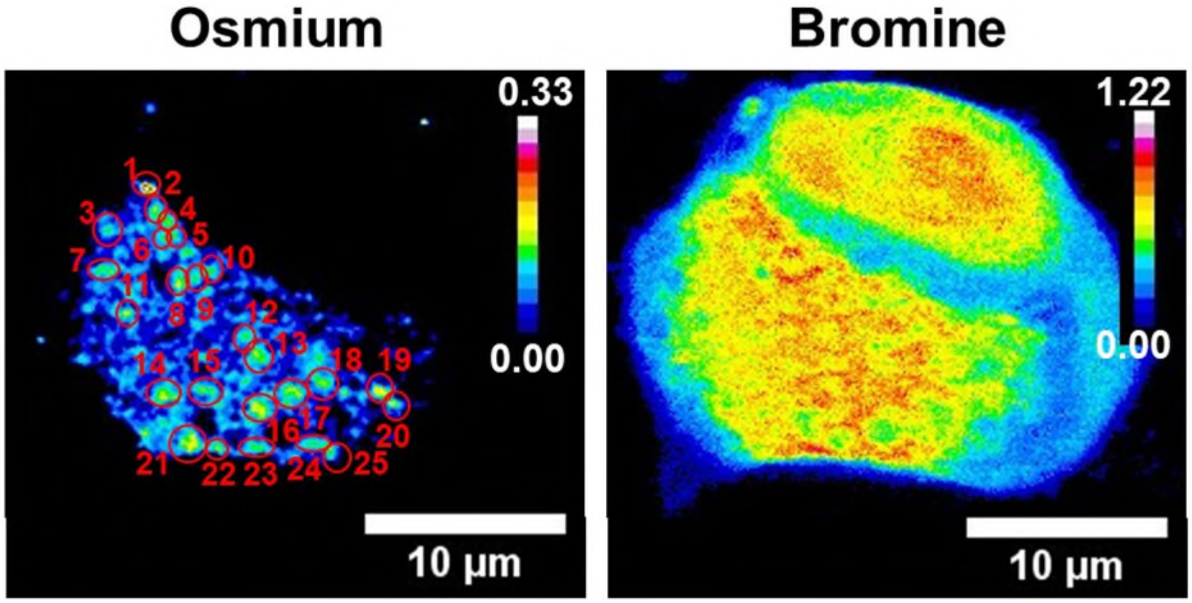

Figure S49. Synchrotron-XRF elemental maps of a cryo-fixed and freeze-dried A549 (human lung) cancer cell (**C13**) treated with 3× IC_{50} of ***SS*-2** (90 µM) for 24 h (no recovery). Maps were collected using 100 nm step size and 0.1 s dwell time. Data were processed in PyMca toolkit developed by the ESRF,[10] and images generated in ImageJ using the 16-colour setting.[11] The calibration bar is in pg mm^{-2}. Red circles represent small vesicle-sized regions of co-localisation of Os and Br. A total of 25 lysosomes were identified with an average area of 0.45±0.15 μm^2 and a calculated average bromine-to-osmium (Br / Os) ratio of 8.5±0.8.

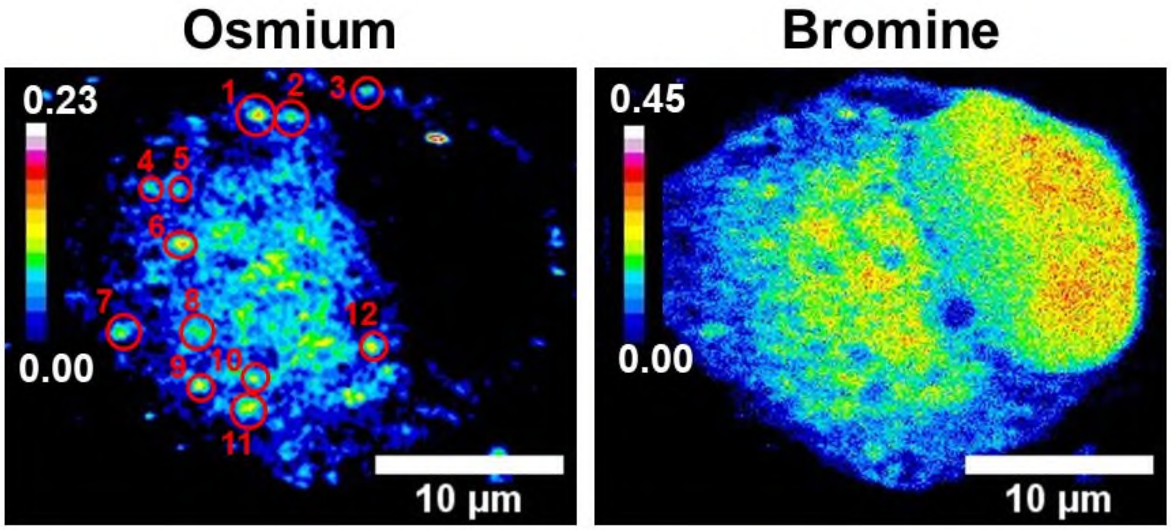

Figure S50. Synchrotron-XRF elemental maps of a cryo-fixed and freeze-dried A549 (human lung) cancer cell (**C15**) treated with 3 × IC_{50} (90 µM) of ***SS*-2** for 24 h (no recovery). Maps were collected using 100 nm step size and 0.1 s dwell time. Data were processed in PyMca toolkit developed by the ESRF,[10] and images generated in ImageJ using the 16-colour setting.[11] The calibration bar is in pg mm^{-2}. Red circles represent small vesicle-sized regions of co-localisation of Os and Br. A total of 12 lysosomes were identified with an average area of 0.58±0.18 μm^2 and a calculated average bromine-to-osmium (Br / Os) ratio of 3.6±0.4.

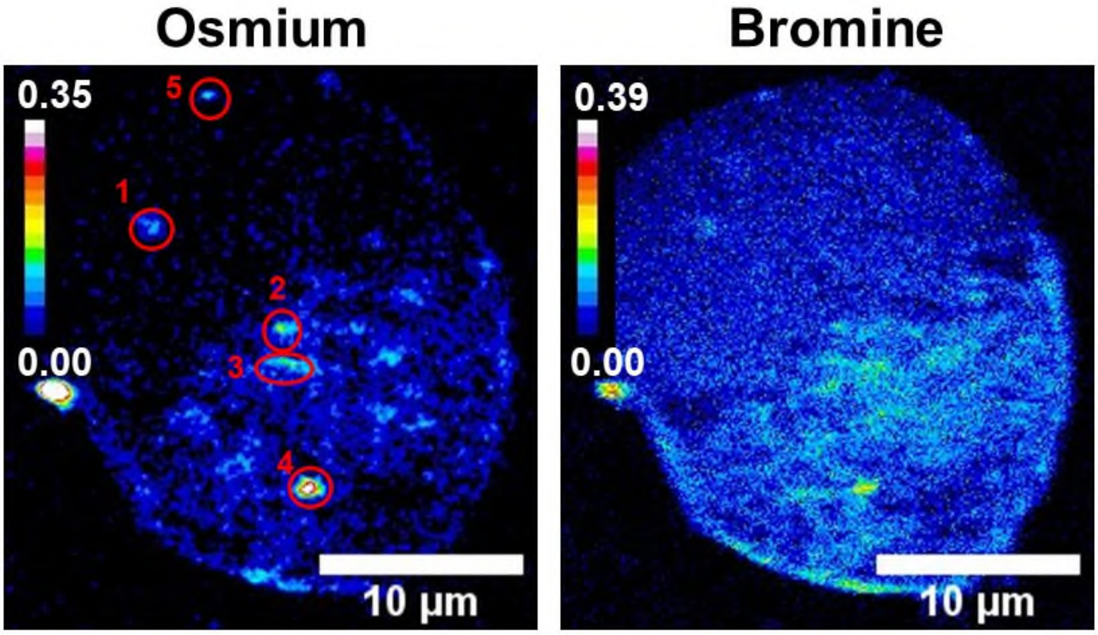

Figure S51. Synchrotron-XRF elemental maps of a cryo-fixed and freeze-dried A549 (human lung) cancer cell (**C16**) treated with 5×IC_{50} (150 µM) of ***SS*-2** for 24 h (no recovery). Maps were collected using 100 nm step size and 0.1 s dwell time. Data were processed in PyMca toolkit developed by the ESRF,[10] and images generated in ImageJ using the 16-colour setting.[11] The calibration bar is in pg mm^{-2}. Red circles represent small vesicle-sized regions of co-localisation of Os and Br. A total of 5 lysosomes were identified with an average area of 0.84±0.42 μm^2 and a calculated average bromine-to-osmium (Br/Os) ratio of 1.57±0.14.

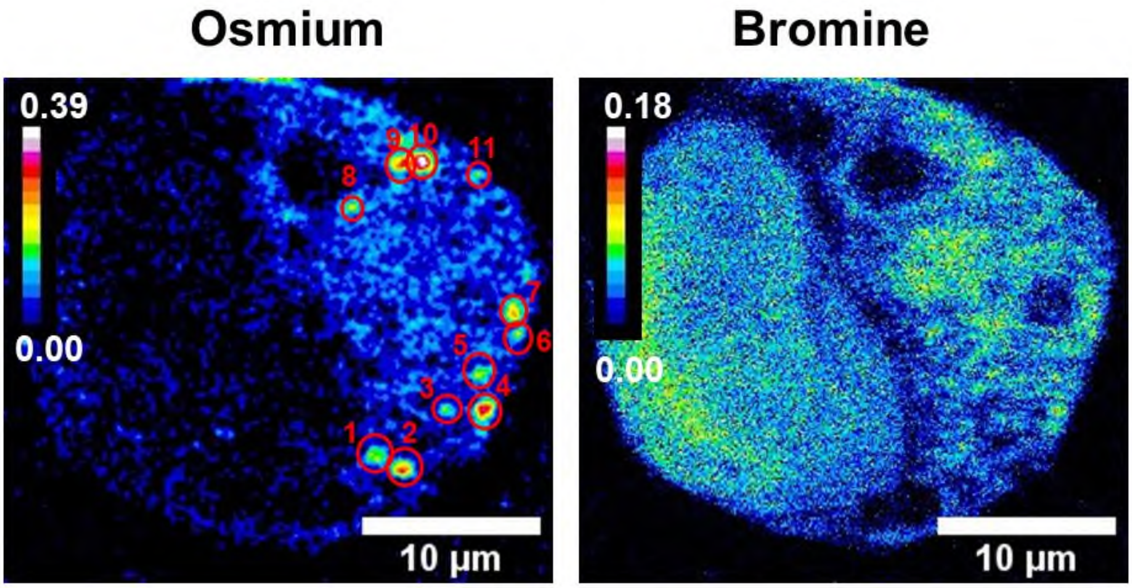

Figure S52. Synchrotron-XRF elemental maps of a cryo-fixed and freeze-dried A549 (human lung) cancer cell (**C17**) treated with 5×IC_{50} (150 µM) of ***SS*-2** for 24 h (no recovery). Maps were collected using 100 nm step size and 0.1 s dwell time. Data were processed in PyMca toolkit developed by the ESRF,[10] and images generated in ImageJ using the 16-colour setting.[11] The calibration bar is in pg mm^{-2}. Red circles represent small vesicle-sized regions of co-localisation of Os and Br. A total of 11 lysosomes were identified with an average area of 0.80±0.28 μm^2 and a calculated average bromine-to-osmium (Br/Os) ratio of 1.53±0.12.

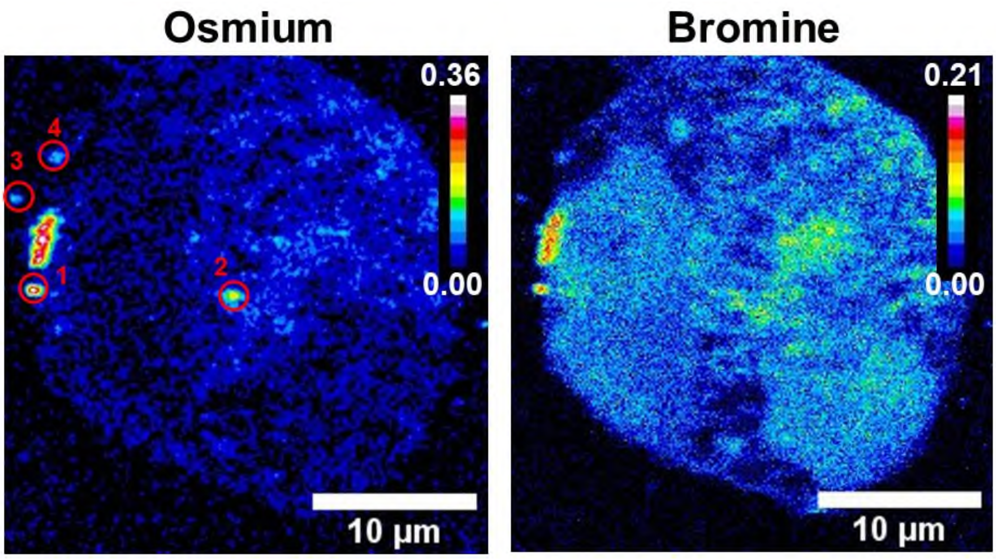

Figure S53. Synchrotron-XRF elemental maps of a cryo-fixed and freeze-dried A549 (human lung) cancer cell (**C18**) treated with 5×IC_{50} (150 µM) of ***SS*-2** for 24 h (no recovery). Maps were collected using 100 nm step size and 0.1 s dwell time. Data were processed in PyMca toolkit developed by the ESRF,[10] and images generated in ImageJ using the 16-colour setting.[11] The calibration bar is in pg mm^{-2}. Red circles represent small vesicle-sized regions of co-localisation of Os and Br. A total of 4 lysosomes were identified with an average area of 0.63±0.05 μm^2 and a calculated average bromine-to-osmium (Br/Os) ratio of 1.24±0.13.

Table S25. A comparison of the average lysosomal area (μm^2) and the average lysosomal molar bromine-to-osmium (Br / Os) ratio of cryo-fixed and freeze-dried A549 (human lung) cancer cells treated with 3 and 5×IC_{50} (90 µM) of ***SS*-2** for 24 h (no recovery), as determined by XRF: (i) **C9-15** (3 × IC_{50}, 90 µM); (ii) **C16-18** (5 × IC_{50}, 150 µM). Maps were collected using 100 nm step size and 0.1 s dwell time. Data were processed in PyMca toolkit developed by the ESRF,[10] and analysed in triplicate in ImageJ.[11]

***SS*-2 / µM**	**Cell number**	**No. of lysosomes**	**Average lysosomal area (μm^2)**	**Mean molar Br / Os**
**30**	**5**	10	0.67±0.22	2.74±0.25
	**6**	6	0.62±0.23	6.9±2.1
	**7**	n.d	n.d	n.d
	**8**	n.d	n.d	n.d
**90**	**9**	17	0.63±0.29	3.4±0.4
	**10**	12	0.57±0.23	3.1±0.6
	**11**	12	0.70±0.17	2.6±0.3
	**12**	19	0.67±0.19	5.7±0.8
	**13**	25	0.45±0.15	8.5±0.8
	**14**	n.d	n.d	n.d
	**15**	12	0.58±0.18	3.6±0.4
**150**	**16**	5	0.84±0.42	1.57±0.14
	**17**	11	0.80±0.18	1.53±0.12
	**18**	4	0.63±0.05	1.24±0.13

ES15 Reactions with L-cysteine

Solution stability tests of ***RR*-1** and ***SS*-2** in the presence of L-cysteine were performed to model a lysosomal environment at acidic (5.5) and neutral pH (7.10). Solutions of 100 µM ***SS*-1** and ***SS*-2** were prepared in 5% *v/v* DMSO and 95% ammonium acetate (25 mM, pH 7.10) containing 0 - 10 mM of L-cysteine and incubated for 24 h (310 K). Similarly, solutions of ***SS*-1** and ***SS*-2** were prepared in 5% *v/v* DMSO and 95% ammonium acetate buffer (25 mM, pH 5.5 – adjusted with acetic acid) containing 0 - 10 mM L-cysteine for 24 h (310 K).

Table S26. ESI-MS *m*/*z* data showing the chemical instability of ***RR*-1** and ***SS*-2** in the presence of L-cysteine (1-10 mM) at pH 5.5 and 7 for 24 h at 310 K to model a lysosomal environment (pH 4.5-5.5 with millimolar concentrations of cysteine proteases).[12] $[M+H]^+$ ions were detected.

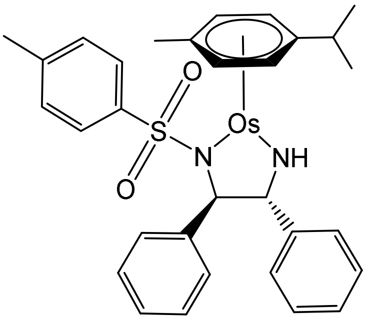

***RR*-1**
MW = 688.91

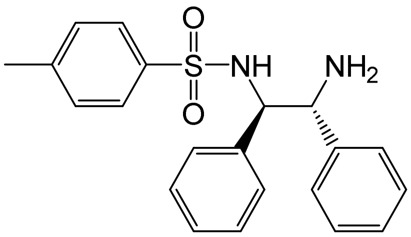

TsDPEN
MW = 366.14

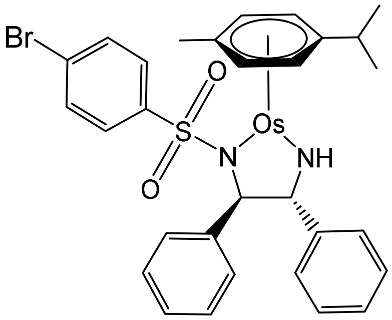

***RR*-2**
MW = 753.78

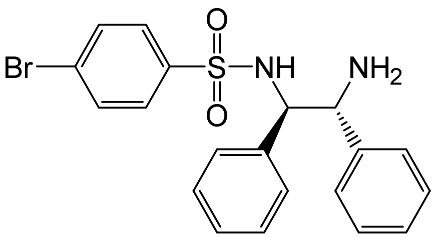

***RR*-L1**
MW = 431.35

			**$[M + H]^+$**	
**Complex**	**pH**	**0 mM L-Cys**	**1 mM L-Cys**	**10 mM L-Cys**
***RR*-1**	5.5	691.2029<br>$[C_{31}H_{34}N_2O_2OsS + H]^+$	367.1475<br>$[C_{21}H_{22}N_2O_2S + H]^+$	367.1476<br>$[C_{21}H_{22}N_2O_2S + H]^+$
	7	691.2026<br>$[C_{31}H_{34}N_2O_2OsS + H]^+$	367.1474<br>$[C_{21}H_{22}N_2O_2S + H]^+$	367.1471<br>$[C_{21}H_{22}N_2O_2S + H]^+$
***RR*-2**	5.5	755.0953<br>$[C_{30}H_{31}BrN_2O_2OsS + H]^+$	431.0419<br>$[C_{20}H_{19}BrN_2O_2S + H]^+$	431.0424<br>$[C_{20}H_{19}BrN_2O_2S + H]^+$
	7	755.0957<br>$[C_{30}H_{31}BrN_2O_2OsS + H]^+$	431.0421<br>$[C_{20}H_{19}BrN_2O_2S + H]^+$	431.0425<br>$[C_{20}H_{19}BrN_2O_2S + H]^+$

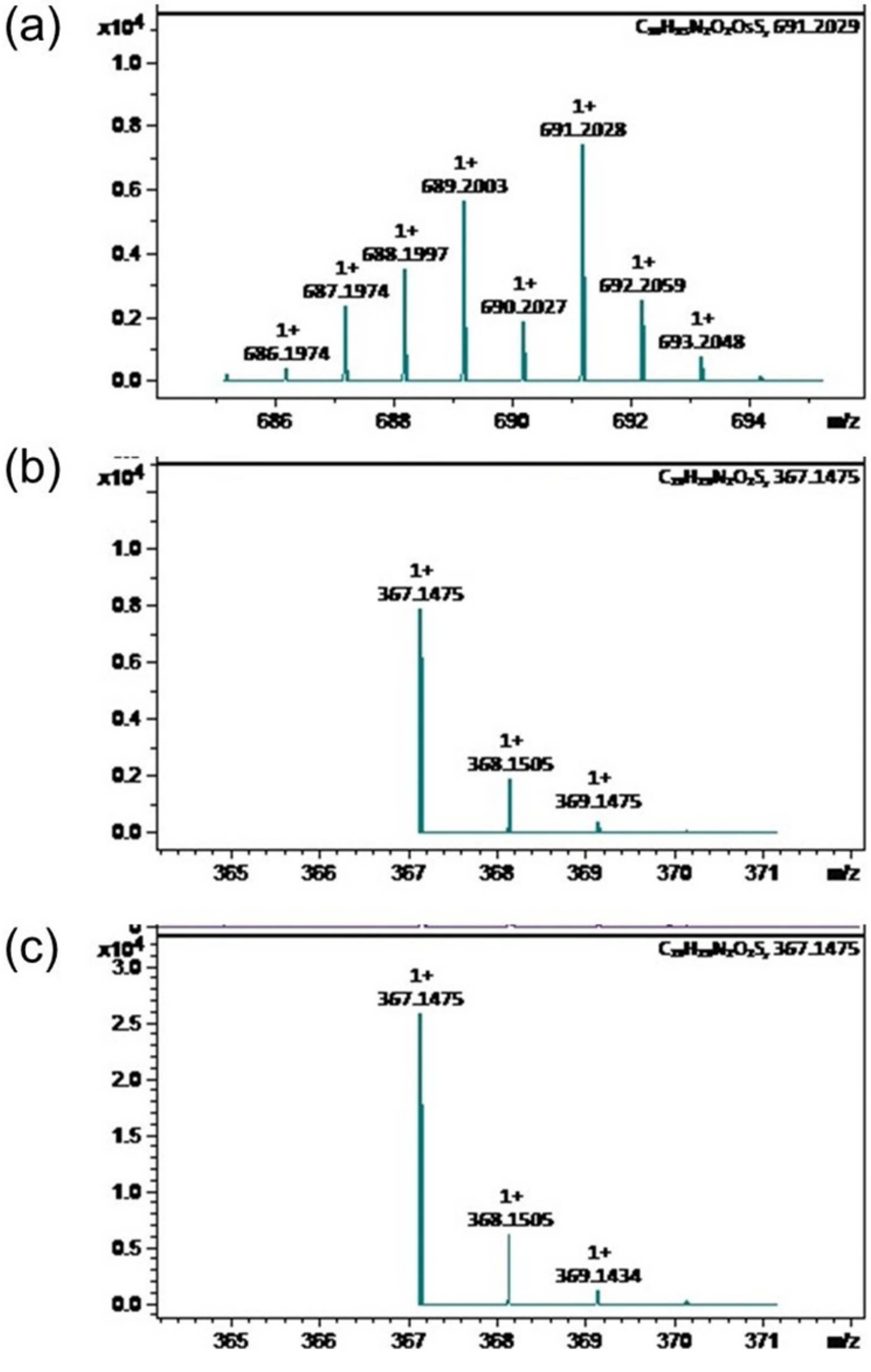

Figure S54. HR-MS of the solution resulting from incubating ***RR*-1** (MW = $C_{31}H_{34}N_2O_2OsS$) at pH 7 (prepared in ammonium acetate) for 24 h in the presence of 0, 1 or 10 mM of L-cysteine. (a) 0 mM L-cysteine - ***RR*-1** remains intact in solution ($[C_{31}H_{34}N_2O_2OsS + H]^+$ = 691.2029). (b) 1 mM L-cysteine - ***RR*-1** dissociates in the presence of L-cysteine, releasing the MePh-TsDPEN ligand in solution ($[C_{21}H_{22}N_2O_2S + H]^+$ = 367.1475). No peak at 691.2029 was observed. (c) 10 mM L-cysteine - ***RR*-1** dissociates in the presence of L-cysteine, releasing the MePh-TsDPEN ligand in solution ($[C_{21}H_{22}N_2O_2S + H]^+$ = 367.1475). No peak at 691.2029 was observed.

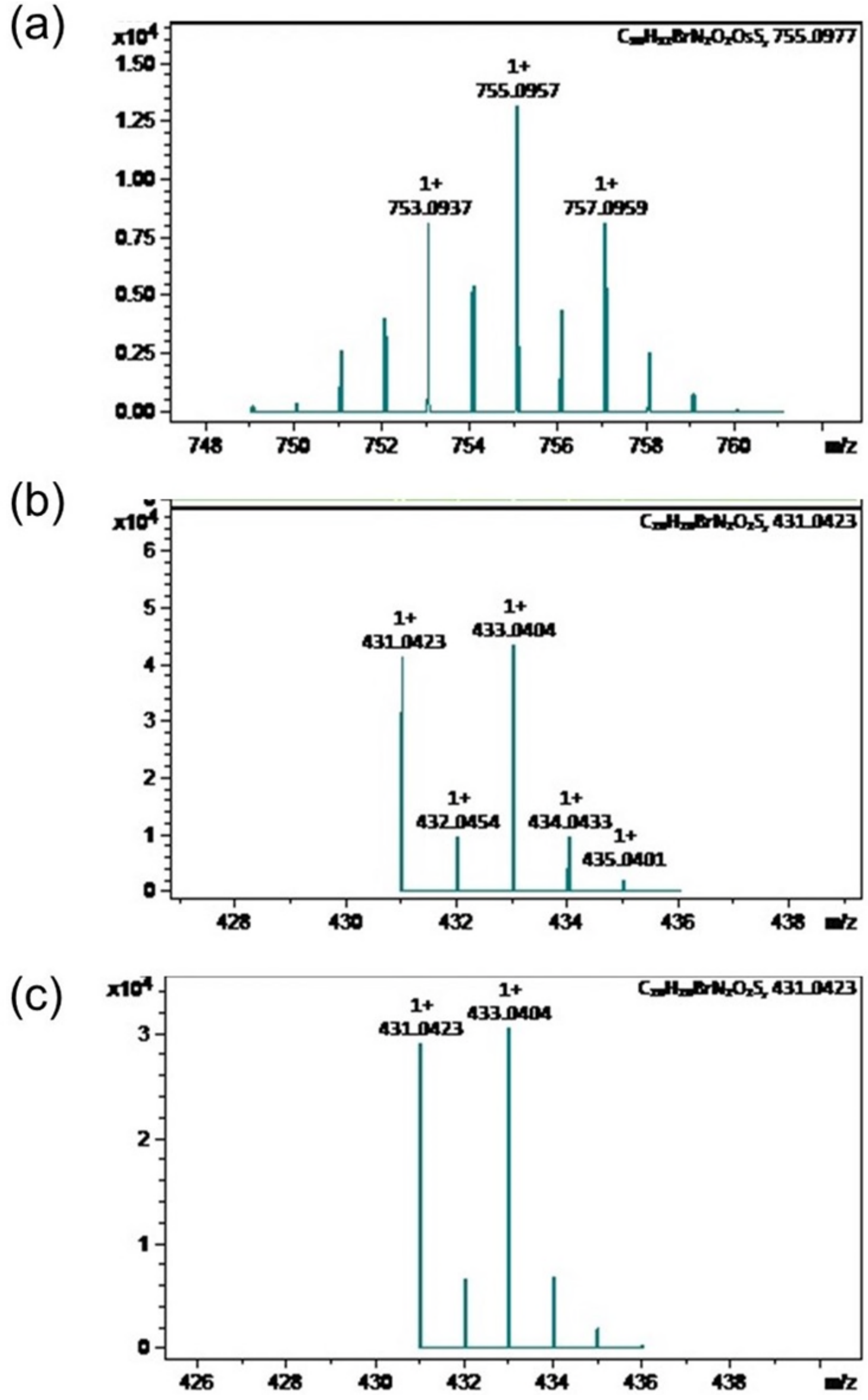

Figure S55. HR-MS of the solution resulting from incubating ***RR*-2** (MW = 753.78) at pH 7 (prepared in ammonium acetate) for 24 h in the presence of 0, 1 or 10 mM of L-cysteine. (a) 0 mM L-cysteine - ***RR*-1** remains intact in solution ($[C_{30}H_{31}BrN_2O_2OsS + H]^+$ = 755.0957). (b) 1 mM L-cysteine - ***RR*-2** dissociates in the presence of L-cysteine, releasing the BrPh-TsDPEN ligand in solution ($[C_{20}H_{19}BrN_2O_2S + H]^+$ = 431.0423). No peak at 755.0957 was observed. (c) 10 mM L-cysteine - ***RR*-2** dissociates in the presence of L-cysteine, releasing the BrPh-TsDPEN ligand in solution $[C_{20}H_{19}BrN_2O_2S + H]^+$ = 431.0423). No peak at 755.0957 was observed.

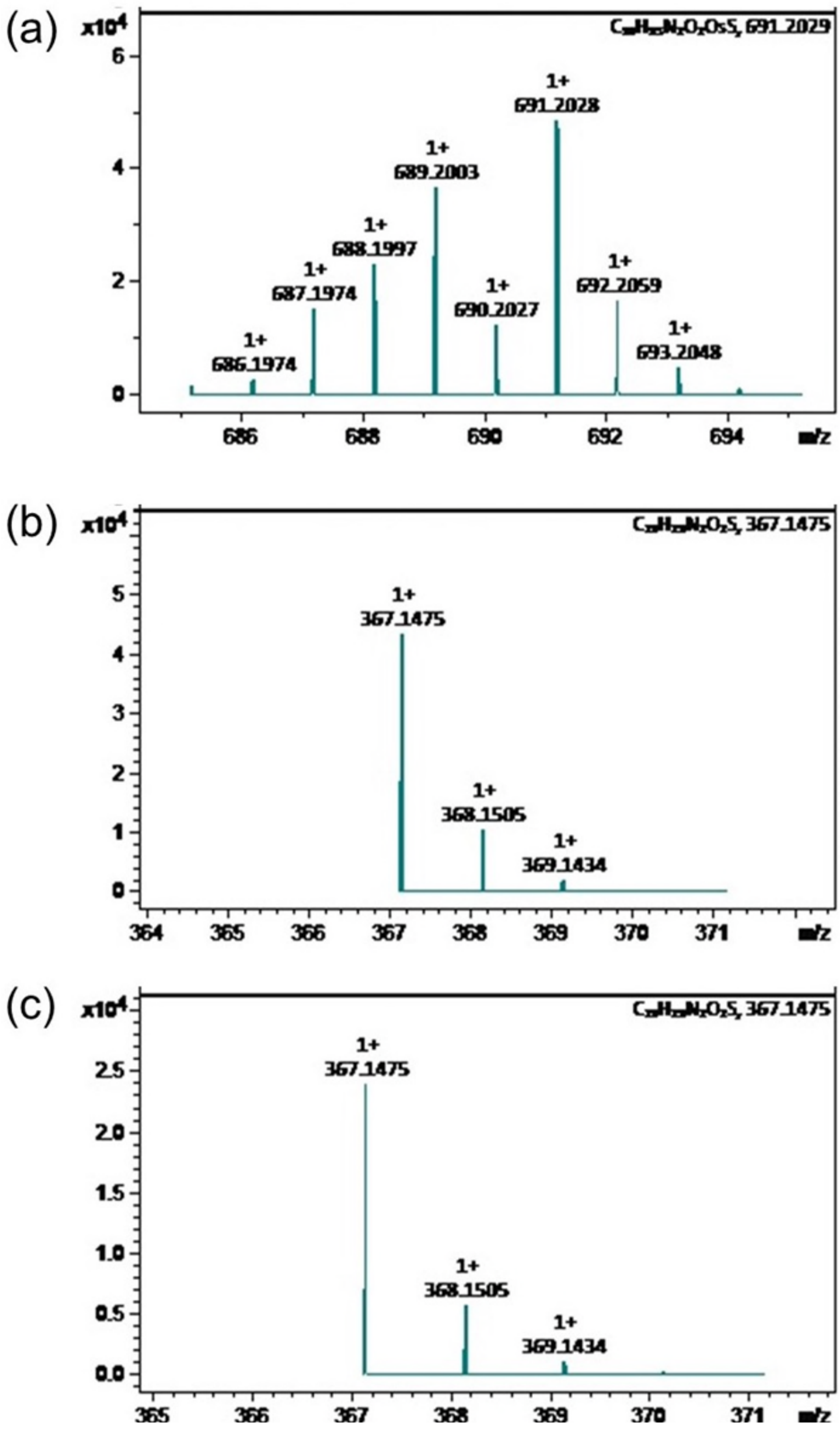

Figure S56. HR-MS of the solution resulting from incubating ***RR*-1** (MW = $C_{31}H_{34}N_2O_2OsS$) at pH 5.5 (prepared in ammonium acetate) for 24 h in the presence of 0, 1 or 10 mM of L-cysteine. (a) 0 mM L-cysteine - ***RR*-1** remains intact in solution ($[C_{31}H_{34}N_2O_2OsS + H]^+$ = 691.2029). (b) 1 mM L-cysteine - ***RR*-1** dissociates in the presence of L-cysteine, releasing the MePh-TsDPEN ligand in solution ($[C_{21}H_{22}N_2O_2S + H]^+$ = 367.1475). No peak at 691.2029 was observed. (c) 10 mM L-cysteine - ***RR*-1** dissociates in the presence of L-cysteine, releasing the MePh-TsDPEN ligand in solution ($[C_{21}H_{22}N_2O_2S + H]^+$ = 367.1475). No peak at 691.2029 was observed.

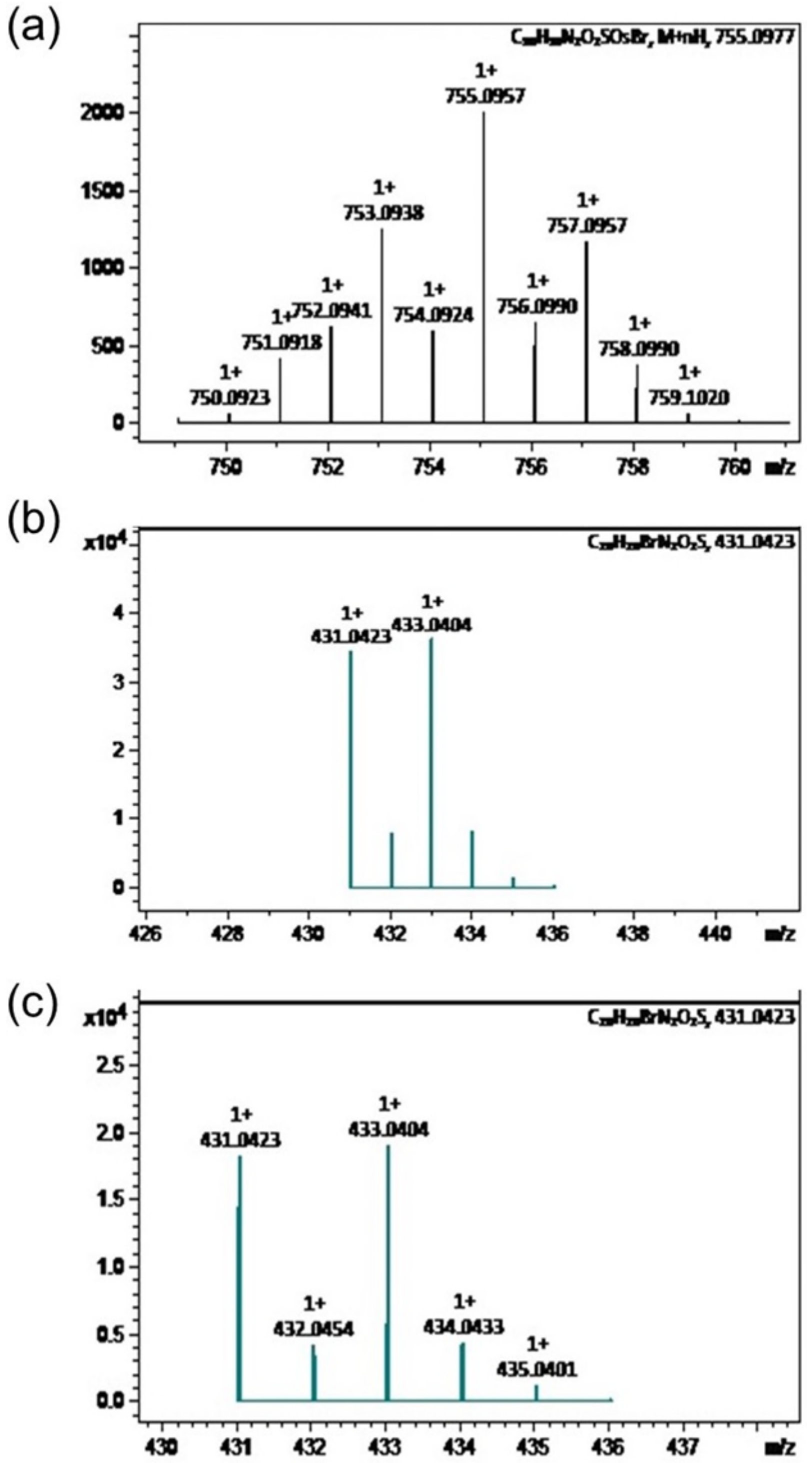

Figure S57. HR-MS of the solution resulting from incubating ***RR*-2** (MW = 753.78) at pH 5.5 (prepared in ammonium acetate) for 24 h in the presence of 0, 1 or 10 mM of L-cysteine. (a) 0 mM L-cysteine - ***RR*-1** remains intact in solution ($[C_{30}H_{31}BrN_2O_2OsS + H]^+$ = 755.0977). (b) 1 mM L-cysteine - ***RR*-2** dissociates in the presence of L-cysteine, releasing the BrPh-TsDPEN ligand in solution ($([C_{20}H_{19}BrN_2O_2S + H]^+$ = 431.0423). No peak at 755.0957 was observed. (c) 10 mM L-cysteine - ***RR*-2** dissociates in the presence of L-cysteine, releasing the BrPh-TsDPEN ligand in solution $[C_{20}H_{19}BrN_2O_2S + H]^+$ = 431.0423). No peak at 755.0957 was observed.

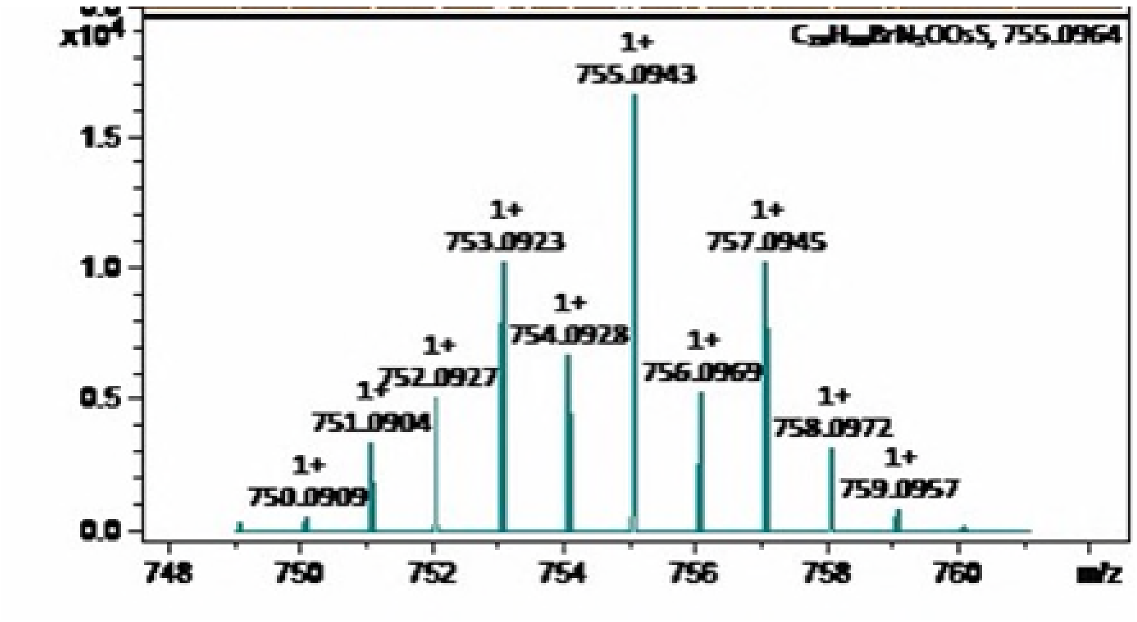

Figure S58. HR-MS of the of ***SS*-2** (MW = 753.78) at pH 7 (prepared in ammonium acetate) for 24 h in the presence of 0.2 mM cystine (approximate concentration in DMEM cell culture medium used in this work). ***SS*-2** remains intact in solution ($[C_{30}H_{31}BrN_2O_2OsS + H]^+$ = 755.0964).

ES16 L-BSO GSH depletion assay

The antiproliferative activity of ***RR*-1** in A2780 (human ovarian) cancer cells in the presence of a non-toxic concentration of 5 μM L-BSO (an inhibitor of γ-glutamylcysteine synthetase enzyme involved in the synthesis of glutathione) was determined using the general SRB assay (**Figure S59**).

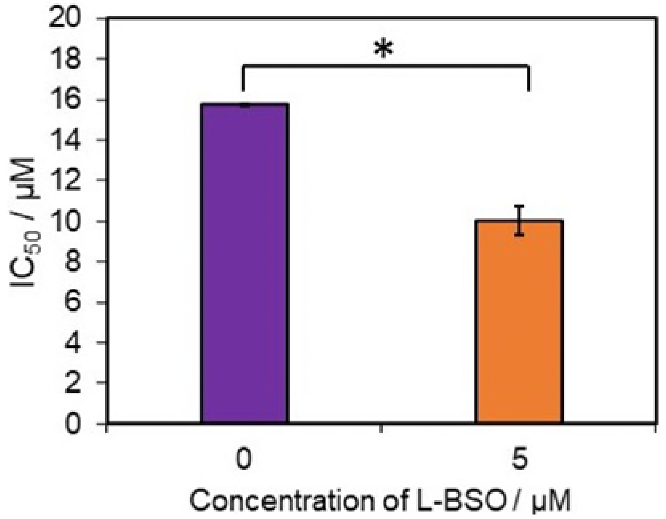

Figure S59. Modulation of the antiproliferative activity of complex ***RR*-1** by co-administration of a non-toxic concentration of L-BSO (5 μM), in A2780 (human ovarian) cancer cells for 24 h, followed by 72 h recovery (in complex free medium). A statistically significant decrease un IC_{50} of ***RR*-1** was observed upon co-administration with 5 μM of L-BSO (p=0.005).

ES17 Chloroquine-dependent *in vitro* activity

Chloroquine-dependent antiproliferative activity. Chloroquine toxicity was determined in A549 (lung) cancer cells using the general SRB protocol but treating cells with 0-200 µM of chloroquine diphosphate (prepared in DMEM) for different exposure times (0-10 h). The chloroquine-dependent IC_{50} concentrations of ***RR*-1** and ***SS*-2** were determined in A549 (lung) cancer cells using the general SRB protocol. Prior to treatment with ***RR*-1** or ***SS*-2**, cells were pre-incubated in 150 µM of chloroquine disphosphate (in DMEM) for 2 h. The chloroquine was removed, cells washed with PBS and then treated with 0.1-150 µM of ***RR*-1** or ***SS*-2.**

Chloroquine-dependent membrane integrity. The chloroquine-dependent effect on membrane integrity was investigated in A549 cells using the same membrane integrity protocol described previously. Cells were pre-incubated with 150 µM chloroquine disphosphate (DMEM) for 2 h, washed with PBS and treated with 1 × IC_{50} of ***RR*-1** (21 µM) or ***RR*-2** (30 µM) for 24 h (no recovery).

Table S27. The membrane integrity flow cytometry for a normalized population of A549 (human lung) cancer cells pre-incubated with either DMEM or chloroquine diphosphate (150 µM) for 2 h, followed by treatment with 1 × IC_{50} of ***RR*-1** (21 µM) or ***SS*-2** (30 µM) for 24 h (no recovery). Chloroquine did not alter the membrane viability in either the untreated controls or cells treated with ***RR*-1** or ***SS*-2.** Statistical significance was calculated using a two-tailed t-test assuming unequal variances (Welch's t-test) compared to the untreated controls, *$p < 0.05$, **$p < 0.01$ and ***$p < 0.001$.

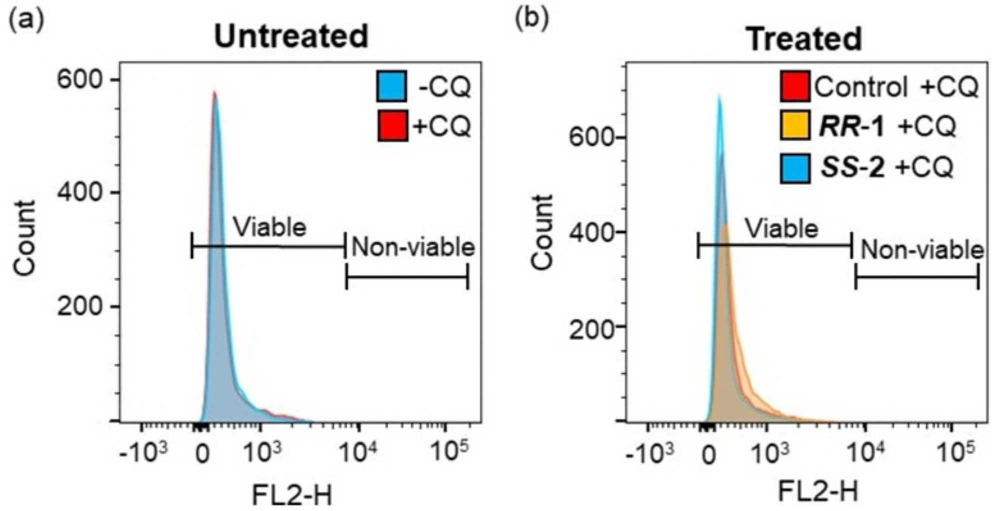

Complex	Viable membrane ($FL2^-$, %)	Non-viable membrane ($FL2^+$, %)
**Untreated control**	99.51±0.05	0.49±0.05
**Chloroquine control (150 µM)**	99.43±0.11	0.57±0.11
***RR*-1**	98.92±0.13	1.08±0.13
***RR*-1 + CQ**	98.78±0.16	1.22±0.16
***SS*-2**	98.31±0.02	1.69±0.02
***SS*-2 + CQ**	99.05±0.05	0.95±0.05

Chloroquine-dependent ^{189}Os and ^{79}Br cellular accumulation. The chloroquine-dependent cellular accumulation of osmium was determined for A549 (lung) cancer cells following the general accumulation protocol. Cells were pre-incubated with 150 µM chloroquine disphosphate (DMEM) for 2 h, washed with PBS and treated with 1 × IC_{50} of ***RR*-1** (21 µM) or ***SS*-2** (30 µM) for 24 h (no recovery). The experiment was performed twice for ***SS*-2** for HNO_3 and TMAH digestion.

Table S28. Osmium (^{189}Os) and bromine (^{79}Br) ICP-MS cellular accumulation (ng / 10^6 cells) and molar Br / Os ratios in A549 (human lung) cancer cells pre-incubated with 0 or 150 µM chloroquine and treated with 1 × IC_{50} (30 µM) of ***SS*-2** for 24 h (no recovery). Statistical analysis was performed using the Welch's two-tailed t-test, assuming unequal variances, *$p < 0.05$, **$p < 0.01$ and ***$p < 0.001$. A statistically significant decrease (*ca.* 29% decrease) in Br/Os was observed for cells incubated in chloroquine diphosphate prior to RR-2 treatment compared to the untreated controls (p=0.0001).

	***SS*-2**	***SS*-2 + CQ**
**Os (ng / 10^6 cells)**	42±5	57±5
**Br (ng / 10^6 cells)**	600±27	552±60
**Molar Br / Os**	34.8±0.1	23.2±0.2

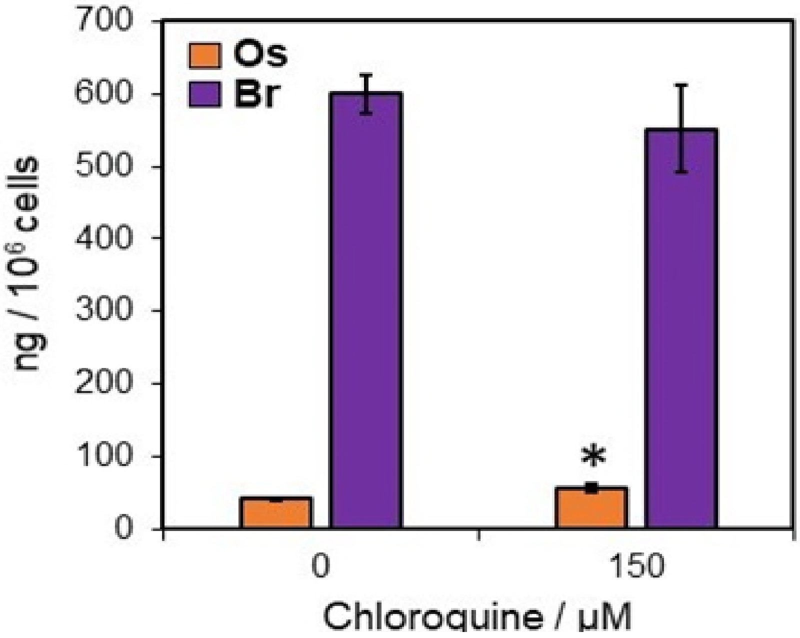

Figure S60. Accumulation of osmium (^{189}Os; ng / 10^6 cells) and bromine (^{79}Br; ng / 10^6 cells) in A549 cells pre-incubated with 0 or 150 µM chloroquine diphosphate for 2 h and treated with 1 × IC_{50} of ***RR*-2** (30 µM) for 24 h (no recovery). Statistical analysis was performed using the Welch's two-tailed t-test, assuming unequal variances, *$p < 0.05$, **$p < 0.01$ and ***$p < 0.001$.

Chloroquine-dependent formate cell viability. A549 cells were seeded as per the general SRB protocol. Cells were treated with 150 µM chloroquine disphosphate (DMEM) for 2 h, washed with PBS and treated with 1 × IC_{50} of ***SS*-2** (30 µM) co-administered with 0 or 2 mM sodium formate for 24 h, followed by 72 h recovery in complex-free DMEM.

Table S29. Transfer hydrogenation-modulated cellular viability of human A549 lung cancer cells pre-incubated with 0 or 150 µM chloroquine diphosphate for 2 h and treated with 1×IC_{50} of ***SS*-2** in the presence of sodium formate (0-2 mM). All cell viability experiments were performed as duplicates of triplicates. Statistical significance was calculated using a two-tailed t-test assuming unequal variances (Welch's t-test), *$p < 0.05$, **$p < 0.01$ and ***$p < 0.001$.

**Condition**	**Normalised cell viability (%)**
***SS*-2**	49±3
***SS*-2 + Chloroquine**	28±3
***SS*-2 + Formate**	18±3
***SS*-2 + Formate + Chloroquine**	10.7±2.5

ES18 References

[1] G. Kang, S. Lin, A. Shiwakoti, B. Ni, *Catal. Commun.* **2014**, *57*, 111–114.

[2] J. Tönnemann, J. Risse, Z. Grote, R. Scopelliti, K. Severin, *Eur. J. Inorg. Chem.* **2013**, 4558-4562.

[3] J. P. C. Coverdale, C. Sanchez-Cano, G. J. Clarkson, R. Soni, M. Wills, P. J. Sadler, *Chem. Eur. J.* **2015**, *21*, 8043-8046.

[4] J. P. C. Coverdale, I. Romero-Canelón, C. Sanchez-Cano, G. J. Clarkson, A. Habtemariam, M. Wills, P. J. Sadler, *Nat. Chem.* **2018**, *10*, 347.

[5] J. J. Wilson, S. J. Lippard, *J. Med. Chem.* **2012**, *55*, 5326-5336.

[6] P. Skehan, R. Storeng, D. Scudiero, A. Monks, J. McMahon, D. Vistica, J. T. Warren, H. Bokesch, S. Kenney, M. R. Boyd, *J. Natl. Cancer Inst.* **1990**, *82*, 1107-1112.

[7] J. P. C. Coverdale, H. E. Bridgewater, J.-I. Song, N. A. Smith, N. P. E. Barry, I. Bagley, P. J. Sadler, I. Romero-Canelón, *J. Med. Chem.* **2018**, *61*, 9246-9255.

[8] M. H. M. Klose, M. Hejl, P. Heffeter, M. A. Jakupec, S. M. Meier-Menches, W. Berger, B. K. Keppler, *Analyst* **2017**, *142*, 2327-2332; b) C. Venzago, M. Popp, J. Kovac, A. Kunkel, *J. Anal. At. Spectrom.* **2013**, *28*, 1125-1129.

[9] J. L. Gregg, K. M. McGuire, D. C. Focht, M. A. Model, *Pflügers Arch.* **2010**, *460*, 1097-1104.

[10] V. A. Solé, E. Papillon, M. Cotte, P. Walter, J. Susini, *Spectrochim. Acta B* **2007**, *62*, 63-68.

[11] C. T. Rueden, J. Schindelin, M. C. Hiner, B. E. DeZonia, A. E. Walter, E. T. Arena, K. W. Eliceiri, *BMC Bioinform.* **2017**, *18*, 529.

[12] a) V. Stoka, B. Turk, V. Turk, *IUBMB life* **2005**, *57*, 347-353; b) M. E. McGrath, *Ann. Rev. Bioph. Biom.* **1999**, *28*, 181-204.
